# Blood-brain-barrier spheroids as an *in vitro* screening platform for brain-penetrating agents

**DOI:** 10.1038/ncomms15623

**Published:** 2017-06-06

**Authors:** Choi-Fong Cho, Justin M. Wolfe, Colin M. Fadzen, David Calligaris, Kalvis Hornburg, E. Antonio Chiocca, Nathalie Y. R. Agar, Bradley L. Pentelute, Sean E. Lawler

**Affiliations:** 1 Harvey Cushing Neuro-Oncology Laboratories, Department of Neurosurgery, Brigham and Women’s Hospital, Harvard Medical School, Boston, Massachusetts 02115, USA; 2 Department of Chemistry, Massachusetts Institute of Technology, Cambridge, Massachusetts 02139, USA; 3 Department of Neurosurgery and Radiology, Brigham and Women’s Hospital, Harvard Medical School, Boston, Massachusetts 02115, USA

## Abstract

Culture-based blood–brain barrier (BBB) models are crucial tools to enable rapid screening of brain-penetrating drugs. However, reproducibility of *in vitro* barrier properties and permeability remain as major challenges. Here, we report that self-assembling multicellular BBB spheroids display reproducible BBB features and functions. The spheroid core is comprised mainly of astrocytes, while brain endothelial cells and pericytes encase the surface, acting as a barrier that regulates transport of molecules. The spheroid surface exhibits high expression of tight junction proteins, VEGF-dependent permeability, efflux pump activity and receptor-mediated transcytosis of angiopep-2. In contrast, the transwell co-culture system displays comparatively low levels of BBB regulatory proteins, and is unable to discriminate between the transport of angiopep-2 and a control peptide. Finally, we have utilized the BBB spheroids to screen and identify BBB-penetrant cell-penetrating peptides (CPPs). This robust *in vitro* BBB model could serve as a valuable next-generation platform for expediting the development of CNS therapeutics.

## Abstract (web summary)

*In vitro* blood-brain barrier (BBB) models are crucial tools for screening brain-penetrating compounds. Here the authors develop a self-assembling BBB spheroid model with superior performance to the standard transwell BBB model, and use their platform to identify cell-penetrating peptides that can cross the BBB.

The inability of most therapeutics to cross the blood–brain barrier (BBB) is a major limitation to effective treatment of diseases in the central nervous system. The BBB is a highly evolved microvasculature system comprised of brain endothelial cells (ECs) lining the vascular lumen, pericytes in the basal lamina, and associating astrocytic end-feet, microglia and neurons. This cellular architecture forms functional neurovascular units that regulate molecular trafficking between blood and brain^1^. Specific features of the brain endothelium distinguish it from the peripheral endothelium, including the presence of complex tight junctions that restrict paracellular permeability^2^, efflux pumps (for example, P-glycoprotein (P-gp)) that move foreign substances out of the brain^3^, and specific transporters and receptors (such as glucose carriers and amino acid carriers) to supply the brain with essential nutrients and other molecules^4^,^5^.

Systems to model the BBB *in vitro* are crucial tools to help predict brain uptake of drug candidates before costly and laborious *in vivo* studies. One major challenge in developing representative models of the BBB is the fact that brain ECs rapidly dedifferentiate and lose their BBB characteristics when they are removed from their native microenvironment and grown in culture, giving rise to a more generic EC phenotype^6^. This phenomenon, known as ‘phenotypic drift’, is especially likely to occur in ECs at higher passages, resulting in lack of expression of key BBB modulators and leaky paracellular barrier function^7^. It has been reported that direct cell–cell interaction of brain ECs with other components of the neurovascular unit such as pericytes and/or astrocytes is important for induction and maintenance of the specialized BBB properties in culture^1^,^8^. This has led to the development of several *in vitro* BBB co-culture models with this modular organization to facilitate discovery and advancement of neuropharmaceuticals^9^,^10^.

To date, the transwell system is the simplest and most widely used *in vitro* BBB model, in which ECs are plated on the upper (apical ‘blood’ side) on a semipermeable membrane, separated from the lower (basal ‘brain’ side) compartment. While this mid- to high-throughput model offers ease of culture and versatility^11^, it has been criticized owing to several well-known limitations^12^. Efforts to simulate a more realistic representation of the BBB morphology in a living system by reproducing the microcirculatory environment in the brain to account for blood flow and shear stress have led to the development of the hollow fibre (dynamic *in vitro* BBB) model^13^ and other microfluidic BBB systems^14^,^15^,^16^. However, these devices are also limited in terms of throughput, and their construction is rather complex, making them relatively inaccessible to many laboratories.

Human brain ECs, pericytes and astrocytes spontaneously form into a multicellular spheroid in co-culture under low-adherence conditions, and self-assemble into a modular organization that resembles the BBB (ref. 17). An important feature of this platform is that each cell type is able to interact with one another within the spheroid, which has been reported to play an essential role in the maintenance of the BBB integrity and function^9^. We present here a modified approach in culturing multicellular BBB spheroids to recapitulate key barrier functions that represent the BBB, and for the first time, demonstrate the utility of this model as a reliable and predictive *in vitro* screening tool for BBB-penetrating compounds. We show that the surface of the BBB spheroids is regulated by the activity of key BBB modulators, such as tight/adherens junctions and the P-gp efflux pump. Furthermore, the expression level of these modulators is significantly higher on the surface of the BBB spheroids compared to ECs grown using the transwell approach. As a proof-of-principle, we also demonstrate the versatility of this model in analysing the LRP-1 receptor-mediated transport of angiopep-2 (refs 18, 19), as well as the permeability of a BBB-penetrant small molecule that inhibits the phosphatidylinositol 3-kinase inhibitor, known as BKM120 (buparlisib)^20^. Finally, we have utilized the BBB spheroid platform to screen and identify cell-penetrating peptides (CPPs) with the ability to cross the BBB. Given the facile construction and the ability of the BBB spheroids to reproduce key barrier activity and characteristics, this model is particularly attractive for cost-effective and high-throughput drug permeability testing.

## Results

### Cellular organization of BBB spheroids

To investigate the spontaneous assembly and formation of the BBB spheroids, we co-cultured primary human astrocytes and human brain vascular pericytes (HBVP) with two different human brain EC types: (1) primary human brain microvascular ECs (HBMECs) and (2) immortalized hCMEC/D3 (human cerebral microvascular EC line D3). First, we confirmed the identity of each cell type by immunofluorescence staining using cell-specific biomarkers; astrocytes were tested for glial fibrillary acidic protein (GFAP) expression (Supplementary Fig. 1a), pericytes for NG2 expression (Supplementary Fig. 1b), primary HBMECs for CD31 (Supplementary Fig. 1c) and immortalized hCMEC/D3 ECs for CD31, VE-cadherin and von Willebrand factor expression (Supplementary Fig. 1d,e). To produce a single spheroid, 1 × 10^3^ cells of each cell type were used. Astrocytes and pericytes were co-cultured with either EC type in a 1:1:1 ratio (as per Urich *et. al.*^17^) in endothelial basal media containing human serum on low-attachment culture plates coated with soft agarose. However, unlike the methodology described by Urich *et al*., we cultured the spheroids in media with no additional VEGF-A, as VEGF-A has been shown to induce vascular permeability through the disruption of tight junctions^21^ and break down of the BBB^22^,^23^.

When the three cell types were co-cultured, we found that all cells interacted tightly with one another and eventually self-assembled into a compact spheroid by 12 h (data not shown). Out of a total of 886 spheroids, 797 of the spheroids formed after 48 h of co-culture were considered ‘acceptable’ on the basis of their physical characteristics (Supplementary Fig. 2a; *n*_experiment_=5; 184/230, 117/136, 85/90, 205/215 and 206/215), yielding an average success rate of 90%. Long-term analysis of spheroid size showed that the spheroids remained stable and viable for over 17 days in culture (Supplementary Fig. 2b).

**Figure 1:**
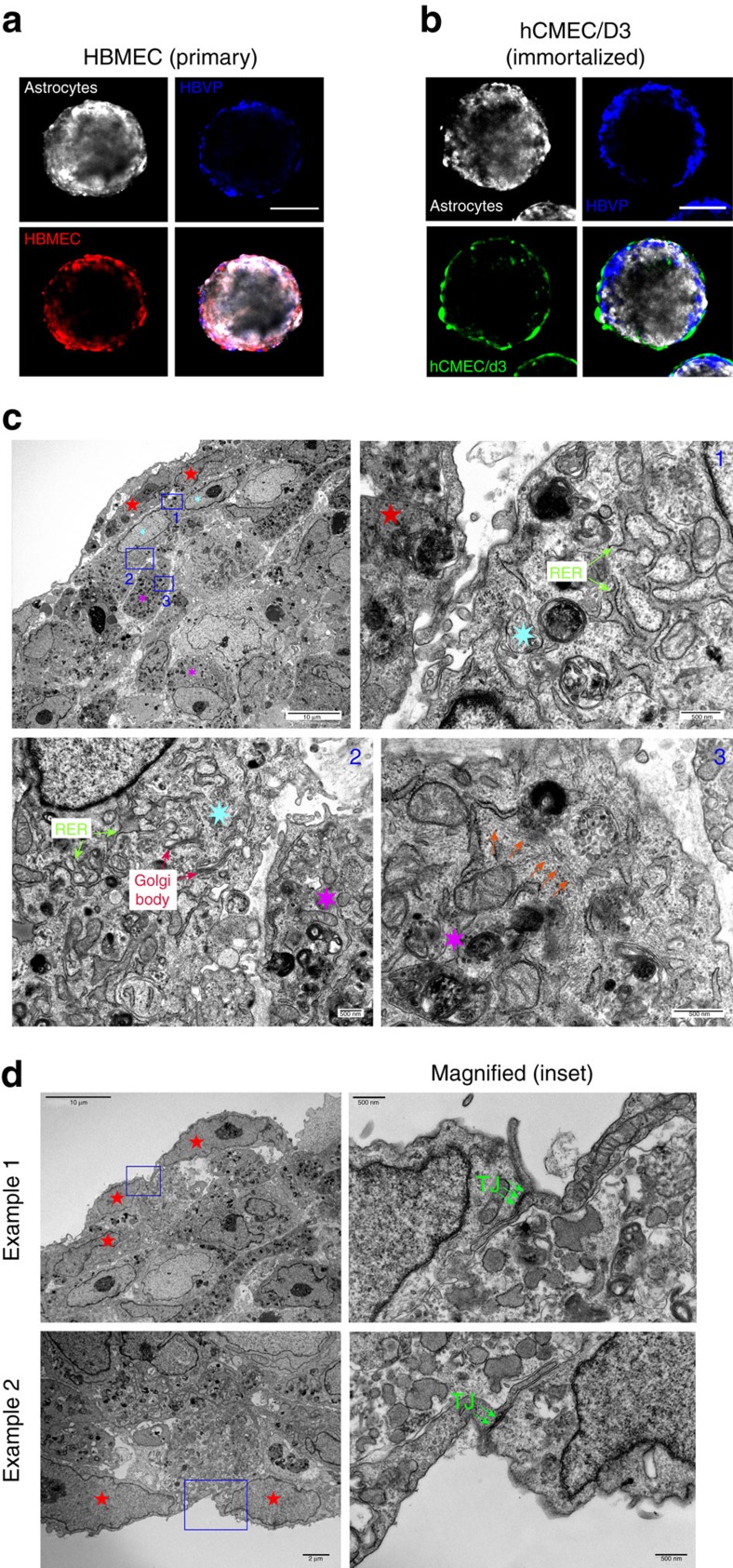
Cellular organization of the multicellular BBB spheroids. Representative confocal images showing the organization of human astrocytes (white), HBVP (blue) and (**a**) primary HBMEC (red) or (**b**) immortalized human cerebral microvascular ECs (hCMEC/D3; green) when co-cultured to form a spheroid. Astrocytes were pre-labelled with VivoTrack 680 Fluorescent Imaging Agent, HBVP with CellTracker Violet, HBMEC with CellTracker Orange, and hCMEC/D3 with CellTracker Green for 1 h before co-culturing on 1% agarose for 48 h. Spheroids were imaged using confocal microscopy, and images displayed represent the organization of each cell type at 96 μm depth from the surface of the spheroid. Scale bar, 100 μm; *n*_spheroid_=5. (**c**) TEM images showing the organization of hCMEC/D3 ECs, HBVP and astrocytes within a spheroid. ECs are identified from their elongated cellular form and irregularly shaped nuclei (red stars), while HBVP (cyan stars) are distinguished based on their elongated rough endoplasmic reticulum (RER) and well-developed Golgi body (see magnified inset 1 and 2; scale bar, 500 nm). Astrocytes (magenta stars) are characterized by the presence of filamentous bundles (orange arrows) within the cell (see inset 3; scale bar, 500 nm). Scale bars are indicated within each image. (**d**) Representative TEM images showing the presence of tight junctions (green arrows) between ECs (red stars) that lined the surface of the spheroid. Scale bar, 2 μm.

**Figure 2:**
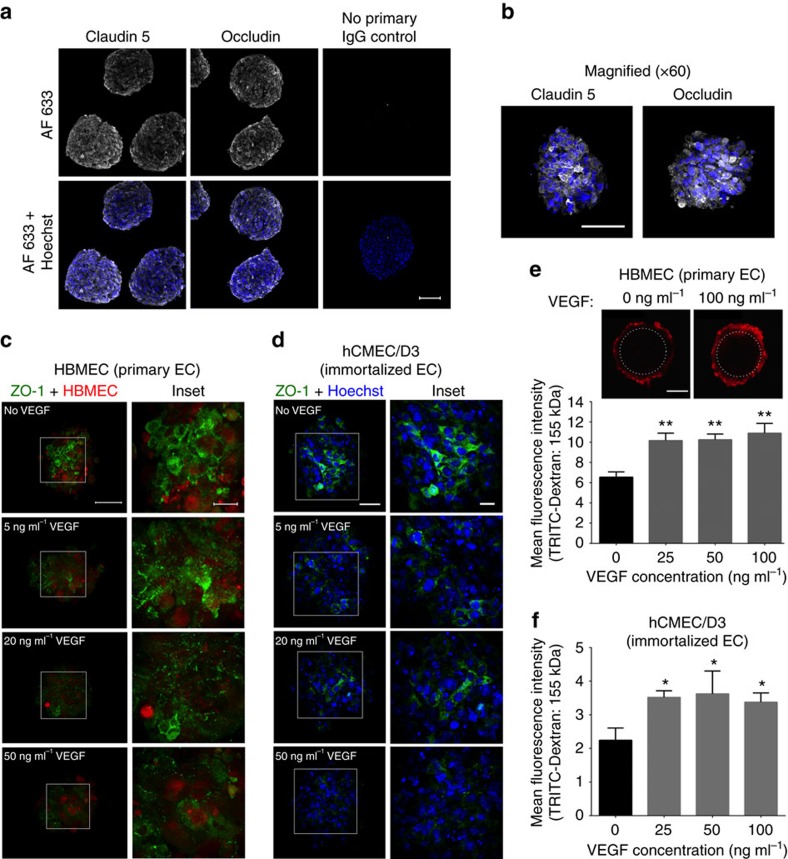
Surface permeability of BBB spheroid to high molecular weight dextran and intact tight junctions are modulated by VEGF. (**a**) Fluorescence images showing the expression of tight junction markers, claudin 5 and occludin (white). Nuclei of spheroids were stained with Hoechst dye (blue). Scale bar, 100 μm (× 20 objective). (**b**) Magnified fluorescence images showing claudin 5 and occluding expression. Scale bar, 100 μm (× 60 objective). (**c**) Fluorescence images showing decreased expression of tight junction marker (ZO-1: green) with increasing VEGF-A concentration (at 5, 20 and 50 ng ml^−1^) in primary HBMEC (pre-labelled with CellTracker Red dye) and (**d**) immortalized hCMEC/D3 ECs. Cell nuclei were labelled with Hoechst dye (blue). Scale bar: 50 μm in lower-magnification images; 10 μm in magnified images. (**e**) Dextran permeability assay showing that VEGF-A (at 25, 50 and 100 ng ml^−1^) increased spheroid permeability to TRITC-Dextran (155 kDa; red; 10 mg ml^−1^) using spheroids established using primary HBMEC ECs. The image panels above the graph depict a representation of how permeability was assessed. The white dotted line marks the area within the core of the spheroid, where the mean fluorescence intensity was quantified. Scale bar, 50 μm; *n*=8. (**f**) Dextran permeability study (as in **e**) using spheroids established using immortalized hCMEC/D3 ECs. n_spheroid_=3–5. Both graphs show mean TRITC fluorescence intensity quantified at 88 μm depth from the surface of the spheroid with s.d. error bars (**P<0.01; *P<0.05). Statistical analyses were performed using the one-way ANOVA and Dunnett’s multiple comparison test.

To distinguish each cell type and track structural organization in co-culture, we pre-labelled each of them with a distinct fluorescent long-term cell-labeling dye as follows: astrocytes (VivoTrack 680 Near-Infrared), pericytes (CellTracker Violet), primary HBMECs (CellTracker Orange) and immortalized hCMEC/D3 (CellTracker Green). After 48 h of co-culturing, we observed distinct cell–cell stratification within the spheroid by confocal microscopy. Consistent with observations by Urich and colleagues^17^, astrocytes were seen to mostly occupy the spheroid core, while HBMECs or hCMEC/D3 ECs together with pericytes appeared to form a surface monolayer encasing the spheroid (Fig. 1a,b). This modular organization was observed in both the primary and the immortalized ECs. Furthermore, we also utilized transmission electron microscopy (TEM) to gain deeper insight into the ultrastructure of each spheroid. Consistent with our prior observations using confocal microscopy, we saw that hCMEC/D3 ECs (characterized by the nature of their irregularly shaped nuclei and elongated cellular structure^24^) lined the outer surface of the spheroid (Fig. 1c). Pericytes (marked by the presence of elongated rough endoplasmic reticulum and well-developed Golgi body^25^) formed direct contact with ECs close to the spheroid surface (Fig. 1c). Meanwhile, astrocytes (characterized by an abundance of filamentous bundles within the cell^26^) were found mostly in the spheroid’s interior (Fig. 1c). A detailed analysis at the point of cell–cell contact between ECs on the surface of each spheroid revealed the formation of dense strands of tight junctions (Fig. 1d), indicating that the outer surface could act as a barrier in governing transport into the spheroid. Indeed, we confirmed a high level of tight junction proteins, including occludin, claudin 5 and zonula occludens-1 (ZO-1) at the surface of each spheroid (Fig. 2a–c).

### Molecular trafficking is regulated by BBB mechanisms

To test if the surface of the BBB spheroid could serve as a functional ‘barrier’ in regulating molecular traffic between the spheroid and its environment, we investigated the activity of key BBB modulators on the spheroid surface. Although small gaseous molecules such as oxygen and carbon dioxide, as well as small lipophilic drugs such as ethanol can diffuse freely across the BBB, complex tight junctions between adjacent brain ECs create a physical barrier to prevent most molecules from entering the brain via the paracellular route^1^. Indeed, we observed that the surface of BBB spheroids displayed low permeability to high molecular weight dextran in spheroids established with primary or immortalized ECs (Fig. 2e,f). Consistent with other reports^21^,^23^, immunofluorescence staining of the tight junction protein ZO-1 confirmed disruption of tight junctions on the spheroid surface in the presence of increasing vascular endothelial growth factor A (VEGF-A) concentrations (Fig. 2c,d). As expected, we also saw a corresponding influx of dextran into the spheroids as VEGF-A concentration increases (Fig. 2e,f and Supplementary Fig. 3).

**Figure 3:**
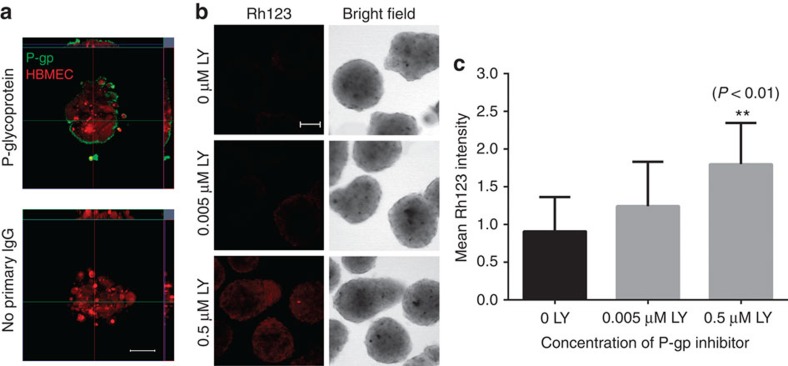
Function of efflux pump, P-gp on multicellular BBB spheroids. (**a**) Immunofluorescence images showing the expression of efflux pump, P-gp (green) on the surface of the spheroid after 48 h of co-culture. HBMECs (red) were pre-labelled with CellTracker Orange before spheroid formation. Scale bar, 50 μm. (**b**) Fluorescence images acquired using confocal microscopy showing that increasing concentration of a P-gp inhibitor, LY335979 increases influx of rhodamine 123 (Rh123; 0.5 μg ml^−1^), a substrate of P-gp into the spheroids. Scale bar, 100 μm. (**c**) Bar graph depicting the influx of Rh123 into the spheroid with increasing LY335979 concentration. The graph shows the mean rhodamine fluorescence intensity quantified from images in **b**, with s.d. error bars (**P<0.01; *n*_spheroid_=11). Statistical analyses were performed using the one-way ANOVA and Tukey’s multiple comparison test.

Next, we chose to examine the BBB spheroids for efflux pump activity, which is responsible for the active transport of a variety of drugs out of the brain capillary ECs^9^. First, we showed that each spheroid has a high level of P-gp efflux pump expression (Fig. 3a). Treatment of spheroids with a potent P-gp inhibitor (LY335979)^27^ resulted in a significant influx of rhodamine 123 (Rh123), a substrate of P-gp, into the spheroid (Fig. 3b,c). Together, these data suggest that the surface of each multicellular spheroid forms an impervious barrier displaying fundamental BBB functions to strictly regulate access of most foreign molecules into the spheroid.

**Figure 4:**
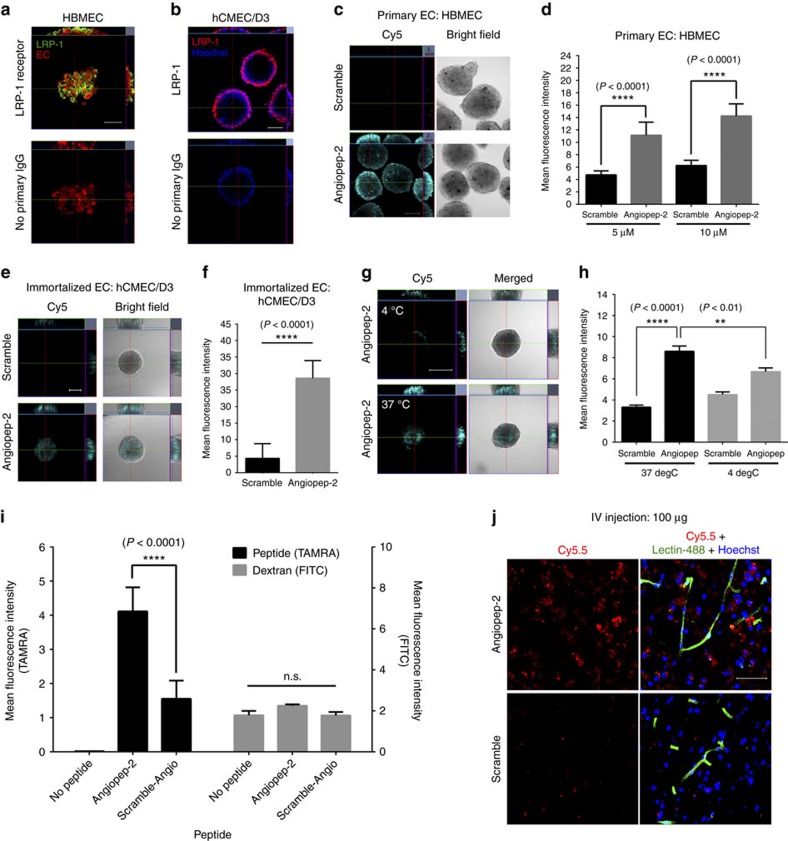
Analysis of angiopep-2 transport in BBB spheroid. (**a**) Fluorescence images showing LRP-1 receptor expression (green) in spheroids established with primary HBMECs (pre-labelled in CellTracker Orange (shown in red)). Scale bar, 50 μm. (**b**) Fluorescence images showing the LRP-1 receptor expression (red) in immortalized hCMEC/D3 ECs. Nuclei of spheroids were stained with Hoechst dye (blue). Scale bar: 100 μm. (**c**) Confocal fluorescence images showing the transport of Cy5-labelled angiopep-2 (cyan; compared to a corresponding scrambled peptide) in spheroids established with primary HBMECs. Spheroids were incubated with either angiopep-2 or scrambled-Cy5 peptide (10 μM) at 37 °C for 3 h. Scale bar, 100 μm. (**d**) Bar graph quantifying the transport of angiopep-2 (or scrambled peptide) at a concentration of 5 and 10 μM in spheroids established with primary HBMECs. Statistical analyses were performed using the one-way ANOVA and Bonferroni’s multiple comparison test. (**e**) Fluorescence images showing the transport of Cy5-labelled angiopep-2 (cyan; conducted as in **c**) in spheroids established with immortalized hCMEC/d3 cells. Scale bar, 100 μm. (**f**) Bar graph quantifying the transport of angiopep-2 (10 μM; from **e**). Statistical analyses were performed using the Student’s *t*-test. (**g**) Fluorescence images acquired using confocal microscopy showing the transport of Cy5-labelled angiopep-2 (cyan; 10 μM) in spheroids established with primary HBMECs at either 4 °C (to inhibit endo/transcytosis) or 37 °C for 3 h. Scale bar, 200 μm. (**h**) Bar graph quantifying the transport of angiopep-2 at either 4 or 37 °C (from **g**). Statistical analyses were performed using the one-way ANOVA and Tukey’s multiple comparison test. All graphs above depict mean Cy5 intensity quantified at 88 μm depth from the surface of the spheroid with s.d. error bars (*n*_spheroid_=10, *n*_experiment_=3). (**i**) Co-incubation of spheroids with TAMRA-labelled angiopep-2 or angio-scramble (at 10 μM) and with FITC-dextran (70 kDa; at 10 μg ml^−1^) for 3 h. The graph displays the mean fluorescence intensity of the peptides (TAMRA) on the left *y* axis, and dextran (FITC) on the right *y* axis at 88 μm depth from the surface of each spheroid with s.d. error bars (*n*_spheroid_=3–6, *n*_experiment_=3). Incubation of spheroids with each peptide did not increase spheroid permeability to FITC-dextran. Statistical analyses were performed using the two-way ANOVA and Dunnett’s multiple comparison test. (**j**) Fluorescence images of brain cryosections showing the accumulation of angiopep-2 (red) in the brain tissue compared to the scrambled peptide. Angiopep-2 (or the scrambled peptide; 100 μg) were injected via the tail vein. Mice were killed after 24 h, and the brains were excised. The vasculature was stained with DyLight 488 lectin (green), while cell nuclei were labelled with Hoechst dye (blue). Scale bar, 50 μm.

As a proof-of-concept to investigate active transport across the spheroid surface, we employed the brain-penetrant agent angiopep-2, a 19-residue peptide known to cross the BBB via receptor-mediated transcytosis through the low-density lipoprotein receptor-related protein-1 (LRP-1) receptor^18^,^19^. We confirmed a high level of LRP-1 receptor expression on the surface of spheroids established using both the primary HBMECs and immortalized hCMEC/D3 cells (Fig. 4a,b). Angiopep-2 and a corresponding scrambled sequence of angiopep-2 were labelled with either a Cy5 or rhodamine dye for detection by confocal microscopy. The ability of a given compound to penetrate the surface barrier of the spheroid is represented by the fluorescence intensity at a given depth below the spheroid surface (up to 100 μm tissue penetration depth limit of the confocal microscope). We saw a substantial influx of fluorescently labelled angiopep-2 compared with the scrambled control for both spheroids prepared with HBMECs or hCMEC/D3 cells (Fig. 4c–f, and Supplementary Fig. 4a). A time-course analysis further validated the transport of angiopep-2 into the spheroid (Supplementary Fig. 4b,c). The scrambled peptide displayed a dramatic reduction in permeability (Supplementary Fig. 4b,c), while the spheroids remained completely impermeable to unconjugated rhodamine dye and rhodamine-dextran (Supplementary Fig. 4c). To further verify our observations from optical sectioning microscopy, BBB spheroids incubated with angiopep-2 (or the scrambled control) were fixed and cryosectioned to expose the core. Fluorescence microscopy revealed a high level of angiopep-2 in the core of the spheroid, whereas a negligible amount of the scrambled peptide was detected (Supplementary Fig. 4d). Both peptides exhibited a similar stability profile in the working media (containing 2% human serum), supporting the notion that the observed increase in angiopep-2 uptake into the spheroids was not related to peptide stability (Supplementary Fig. 4e). When the spheroids were incubated at 4 °C to inhibit transcytosis, the influx of angiopep-2 was significantly reduced (Fig. 4g,h), providing further evidence to support that angiopep-2 transport into the spheroids was indeed due to receptor-mediated transcytosis. To ensure that neither peptide would disrupt the surface barrier, spheroids were co-incubated with each peptide and a high molecular weight dextran. While we observed significantly higher influx of angiopep-2 compared with the scrambled peptide, the spheroid remained impermeable to dextran (Fig. 4i), thus verifying that angiopep-2 was transported into the spheroid across an intact surface barrier. We confirmed the ability of Cy5.5-labelled angiopep-2 to cross the BBB *in vivo* by demonstrating the extravasation and accumulation of angiopep-2 in the brain parenchyma in contrast to its scrambled counterpart, which showed negligible parenchymal accumulation (Fig. 4j).

Next, we tested the delivery of angiopep-2 conjugates of various sizes across the spheroid surface barrier. First, we conjugated TAMRA-labelled angiopep-2 (2.7 kDa) to a peptidic analogue of the Bim BH3 domain or to an affibody derived from a binding domain to the HER2/Neu receptor, yielding constructs with total molecular weights of 6.6 and 8.9 kDa, respectively (Supplementary Fig. 5a–i,b–i,c–i). In these cases, the influx of angiopep-2 conjugates into the spheroids was significantly higher compared to those of the scrambled peptide (Supplementary Fig. 5a–c). Angiopep-2 conjugated to the Bim BH3 peptide analogue had similar influx rate as unconjugated angiopep-2 (Supplementary Fig. 5b–iii), while the level of influx of angiopep-2 conjugated to an affibody was slightly lower than unconjugated angiopep-2, though significantly higher than that of the control affibody (Supplementary Fig. 5c–iii). We also observed that angiopep-2 was able to successfully transport GFP (∼30 kDa) into the spheroid (Supplementary Fig. 5d). Together, these results highlight the versatility of the BBB spheroid model in analysing the delivery of diverse cargoes and its potential for screening a wide range of therapeutics.

### Evaluation of small molecule transport using MALDI-MSI

**Figure 5:**
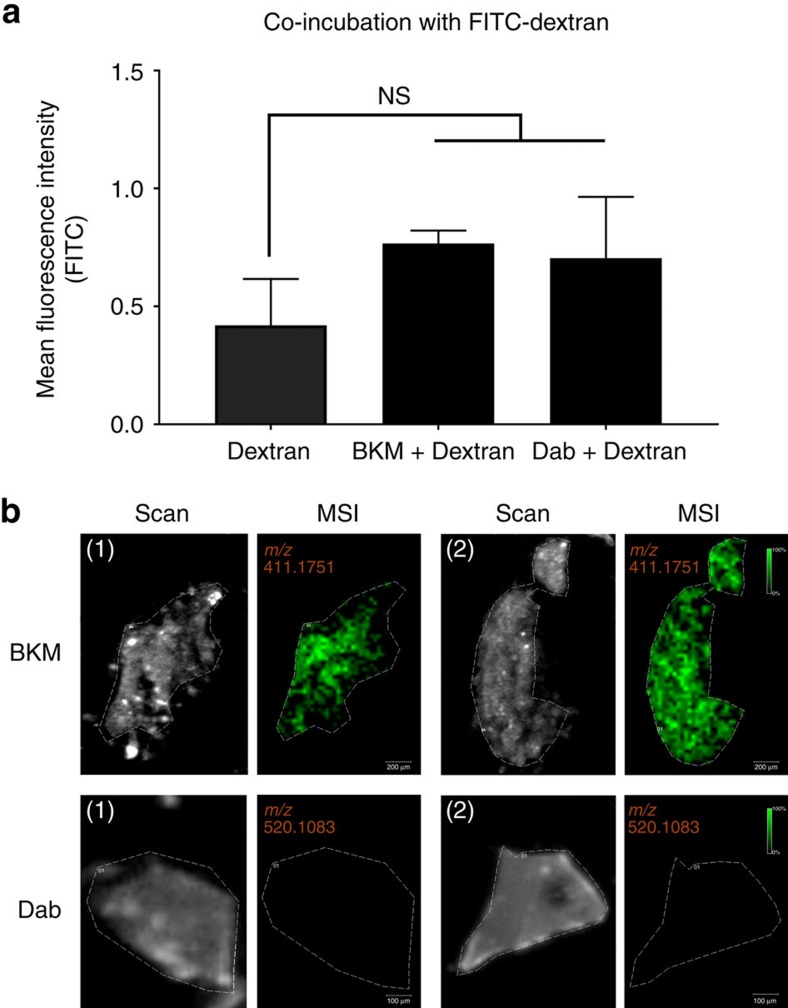
Transport of BKM120 (a BBB-penetrant drug) and dabrafenib (a non-penetrant drug) into BBB spheroids. (**a**) Co-incubation of spheroids with BKM120 or dabrafenib (at 10 μM concentration) and with FITC-dextran (70 kDa; at 10 μg ml^−1^ concentration) for 24 h did not affect spheroid’s surface integrity. The graph displays the mean fluorescence intensity of dextran (FITC) at 88 μm depth from the surface of each spheroid with s.d. error bars (*n*_spheroid_=3–6). Statistical analyses were performed using the one-way ANOVA and Tukey’s multiple comparison test. (**b**) MALDI-MSI ion images of sections of BBB spheroids incubated with BKM120 or dabrafenib (*n*_spheroid_=150, *n*_tissue_=2). Top panels show the distribution of BKM120 (in green, *m/z* 411.1751±0.001). Lower panels indicate that dabrafenib (*m/z* 520.1083±0.001) was not detected in the BBB spheroids. Dashed lines on the scanned images delineate the positions of the BBB spheres tissue sections. Scale bars are indicated within each image.

Because most drugs are non-fluorescent, we explored an alternative strategy to detect drugs within the BBB spheroids using matrix-assisted laser desorption/ionization mass spectrometry imaging (MALDI-MSI). As a proof-of-principle, we chose BKM120, a phosphatidylinositol 3-kinase inhibitor which is known to cross the BBB^20^,^28^, as well as dabrafenib, an inhibitor of the threonine-protein kinase B-Raf (V600E) that is known to have limited BBB penetrance^29^. First, we demonstrated that both BKM120 and dabrafenib did not affect the ability of BBB spheroids to restrict the entry of high molecular weight dextran (Fig. 5a), suggesting that the integrity of the spheroid’s surface barrier remained intact in the presence either drug for 24 h at 10 μM concentration. Next, BBB spheroids incubated with 10 μM of either BKM120 or dabrafenib (for the same duration) were frozen and then cryosectioned. Sectioning of the spheroid was performed to allow discernment of true drug penetration/accumulation inside the spheroid from endothelial uptake at the surface of the spheroid. For MALDI-MSI analyses, the tissue sections of the BBB spheroids were scanned and MS data were acquired at a spatial resolution of 30 μm (Supplementary Fig. 6a–c). Considering that an average spheroid had a diameter of ∼300 μm, a pixel step size of 30 μm across the tissue allowed us to properly assess MS information inside the spheroids to confirm drug penetration. Indeed, we observed a dramatic accumulation of BKM120 (*m/z* 411.1751±0.001) in the BBB spheroids, while the presence of dabrafenib (*m/z* 520.1083±0.001) was not detected (Fig. 5b).

### Comparison of BBB spheroid platform with transwell model

Because the transwell system is the most commonly used and convenient approach currently available for studying BBB transport, we compared its utility with the BBB spheroid platform. It has been reported that a triple co-culture of ECs with pericytes and astrocytes significantly improved the morphological and functional integrity of paracellular barriers and efflux pump expression/activity in the transwell model compared to when ECs were cultured alone, or in co-culture with either pericytes or astrocytes only^8^. In our studies, we co-cultured 5 × 10^7^ hCMEC/D3 on the apical side of the insert, 5 × 10^4^ pericytes on the basal side of the insert and 5 × 10^4^ astrocytes on the culture plate in the basal compartment (as shown in Supplementary Fig. 7a). After 84 h, an average trans-endothelial electrical resistance (TEER) value of ∼50 Ω cm^2^ was achieved in our triple co-culture transwell model (consistent with previously reported measurements^14^,^30^,^31^), and this value was significantly higher than values obtained with the endothelial mono-cultures, as expected (Supplementary Fig. 7b). Indeed, the low TEER value, even in our triple co-culture transwell model was expected, as immortalized ECs, such as hCMEC/D3 are known to have limited ability in forming restrictive monolayers *in vitro* and face many challenges in establishing BBB models^31^,^32^. Nonetheless, these immortalized ECs offer a feasible solution to the scarcity, cost and instability of primary human ECs, making them more practical for wide-scale studies^30^. The utility of human cells in our BBB model would allow us to avoid interspecies variations in screening for clinically relevant translatable agents. Alternatively, we also expect this model to be compatible with and easily adapted to integrate commonly used ECs from various other species (such as, mouse, bovine or porcine) for BBB studies^9^. Indeed, these ECs have been shown to yield higher TEER values in *in vitro* BBB models compared to human brain ECs^10^, though a major drawback includes the difference in the expression of various drug transporters and efflux pumps in animal ECs compared to human ECs^31^,^33^.

**Figure 6:**
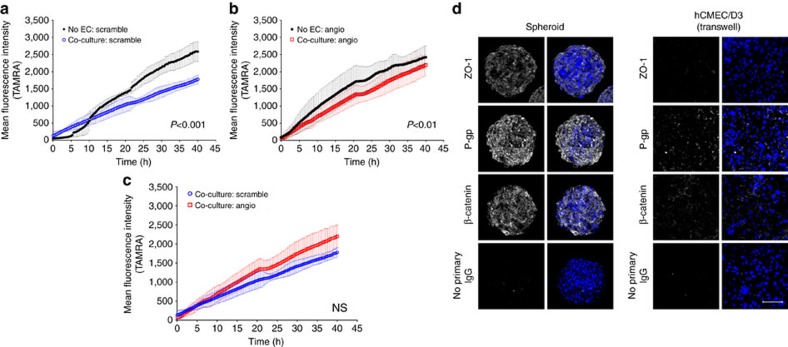
Analysis of angiopep-2 transport using the well-established *in vitro* BBB Transwell system. Permeability assay using the BBB co-culture Transwell model showing that the (**a**) scrambled control and (**b**) angiopep-2 displayed significantly lower permeation in the co-culture model compared to inserts containing no cells (which represent passive diffusion). (**c**) The Transwell co-culture model failed to differentiate between the permeability of angiopep-2 and the scrambled peptide. For all permeability assays, TAMRA-labelled angiopep-2 (or scramble) peptide (10 μM concentration) was added onto the apical side of the Transwells of the co-culture model after 84 h of incubation. The basal side of the Transwell was imaged using fluorescence microscopy, and the fluorescence intensity was quantified over 40 h. The plots show the accumulation of fluorescence intensity over time with s.d. error bars (*n*_transwell_=2, *n*_experiment_=2). Statistical analysis was performed using the one-way ANOVA and Tukey’s multiple comparison test. (**d**) Confocal images showing higher expression of ZO-1 (tight junction), P-gp (efflux pump) and β-catenin (adherens junction; shown in white) on the surface of BBB spheroids compared with hCMEC/D3 ECs in the triple co-culture Transwell model after 48 h. Cell nuclei were labelled with Hoechst dye (shown in blue). Scale bar, 100 μm.

Next, we used our triple co-culture transwell model to compare the permeability of angiopep-2 with the scrambled control peptide. We observed that compared to inserts with no ECs (which represent passive diffusion of compounds across the insert with no barrier), both angiopep-2 and the scrambled peptide displayed significantly lower permeability in the co-culture system (Fig. 6a,b). However, the transwell co-culture system was unable to differentiate between the permeability of angiopep-2 and the scrambled control (Fig. 6c). Interestingly, we found the level of P-gp, ZO-1 and β-catenin (adherens junction protein) on the surface of BBB spheroids to be substantially higher than ECs cultured in the triple co-culture transwell model (Fig. 6d). These results may be attributed to the fact that although the transwell co-culture models have been shown to improve barrier tightness^8^,^9^,^12^, this system, unlike the BBB spheroid model, fails to account for 3D cellular organization and direct cell–cell contact, which are imperative for proper cell differentiation and barrier formation^17^. In addition, it has also been reported that ECs tend to distribute unevenly on the transwell filter, resulting in barrier imperfections caused by the formation of multiple layers of cells or holes within the monolayer^34^. Furthermore, in terms of cost-effectiveness and versatility, a standard transwell system (utilizing a 24-well plate) typically requires a relatively large number of ECs (ranging between 10^4^–10^7^ cells) to form a tight cellular monolayer on the membrane surface over 3–7 days. Meanwhile, the spheroid model which only requires 1 × 10^3^ of each cell type (ECs, pericytes and astrocytes) consistently produced an intact and functional barrier displaying key BBB characteristics within 48 h. Altogether, these attributes highlight the spheroid model as highly cost-effective and reproducible, with substantial potential in facilitating high-throughput and robust screening of brain-penetrating drugs.

### Discovery of brain-penetrating CPPs using BBB spheroids

**Figure 7:**
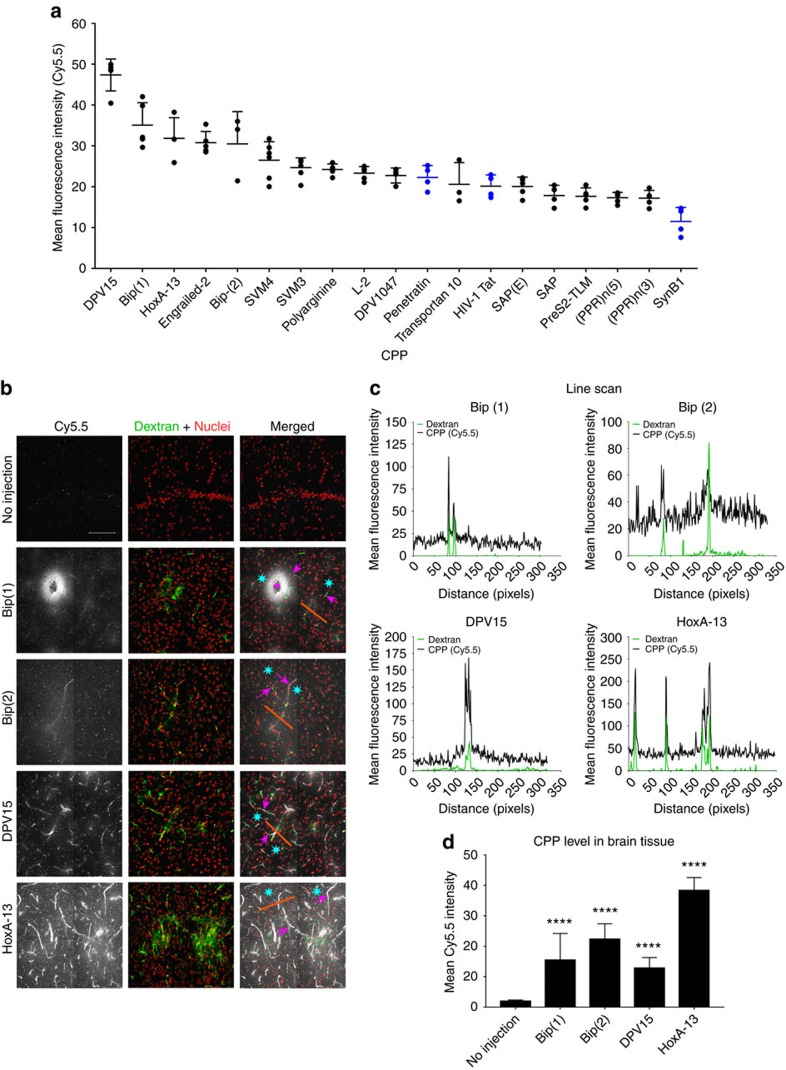
Screen of CPPs for BBB penetration using the multicellular BBB spheroids. (**a**) Waterfall plot showing the mean fluorescence intensity at 88 μm depth from the surface of each spheroid after incubation with each CPP at 5 μM concentration for 3 h. The CPPs were synthesized with Cy5.5 conjugated to the N-terminus. The graph displays s.d. error bars (*n*_spheroid_=3–7, *n*_experiment_=3). The HIV-1 Tat, penetratin and SynB1, (all well-established BBB-penetrating CPPs) are indicated in blue. (**b**) Fluorescence images of brain cryosections (20 μm slices) showing the localization of top 4 CPPs (shown in white) identified from **a** in the mouse frontal lobe. CPPs (100 μl of 500 μM peptide solution) were injected via the tail vein. 30 min later, mice were injected with 100 μl of 50 mg ml^−1^ of TRITC-dextran (155 kDa; shown in green). After 15 min, mice were euthanized, and their brains excised, flash frozen and cryosectioned. Tissue sections from the frontal lobe were labelled with Hoechst dye (nuclei: red), and imaged by confocal microscopy using a × 60 oil immersion objective. Small capillaries (magenta arrows), the lumen of a larger vessel (magenta star) and brain parenchyma (cyan stars) are indicated. Tile scans (2 × 2) and z-slices were merged to generate a 2D maximum intensity projection. Scale bar, 100 μm. (**c**) Line profile through the brain endothelium (depicted by orange line shown in images from (**b**)). The mean fluorescence intensity of dextran (green) indicates area of high perfusion (that is, in the brain endothelium). (**d**) Bar graph showing the accumulation of top 4 CPPs in the brain parenchyma. Regions outside the areas with high dextran signal (such as those indicated with cyan stars) were selected and the mean fluorescence intensity was quantified (*n*=10). The graph shows s.d. error bars, and statistical analyses were performed using the one-way ANOVA and Dunnett’s multiple comparison test (****P<0.0001).

Emerging evidence suggests that CPPs, a group of peptides with enhanced ability to cross cellular membranes^35^ can also cross the BBB through a variety of different mechanisms^36^,^37^. However, only a small number of CPPs have been investigated for their ability to cross the BBB, and their exact mechanisms of entry remain unclear. We sought to demonstrate the utility of the spheroid model for screening a panel of 16 CPPs labelled with Cy5.5 dye (Table 1), and test its ability to predict *in vivo* BBB penetration. Established brain-penetrating CPPs, such as HIV-1 Tat, penetratin and SynB1 (refs 36, 38, 39, 40, 41) were also labelled with Cy5.5 and included as standards in this screen. We observed a high level of HIV-1 Tat and penetratin influx in the spheroids compared with SynB1 (Fig. 7a), although this might be due to the higher degradation rate of SynB1 (∼10% product remaining after 3 h in working media) compared with HIV-1 Tat and penetratin (50 and 70% product remaining, respectively; Supplementary Fig. 7a). Most of the other CPPs, which had not been previously studied for BBB penetration, exhibited similar or higher influx level than HIV-1 Tat and penetratin, and their relative permeability was ranked as shown in Fig. 7a. We measured the stability of each peptide in BBB media and observed no clear correlation between peptide influx and the stability of each peptide in the working media (Supplementary Fig. 8a), supporting the fact that the observed influx was likely an attribute to the permeability characteristic of each peptide.

The top 5 CPPs with the highest fluorescence level inside the spheroids were DPV 15, HoxA-13, Engrailed-2, Bip(1) and Bip(2) (Fig. 7a). These CPPs remained relatively stable in working media during the course of the experiment (Supplementary Fig. 8a). Time-course analysis of the top 5 CPPs compared to a cell impermeable peptide analogue of the Bim BH3 domain^42^,^43^ revealed Bip(1), Bip(2) and DPV15 to have the highest influx rate at earlier time points (15 min–1 h), followed by HoxA-13 and Engrailed-2 (Supplementary Fig. 8b). At later time points (2–6 h), an equilibrium state was achieved, where all 5 CPPs reached a maximum level of influx (Supplementary Fig. 8b). To test if these CPPs were disrupting the integrity of the spheroid surface barrier, the spheroids were co-incubated with both the CPP and a high molecular weight dextran. As expected, the CPPs displayed a high level of influx into the spheroids after 3 h compared with the control Bim BH3-derived peptide (Supplementary Fig. 8c). Except for spheroids that were co-incubated with Engrailed-2, no significant dextran influx was observed for the other CPPs compared to the ‘no peptide’ control (Supplementary Fig. 8c). Our observation that Engrailed-2 led to a significant increase of dextran influx into the spheroids suggests that Engrailed-2 may compromise the integrity of the BBB, and was thus omitted from further investigations *in vivo*.

The remaining four Cy5.5-labelled CPP candidates, HoxA-13, DPV15, Bip(1) and Bip(2) were administered intravenously into healthy nude mice via the tail vein. After 30 min, fluorescein isothiocyanate (FITC)-dextran was injected intravenously into each mouse so that perfusion could also be easily visualized. Fifteen minutes later (a total of 45 min after CPP administration), the mice were euthanized, their brains flash-frozen and the frontal lobes cryosectioned. Confocal microscopy revealed that all 4 CPPs accumulated within the brain parenchyma beyond areas of high perfusion (that is, blood vessels), though each CPP demonstrated variation in its level of extravasation across the BBB (Fig. 7b,d). Line profiling through capillaries showed a spike in fluorescence intensity for both dextran and CPPs within the vessels (Fig. 7c), indicating that each peptide may have either still been present in the blood circulation at the time of killing, or that they displayed substantial uptake by the endothelium (Fig. 7c). All 4 CPPs could be detected outside of the vessels and in the brain tissues, though slightly elevated peptide signal was observed in areas that were in closer proximity to the vessels (Fig. 7c). Particularly, HoxA-13 displayed the most prominent level of brain accumulation, followed by Bip(2), Bip(1) and then, DPV15 (Fig. 7d). While this trend did not directly correspond with observations from the spheroid model (Fig. 7a), this discrepancy is expected for a few reasons: (1) the pharmacokinetics and biodistribution of each CPP in a living system cannot be accounted for in the *in vitro* spheroid model; (2) the utility of human brain cells in the spheroid model limits direct comparison with the mouse BBB; (3) the inability of the spheroid model to simulate shear stress and blood flow; and (4) CPPs have different serum stability profiles in mice. Indeed, our *ex vivo* serum stability analysis showed that HoxA-13 and Engrailed-2 have the highest stability in mouse serum (74 and 65% of peptide detected after 1 h, respectively), followed by Bip(1), Bip(2) and DPV15 (53, 33 and 10% of peptide detected after 1 h, respectively; Supplementary Fig. 8d). Although our initial screen using the spheroid model revealed DPV15 as the top ‘hit’ with a rapid influx rate (Fig. 6a and Supplementary Fig. 8b), the relatively low BBB penetration observed in mice (Fig. 6b–d) might be a factor of low peptide stability in mouse serum.

## Discussion

Altogether, the BBB spheroid model is able to reproduce BBB properties and functions through the exhibition of high levels of tight/adherens junctions, efflux pumps and transporters that are required to restrict or regulate the influx of foreign molecules. This model can also offer a highly convenient approach to study mechanisms of BBB transport (that is, paracellular delivery, receptor-mediated transcytosis and so on). We have demonstrated the value and versatility of this model as a screening tool using two different detection approaches: confocal fluorescence microscopy and MALDI mass spectrometry imaging. Importantly, our screening experiments demonstrate that the permeability results obtained *in vitro* are recapitulated *in vivo*. The BBB spheroid model is easily scalable to high-throughput capacity due to the simplicity of the approach and low amount of reagents required to establish the spheroids. Furthermore, the screening throughput of this model can be increased even further through the possibility of integration with automated microscopy and robotics-assisted mass spectrometry technologies. The ease of culture, cost-effectiveness and reproducibility of this model offer a very practical and attractive approach for researchers interested in studying BBB drug transport and developing brain-penetrant drugs for the treatment of CNS diseases.

## Methods

### Materials

For peptide synthesis, N^α^-Fmoc protected L-amino acids were obtained through Advanced ChemTech (Louisville, KY). H-Rink Amide-ChemMatrix resin was obtained from PCAS BioMatrix Inc. (St-Jean-sur-Richelieu, Quebec, Canada). 4-pentynoic acid, 2-(1H-Benzotriazol-1-yl)-1,1,3,3-tetramethyluronium hexafluorophosphate (HBTU), 2-(7-Aza-1H-benzotriazole-1-yl)-1,1,3,3-tetramethyluronium hexafluorophosphate (HATU) purchased from Chem Impex (Wood Dale, IL). N,N-Dimethylformamide, dichloromethane and high-performance liquid chromatography (HPLC)-grade acetonitrile were from EMD Millipore (Billerica, MA). Solvents for HPLC-MS were purchased from EMD and Fluka (Darmstadt, Germany). Cy5.5-azide and Cy5-NHS were purchased from Lumiprobe (Hallandale Beach, FL). 5-TAMRA-COOH was purchased from ChemPep Inc (Wellington, FL). Ni-NTA Agarose beads were from Qiagen. All other reagents were purchased from Sigma-Aldrich.

The following antibodies were used in our studies: mouse anti-human GFAP (Sigma-Aldrich; Cat. # G3893), rabbit anti-human CD31 (Abcam; Cat. # ab28364), rabbit anti-human NG2 (Millipore; Cat. # ab5320), rabbit anti-human VE-cadherin (Cell Signaling Technology; Cat. # 2500P), mouse anti-human ZO-1 (Thermo Fisher Scientific; Cat. # 33–9,100), rabbit anti-human β-catenin (Cell Signaling Technology; Cat. # 8,814), rabbit anti-human LRP-1 receptor (Abcam; Cat. # ab92544), mouse anti-human P-gp [4E3] (Abcam; Cat. # ab10333), rabbit anti-claudin 5 (Abcam; Cat. # ab15106), rabbit anti-occludin (Thermo Fisher Scientific; Cat. # 71–1,500) and rabbit anti-von Willebrand factor (Abcam; Cat. # ab6994). For immunofluorescence staining, anti-mouse Alexa-Fluor 488 and anti-rabbit Alexa-Fluor 546 secondary antibodies (Invitrogen) and Hoechst dye (Life Technologies) were used.

The following reagents were used in our experimental studies: LY335979 (Zosuquidar 3HCl; Selleck Chemicals, Cat. # S1481), rhodamine 123 (Enzo Life Sciences, Cat. # ENZ-52307), recombinant human VEGF (PeproTech, Cat. # 100-20), tetramethylrhodamine isothiocyanate (TRITC)-dextran (155 kDa; Sigma-Aldrich; Cat. # T1287), tetramethylrhodamine isothiocyanate (TRITC)-dextran (4,400 Da; Sigma-Aldrich; Cat. # T1037), FITC-dextran (70 kDa; Sigma-Aldrich; Cat. # 46,945), BKM120 (Buparlisib; Selleckchem, Cat. # S2247) and Dabrafenib (GSK2118436; Selleckchem, Cat. # S2807).

For fluorescence imaging, we use either the Zeiss LSM 710 laser scanning confocal microscope or the Nikon Eclipse TE2000-U epi-fluorescence microscope equipped with a QIClick camera. For liquid chromatography–mass spectrometry, all chromatograms and mass spectra were obtained using an Agilent 6,520 ESI-Q-TOF mass spectrometer.

### Cell lines and culture conditions

Primary human astrocytes (Lonza Bioscience) were cultured in Astrocyte Growth Medium (AGM; astrocyte basal medium supplemented with human endothelial growth factor, insulin, ascorbic acid, GA-1,000 (Gentamicin, Amphotericin-B), L-glutamine and 1% fetal bovine serum (FBS); Lonza Bioscience). Human brain microvascular pericytes (HBVP; ScienCell Research Laboratories, Carlsbad, CA) were cultured in Pericyte Medium (ScienCell Research Laboratories) containing 2% FBS, pericyte growth supplement and penicillin-streptomycin. Human cerebral microvascular ECs (hCMEC/D3; Cedarlane, Canada) were maintained in culture using Endothelial Growth Medium (EGM-2; Lonza Bioscience) containing human endothelial growth factor, hydrocortisone, GA-1,000, FBS, VEGF, hFGF-B, R^3^-IGF-1, ascorbic acid and heparin (Lonza Bioscience). Primary HBMEC (ScienCell Research Laboratories) were cultured in endothelial cell medium (ScienCell Research Laboratories) containing 5% FBS, EC growth supplement and penicillin-streptomycin. For experimental use, astrocytes were maintained between passages p2 and 5, pericytes between passages p2 and 10, hCMEC/D3 cells between passage 27 and 32, and HBMEC between passages 2 and 5. All cells were grown in T75 Cell+ vented-cap tissue culture flasks (Sarstedt AG and Co). For spheroid formation in co-culture condition and functional assays, when hCMEC/D3 ECs were used, cells/spheroids were maintained in EGM-2 (Lonza Bioscience) supplemented with 2% human serum (Valley Biomedical; Cat. # HS1021), but with the elimination of VEGF supplementation. Meanwhile, when HBMECs were used, cells/spheroids were maintained in endothelial basal medium (ScienCell Research Laboratories) supplemented with 2% human serum. Each medium will hereafter be referred to as ‘hCMEC/D3 working medium’ and ‘HBMEC working medium’, respectively. Cells were cultured in a humidified incubator at 37 °C with 5% CO_2_, and 95% natural air. All cell lines were regularly tested for mycoplasma contamination.

### Multicellular BBB spheroid co-culture

Sterile 1% agarose (w/v) was prepared by adding 0.5 mg of molecular biology grade agarose (Bio-rad) into 50 ml of PBS in a conical flask, and boiled in a microwave until completely dissolved. The melted agarose solution was transferred into a sterile tissue culture hood, and 50 μl of the solution was dispensed into each well of a 96-well plate while it was still hot using a multi-channel pipette, and allowed to cool/solidify (∼15 min). Primary human astrocytes, HBVP and HBMEC were released by trypsin/EDTA (Thermo Scientific) and resuspended in HBMEC working medium. The concentration of each cell type was determined using a haemocytometer. 1 × 10^3^ of each cell type was seeded onto the agarose gel in each well of the 96-well plate in a 1:1:1 ratio (final volume=100 μl). Cells were placed in a humidified incubator at 37 °C with 5% CO_2_, and 95% natural air for 48–72 h to allow for the assembly of multicellular BBB spheroids. For spheroid formation using the immortalized hCMEC/D3 EC line: hCMEC/D3, astrocytes and HBVP were released by trypsin/EDTA and resuspended in hCMEC/D3 working medium. 1.5 × 10^3^ of each cell type were seeded onto the agarose gel in each well of the 96-well plate in a 1:1:1 ratio, and spheroids were allowed to form as described above.

To study the arrangement of each cell type during the assembly of a spheroid, HBVP and HBMECs were labelled with CellTracker Violet BMQC (Thermo Scientific) and CellTracker Orange CMRA dye (Thermo Scientific), respectively, while astrocytes were labelled with VivoTrack 680 near-infrared dye (PerkinElmer Inc.). To study spheroids made with the immortalized hCMEC/D3 ECs, hCMEC/D3 were labelled with CellTracker Green CMFDA dye (Thermo Scientific). Briefly, cells were mixed with each dye (1:1,000 dilution for CellTracker; 0.83 g ml^−1^ for VivoTrack) in PBS for 15 min at 37 °C with 5% CO_2_. The cells were then washed 3 times with HBMEC working medium, counted using a haemocytometer and co-cultured on agarose to form the BBB spheroids as described above. After 48 h, spheroids were fixed in 3.7% formaldehyde for 30 min, washed 3 times with PBS, transferred into a Nunc Lab-Tek II thin-glass 8-well chambered coverglass (Thermo Scientific) and imaged under a Zeiss LSM710 confocal microscope. Confocal z-stack images (8 μm slices) were captured through each spheroid (up to 104 μm-deep) using a × 20 objective.

### Dextran permeability assay

Multicellular BBB spheroids were formed through co-culturing astrocytes, HBVP and either HBMEC or hCMEC/D3 as described earlier for 48 h. Spheroids were incubated with increasing concentration of VEGF-A (between 5 and 100 ng ml^−1^) and 10 μg ml^−1^ of TRITC-Dextran (155 kDa; Sigma-Aldrich) or FITC-Dextran (70 kDa; Sigma-Aldrich) in the appropriate working medium for 24 h at 37 °C with 5% CO_2_. Spheroids were washed 3 times with PBS (5 min each), transferred into a Nunc Lab-Tek II thin-glass 8-well chambered coverglass (Thermo Scientific) and imaged under a confocal microscope as described earlier. Quantification of spheroid permeability to fluorescent dextran was performed using ImageJ software (http://imagej.net/Fiji). The mean fluorescence intensity of the core of each spheroid at 88 μm depth was quantified and plotted using GraphPad Prism (version 7.0 software).

### Immunofluorescence staining of BBB spheroids

Multicellular BBB spheroids were established for 48 h as described earlier. Spheroids were collected and pooled into a 0.2 ml Eppendorf tube (Corning Inc.). The spheroids were washed once in PBS and then fixed in 3.7% formaldehyde for 10 min at room temperature (RT). Spheroids were washed twice in PBS and then permeabilized with PBS containing 0.1% Tween-20 (v/v) for 30 min. Blocking was performed in 10% normal donkey serum (NDS) diluted in PBS containing 0.025% Tween-20 (v/v) for 1 h at RT under constant rotation. Then, we added the following primary anti-human antibodies: ZO-1 (1:100 dilution), P-gp (1:100 dilution), LRP-1 receptor (1:100 dilution) or β-catenin (1:500 dilution), and incubated the spheroids overnight at 4 °C under constant rotation. Spheroids were washed 3 times with PBS containing 0.025% Tween-20, and then incubated appropriately with either anti-mouse Alexa Fluor 488 or anti-rabbit Alexa Fluor 546 (Invitrogen; 1:1,000 dilution) and Hoechst dye (1:1,000 dilution) in 10% NDS diluted in PBS containing 0.025% Tween-20 (v/v) for 1 h at room temperature under constant rotation. Spheroids were then washed 3 times with PBS containing 0.025% Tween-20 and transferred into a Nunc Lab-Tek II thin-glass 8-well chambered coverglass (Thermo Scientific; Cat. # T-2825-8). Spheroids were imaged using a confocal microscope under a × 20 or × 40 oil immersion objective. Z-slices captured through each spheroid (up to 100 μm-deep) were merged to generate a 2D maximum intensity projection or reconstructed to form a 3D image using the ZEN blue imaging software (version 2012, Zeiss, Jena, Germany).

### Transmission electron microscopy of BBB spheroids

Multicellular BBB spheroids were established for 48 h as described earlier. Spheroids were collected and pooled into an Eppendorf tube. The spheroids were washed once in PBS, and then fixed overnight in 2.5% glutaraldehyde, 1.25% paraformaldehyde and 0.03% picric acid in 0.1 M sodium cacodylate buffer (pH 7.4) at 4 °C. Spheroids were then, washed in 0.1 M cacodylate buffer and post-fixed with 1% osmium tetroxide (OsO4)/1.5% potassium ferrocyanide (KFeCN6) for 1 h, washed 2 × in water, 1 × in maleate buffer and incubated in 1% uranyl acetate in maleate buffer for 1 h, followed by 2 washes in water and subsequent dehydration in the following grades of alcohol (10 min each; 50%, 70%, 90% and 2 × 10 min 100%). The samples were then placed in propyleneoxide for 1 h and infiltrated overnight in a 1:1 mixture of propyleneoxide and TAAB Epon (Marivac Canada Inc. St Laurent, Canada). The following day, the samples were embedded in TAAB Epon and polymerized at 60 °C for 48 h. Ultrathin sections (∼80 nm) were cut on a Reichert Ultracut-S microtome, picked up on to copper grids stained with lead citrate and examined using a JEOL 1200EX transmission electron microscope. Images were recorded with an AMT 2 k CCD camera.

### P-glycoprotein function on BBB spheroids

Multicellular BBB spheroids (established with astrocytes, HBVP and HBMEC) were established for 48 h as described earlier, and pooled into a 1.5 ml Lobind microcentrifuge tube (Eppendorf, Hauppauge, NY) in 1.0 ml HBMEC working media. The P-gp inhibitor, LY335979 was added at the following final concentrations: 0.5, 0.05 and 0.005 μM. Rhodamine 123 (Rh123) dye, a substrate of P-gp was added to each tube at 0.5 μg ml^−1^ and incubated for 3 h at 37 °C. The spheroids were then washed 5 times with PBS (5 min each) and fixed with 3.7% formaldehyde for 10 min. Spheroids were transferred into a Nunc Lab-Tek II thin-glass 8-well chambered coverglass (Thermo Scientific), and imaged under a confocal microscope. Confocal z-stack images (8 μm slices) were captured through each spheroid (104 μm-deep) using a × 20 objective. Quantification of spheroid permeability to Rh123 was performed using ImageJ software. The mean fluorescence intensity of the core of each spheroid at 104 μm depth was quantified and plotted using GraphPad Prism.

### Synthesis of angiopep-2 and scrambled angiopep-2

(Note: Scrambled angiopep-2 will henceforth be referred to as ‘scramble’). The peptides were synthesized using an automated flow-based synthesizer using Fmoc chemistry and H-Rink Amide-ChemMatrix resin as previously reported^44^. For all TAMRA-containing peptides, 5-TAMRA was coupled to the N-terminus on resin using HATU. For Cy5-labelled peptides, Cy5-NHS was coupled to the N-terminus on resin. For Cy5.5-labelled peptides, the N-terminus was capped with 4-pentynoic acid and Cy5.5-azide was conjugated using copper-catalyzed ‘click’ chemistry (see below). The peptides were then cleaved from the resin, and labelled peptide was separated from unlabelled using reverse phase HPLC on a Zorbax C_3_ column (9.4 × 250 mm, 5 μm; Agilent). HPLC chromatograms and mass spectra of all proteins and peptides used in this study are depicted in Supplementary Note 1.

### Transcytosis of angiopep-2 into BBB spheroids

Multicellular BBB spheroids (composed of astrocytes, HBVP and HBMEC (or hCMEC/D3)) were established for 48–72 h as described earlier, and pooled into a 1.5 ml Lobind microcentrifuge tube (Eppendorf) in 1.0 ml HBMEC (or hCMEC/D3) working media. Angiopep-2 (or scramble) peptide labelled with a Cy5 was added into each tube at a final concentration of 5 and 10 μM, and incubated for 3 h at 37 °C on a rotator. Spheroids were washed with PBS, fixed with 3.7% formaldehyde and imaged using a confocal microscope as described in earlier sections. The mean fluorescence intensity inside each spheroid at 88 μm depth was quantified using ImageJ software and plotted using GraphPad Prism. The same methodology was applied for angiopep-2/scramble peptide labelled with a TAMRA (tetramethylrhodamine) dye. To inhibit transcytosis, spheroids were incubated with each peptide (5 μM final concentration) in cold working medium (on ice) for 3 h. For cryo-sectioning, spheroids were embedded in optimum cutting temperature compound (Tissue-Tek, Sakura Finetek, Torrance, CA), frozen on dry ice and sliced into 10 μm sections. Spheroid sections were imaged under a Nikon epi-fluorescence inverted microscope.

To examine if angiopep-2 affects the overall permeability of the spheroid surface barrier, spheroids were co-incubated with 10 μM of TAMRA-angiopep (or TAMRA-scramble) and FITC-dextran (70 kDa; 10 μg ml^−1^) as described earlier for 3 h at 37 °C on a rotator. Spheroids were then washed 3 times with PBS, fixed with 3.7% formaldehyde for 10 min and imaged using confocal microscopy as described earlier. The mean TAMRA and FITC fluorescence intensity of the core of each spheroid at 88 μm depth was quantified using ImageJ software and plotted using GraphPad Prism.

### *Ex vivo* imaging of angiopep-2 in mouse brain

Cy5.5-angiopep-2 or Cy5.5-scramble were diluted to a final concentration of 1 mg ml^−1^ in 0.9% NaCl irrigation solution (Hospira, Lake Forest, IL). 100 μl of each peptide (total of 100 μg) was administered intravenously via tail vein injection. After 24 h, mice were killed (by CO_2_ asphyxiation), and immediately perfused with PBS followed by 10% formalin solution (Sigma-Aldrich). Brains were excised, and a coronal cut through the cerebral cortex was made with a sharp scalpel. The brains were washed several times with PBS containing 0.05% Tween-20, and incubated with DyLight 488 lectin (Vector Laboratories, Burlingame, CA) and Hoechst dye (Life Technologies; 1:1,000 dilution) for 1 h in PBS containing 0.02% Tween-20. This was followed by 3 washes (15 min each) with PBS containing 0.05% Tween-20. The brain slices were transferred into a Nunc Lab-Tek II thin-glass 8-well chambered coverglass (Thermo Scientific), with the coronal plane of the cerebral cortex facing the bottom of the chamber. The brain tissue was imaged under a confocal microscope using a × 40 oil immersion objective. Z-slices captured through each spheroid (up to 100 μm-deep) were merged to generate a 2D maximum intensity projection using the ZEN blue imaging software (version 2012). Animal experiments described here are listed in the Brigham and Women’s Hospital Animal Experimentation Protocol 2016N000300 *Translational Neurooncology Core* (BWH IACUC approved 2016).

### Evaluation of BKM120 or dabrafenib transport using MALDI-MSI

BBB spheroids were established (using astrocytes, HBVP and hCMEC/D3 cells) for 48 h as described earlier. Approximately 150 spheroids per group were pooled into a 1.5 ml microcentrifuge tube in 1 ml of hCMEC/D3 working media. BKM120 or Dabrafenib was added into each tube at a final concentration of 10 μM and incubated for 24 h at 37 °C under constant rotation. Spheroids were washed 5 × with wash buffer (150 mM ammonium acetate in HPLC grade water, pH 7), and then transferred into an UltraFlux 0.2 ml PCR tube (BioExpress) in 200 μl of wash buffer. The spheroids were then, pooled at the bottom of the tube, and snap-frozen in a dry ice/ethanol bath. The top of the frozen tube was mounted onto a cryostat holder, and the bottom of the tube containing the spheroids was sectioned into 12 μm slices using a Microm HM550 cryostat, with the chamber and the specimen holder chilled at −26 °C. Sectioned tissues embedded in the frozen wash buffer were thaw-mounted onto indium tin oxide (ITO) coated microscopic slides (Bruker Daltonics) for MALDI-MSI, and regular positively charged glass slides for haematoxylin and eosin (H/E) staining. The tissues were allowed to dry for at least 15 min in a dessicator. 2,5-dihydroxybenzoic acid matrix (2,5-DHB, 160 mg ml^−1^ solution in methanol/0.1% trifluoroacetic acid (TFA) 70:30 v/v) was deposited using a TM-sprayer (HTX imaging) under the following conditions: flow rate, 180 μl min^−1^; spray nozzle velocity, 1,200 mm min^−1^; spray nozzle temperature, 75 °C; nitrogen gas pressure, 10 psi; track spacing, 2 mm; and number of passes, 2.

For MALDI-MS imaging, mass spectra were acquired using a 9.4T SolariX XR Fourier transform ion cyclotron resonance mass spectrometer (Bruker Daltonics). MALDI-MSI experiments were acquired with a pixel step size for the surface raster set to 30 μm in FlexImaging 4.1 software (Bruker Daltonics). The analyses were performed in positive ion mode by continuous accumulation of selected ions in a mass range comprised between *m*/*z* 405 and 525 and a laser intensity set to 25%. Each mass spectrum is the sum of 250 laser shots at a laser frequency of 1,000 Hz. The MALDI images were displayed using FlexImaging 4.1 after total ion current normalization. The distribution of the drugs is visualized following the signal of BKM120 at *m/z* 411.1751±0.001 and dabrafenib at *m/z* 520.1083±0.001.

### Transwell co-culture model

Both the apical and basal sides of 24-well transwell insert (0.4 μm pore size; Greiner Bio-One, Cat. # 662,641) were coated with rat-tail collagen (5 μg ml^−1^ in PBS; Olaf Pharmaceuticals, Worcester, MA) overnight at 4 °C. Each transwell was inverted, and 5 × 10^4^ HBVP cells (in 50 μl pericyte medium) were seeded on the basal side of the insert. The inserts were encased in an inverted plate, and allowed to adhere in a humidified incubator for 2 h at 37 °C with 5% CO_2_. Inserts were then removed and placed within a 24-well plate (upright) containing 300 μl pericyte medium, ensuring that the HBVP cells on the basal side of the insert was properly submerged in the medium. 5.0 × 10^7^ hCMEC/D3 cells were seeded on the apical side of the insert (in 200 μl volume) in EGM-2. In a separate 24-well plate, 5.0 × 10^4^ astrocytes were seeded in AGM. All cells were allowed to adhere/grow overnight at 37 °C with 5% CO_2_. On the next day, cells on both the apical and basal sides of the transwell, as well as the astrocytes in the 24-well plate were washed twice with hCMEC/D3 working media. The inserts (containing hCMEC/D3 on the apical side, and HBVP on the basal side) were then transferred into the 24-well plate containing the astrocytes cells using sterile forceps. The final volume of hCMEC/D3 working medium was 500 μl in the basal compartment and 200 μl on the apical side of the insert. The transwell co-culture system was placed in a humidified incubator at 37 °C with 5% CO_2_ for an additional 60 h. The media was replaced with fresh hCMEC/D3 working medium every 2 days. To obtain transepithelial electrical resistance (TEER) measurements, media on both the apical and basal side of the insert were replaced with fresh hCMEC/D3 working medium (300 μl on the apical side; 1 ml on the basal side), and measurements were obtained using the EVOM-2 epithelial voltohmeter instrument (World Precision Instruments) at *t*=60 and 84 h. TEER measurements of inserts without cells were subtracted from TEER values obtained from inserts containing cells (measured in ohms (Ω)). The measurements were multiplied by 0.336 cm^2^ (surface area of the 24-well transwell insert) to generate a TEER value in Ω·cm^2^.

### Transwell permeability study

After 84 h of co-culture in the transwell, TAMRA- angiopep-2 or TAMRA-scramble were added into the apical side of the insert at a final concentration of 10 μM. Image tiling/stitching was used to capture across the entire centre of each transwell, and images were acquired every 20 min for 40 h. To assess the apical-to-basolateral permeability, the mean fluorescence intensity of the basal side of the transwell was quantified over time using ImageJ software and plotted using GraphPad Prism.

### Immunofluorescence staining of ECs co-cultured on transwell

All cells on the apical and basal side of the transwell were washed with PBS and fixed with 3.7% formaldehyde for 30 min. The basal side of the insert’s membrane was wiped with a cotton swab to remove all HBVP cells. Both sides of the transwell were washed twice in PBS and then, permeabilized with PBS containing 0.1% Tween-20 (v/v) for 30 min. Then, hCMEC/D3 ECs on the apical side of the insert was blocked with 10% NDS diluted in PBS containing 0.025% Tween-20 (v/v) for 1 h at RT. The following primary anti-human antibodies: ZO-1 (1:100 dilution), P-gp (1:100 dilution) or β-catenin (1:500 dilution) was added into the blocking solution and incubated overnight at 4 °C. The next day, hCMEC/D3 cells were washed 3 times with PBS containing 0.025% Tween-20, and incubated appropriately with either anti-mouse Alexa Fluor 488 or anti-rabbit Alexa Fluor 546 (Invitrogen; 1:1,000 dilution) and Hoechst dye (1:1,000 dilution) in 10% NDS diluted in PBS containing 0.025% Tween-20 (v/v) for 1 h at RT. Cells were then washed 3 times with PBS containing 0.025% Tween-20 (care was taken to not puncture the membrane). The insert was placed on a 24 × 60 mm glass coverglass (Fisherfinest, Cat. # 12-548-5P) for support so that it could be imaged under our confocal microscope using a × 20 objective. Z-slices captured through the hCMEC/D3 cells on the insert were merged to generate a 2D maximum intensity projection using ZEN blue imaging software (version 2012).

### Synthesis of Cy5.5-labelled CPPs

The CPPs were synthesized using an automated flow-based synthesizer using Fmoc chemistry as previously reported^44^,^45^. The N-terminus was capped with an alkyne, 4-pentynoic acid. After cleavage from the resin, the peptides were purified by reverse phase HPLC using an Agilent Zorbax C_3_ or C_18_ column (9.4 × 250 mm, 5 μm) and pure fractions were combined and lyophilized. Cy5.5-azide was attached using modification of established protocols^46^. Briefly, the CPP (2.38 μmol) was mixed with 1.2 equiv. (2.86 μmol) Cy5.5-azide in a 50:50 water:*tert-*butanol mixture containing 5 mM CuSO_4_, 100 mM Ascorbic acid, 50 mM Tris (pH 8.0), 1 mM tris(2-carboxyethyl)phosphine and 0.1 mM tris(benzyltriazolylmethyl)amine. After 2 h, the reaction mixture was diluted with water/acetonitrile and purified by HPLC to provide the Cy5.5-CPP conjugates. HPLC chromatograms and mass spectra of all proteins and peptides used in this study are depicted in Supplementary Note 1.

### Screening for BBB-penetrating CPPs

Multicellular BBB spheroids (using astrocytes, HBVP and hCMEC/D3 cells) were established for 48 h as described earlier, and pooled into a 0.5 ml microcentrifuge tube (Eppendorf) in 500 μl of hCMEC/D3 working media. Each CPP was added into each tube at a final concentration of 5 μM and incubated for 3 h at 37 °C under constant rotation. Spheroids were washed with PBS, fixed with 3.7% formaldehyde and imaged using a confocal microscope as described in earlier sections. The mean fluorescence intensity inside each spheroid (at 88 μm depth from the surface) was quantified using ImageJ software and plotted using GraphPad Prism.

### Evaluation of CPP brain entry

The top brain-penetrant CPPs identified from the above screen (Bip(1), Bip(2), DPV15 and HoxA-13) were dissolved in DMSO at 50 mM stock concentration. Each peptide was then diluted to a final concentration of 500 μM in a solution of 50% polyethylene glycol (PEG)-300 (Kollisolv; Sigma-Aldrich) in 0.9% sodium chloride irrigation solution (Hospira). 100 μl of each peptide was administered intravenously via tail vein injection into healthy 7-week-old female nude mice. After 30 min, 100 μl of (TRITC)-dextran (155 kDa; 50 mg ml^−1^) was injected intravenously via the tail vein. Fifteen minutes later, mice were killed (by CO_2_ asphyxiation and cervical dislocation), and their brains excised and immediately flash frozen in a dry ice/ethanol bath. Whole brain cryosections (20 μm) were obtained using the Microm HM550 cryostat (Thermo Scientific). The tissues were then fixed in 3.7% formaldehyde for 10 min, stained with Hoechst dye (1:1,000 dilution in PBS), mounted with a coverslip in VECTASHIELD Antifade Mounting Media (Vector Laboratories) and imaged under a confocal microscope using a × 63 oil immersion objective. Tile scans (2 × 2) and z-slices were merged to generate a 2D maximum intensity projection using ZEN black imaging software (version 2012).

### Serum stability analyses

*Cy5.5-angiopep and Cy5.5-scramble*. Angiopep and scramble peptides (50 μM) were incubated in hCMEC/D3 working media (containing 2% human serum) at 37 °C. After 0, 1, 2, 4, 8 and 24 h, 10 μl of the crude reaction was transferred to a microcentrifuge tube, flash frozen and lyophilized to dryness. The material was resuspended in 30 μl of 50:50 water:acetonitrile containing 0.1% TFA. The sample was injected onto the Agilent 6520 Q-TOF MS. An extracted ion current (EIC) for the most abundant charge state *m*/*z* was analysed using the MassHunter software. The EIC peak was integrated and per cent peptide intact was determined by (EICt_1_/EICt_0_) × 100 in which EICt_1_ is the peak integration at a given time point and EICt_0_ is the peak integration at time *t*=0. The process was repeated (*n*=3) for each peptide.

*Cy5.5-CPPs*. All 19 Cy5.5-CPPs (5 μM) were incubated in hCMEC/D3 working media at 37 °C. After 0 and 3 h, 5 μl of the crude reaction was transferred to a microcentrifuge tube, flash frozen and lyophilized to dryness. The material was resuspended in 30 ml of 50:50 water:acetonitrile containing 0.1% TFA. The sample was injected onto the Agilent 6520 Q-TOF MS. An EIC for the most abundant charge state *m/z* was analysed using the MassHunter software. The EIC peak was integrated and percentage peptide intact was determined by (EICt_3_/EICt_0_) × 100, in which EICt_3_ is the peak integration at 3 h and EICt_0_ is the peak integration at time *t*=0. Before *in vivo* studies, a similar protocol was used to assess the stability of the top Cy5.5-Cpps in mouse serum. Briefly, each peptide (50 μM) was incubated in PBS containing 10% mouse serum at 37 °C. After 0, 1, 2, 4 and 8 h, 10 ml of the crude reaction was transferred to a microcentrifuge tube, flash frozen and lyophilized to dryness. The samples were redissolved and analysed by mass spectometry using identical conditions to those used for testing the whole peptide library in working media.

### Data availability

All data generated or analysed during this study are included in this published article (and its Supplementary Information Files). The data sets are available from the corresponding authors on reasonable request.

## Additional information

**How to cite this article:** Cho, C.-F. *et al*. Blood-brain-barrier spheroids as an *in vitro* screening platform for brain-penetrating agents. *Nat. Commun.* **8,** 15623 doi: 10.1038/ncomms15623 (2017).

**Publisher’s note:** Springer Nature remains neutral with regard to jurisdictional claims in published maps and institutional affiliations.

## Supplementary Material

Supplementary Information Supplementary Figures, Supplementary Methods, Supplementary Note and Supplementary References

## Figures and Tables

**Table 1:** Name, sequences and properties of CPPs used in our screen to investigate their BBB-penetrating ability.

**Name**	**Sequence**	**CPP chemical class**	**CPP origin**	**Number of R,H,K**	**Number of residues**	**% cationic residues**	**MW (Da)**
SynB1	RGGRLSYSRRRFSTSTGR	Amphipathic	From antimicrobial peptides	6	18	33	2842.1
L-2	HARIKPTFRRLKWKYKGKFW	Amphipathic	From antimicrobial peptides	9	20	45	3388.5
PreS2-TLM	PLSSIFSRIGDP	Amphipathic	From viral proteins	1	12	8	2030.7
Transportan 10	AGYLLGKINLKALAALAKKIL	Amphipathic (cationic)	Designed	4	21	19	2924.4
SAP	VRLPPPVRLPPPVRLPPP	Amphipathic (proline rich)	Designed	3	18	17	2739.3
SAP(E)	VELPPPVELPPPVELPPP	Amphipathic (proline rich)	Designed	0	18	0	2658.1
SVM3	KGTYKKKLMRIPLKGT	Amphipathic (cationic)	From computational prediction	6	16	38	2604.1
SVM4	LYKKGPAKKGRPPLRGWFH	Amphipathic (cationic)	From computational prediction	7	19	37	2978.3
Bip(1)	VPALR	Hydrophobic	From natural protein (hydrophobic)	1	5	20	1297.4
Bip(2)	VSALK	Hydrophobic	From natural protein (hydrophobic)	1	5	20	1259.3
(PPR)3	PPRPPRPPR	Amphipathic (proline rich)	Designed	3	9	33	1811.6
(PPR)5	PPRPPRPPRPPRPPR	Amphipathic (proline rich)	Designed	5	15	33	2512.0
DPV1047	VKRGLKLRHVRPRVTRMDV	Cationic	From heparin binding protein	8	19	42	3058.4
DPV15	LRRERQSRLRRERQSR	Cationic	From heparin binding protein	8	16	50	2924.3
HIV-1 Tat	RKKRRQRRR	Cationic	From RNA binding protein	8	9	89	2081.9
Penetratin	RQIKIWFQNRRMKWKK	Cationic	From DNA binding protein	7	16	44	2988.3
Engrailed-2	SQIKIWFQNKRAKIKK	Catioinic	From DNA binding protein	6	16	38	2758.2
HoxA-13	RQVTIWFQNRRVKEKK	Cationic	From DNA binding protein	6	16	38	2858.2
Polyarginine	RRRRRRRR	Cationic	Designed	8	8	100	2009.8
