# Supplementary material for: Blood-brain-barrier spheroids as an in vitro screening platform for brain-penetrating agents
Source: Nat Commun. 2017 Jun 6;8:15623. doi: 10.1038/ncomms15623 (PMC5467173; doi:10.1038/ncomms15623)
Supplement: Supplementary Information — Supplementary Figures, Supplementary Methods, Supplementary Note and Supplementary References [file ncomms15623-s1.pdf]

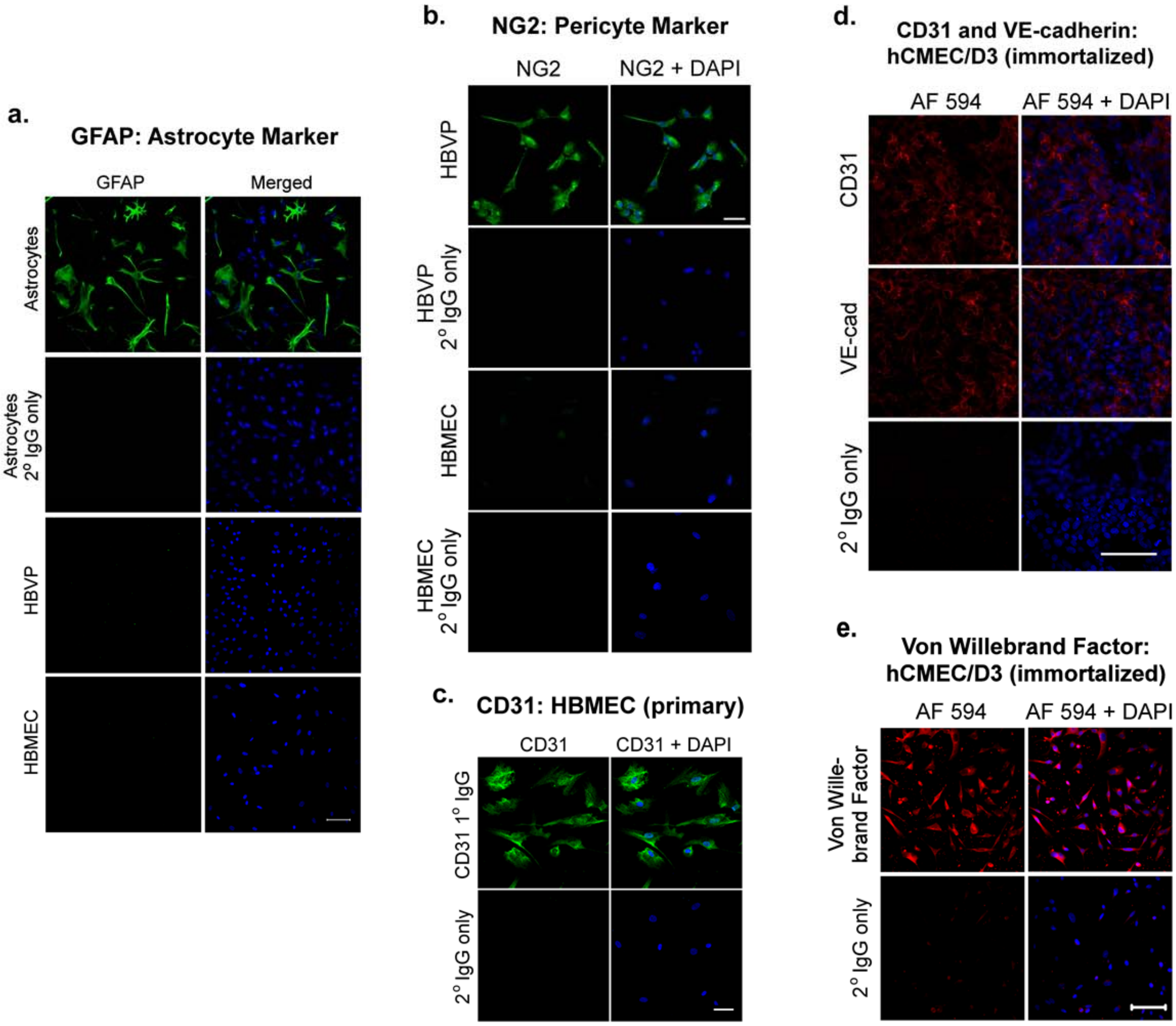

Supplementary Figure 1. Characterization of each cell type used in establishing the multicellular BBB spheroids. (a) Fluorescence images showing the expression of GFAP (an astrocyte marker) in normal human astrocytes but not in primary human brain vascular pericytes (HBVP) and brain microvascular endothelial cells (HBMEC). Scale bar: 100 microns. (b) Fluorescence images showing the expression of NG2 (a pericyte marker) in pericyte cells but not in HBMECs. Scale bar: 50 microns. (c) Fluorescence images showing the expression of CD31 (an endothelial cell marker) in primary endothelial cells (HBMECs). Scale bar: 50 microns. (d) Fluorescence images showing the expression of CD31 and VE-cadherin (endothelial cell markers) in immortalized human cerebral microvascular endothelial cells hCMEC/D3 endothelial cells. Scale bar: 100 microns. (e) Fluorescence images showing the expression of Von Willebrand Factor in immortalized human cerebral microvascular endothelial cells hCMEC/D3 endothelial cells. Scale bar: 100 microns. Cells incubated with secondary (2°) IgG only (no primary IgG) were used as negative controls. In all cases, cell nuclei were labeled with Hoechst dye (blue).

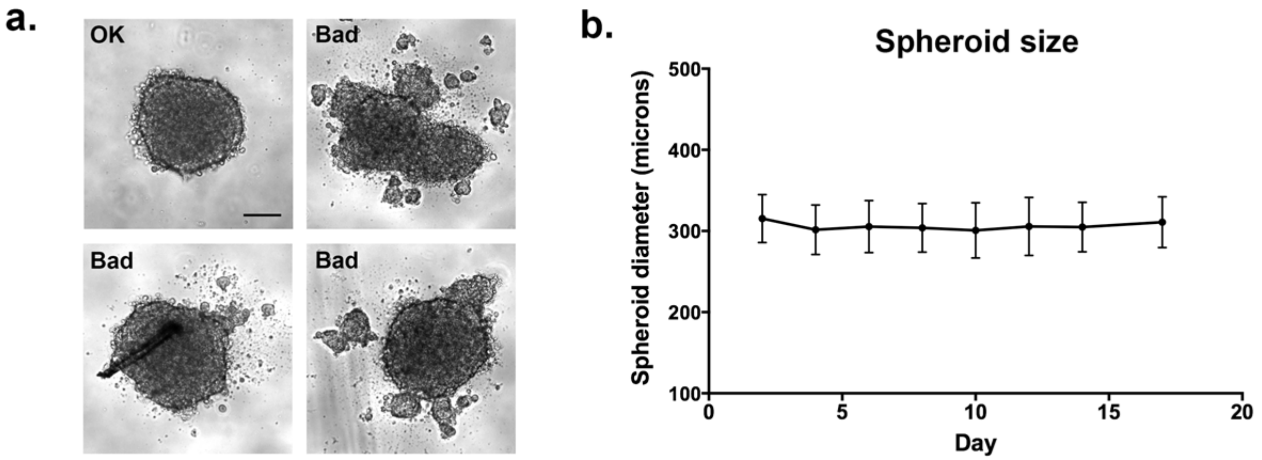

Supplementary Figure 2. Reproducibility and viability of BBB spheroids. (a) Brightfield images showing representative images of "acceptable" vs. "failed" BBB spheroids cultured on 1% agarose for 48 hours within a 96-well plate. Scale bar: 100 microns. (b) Long-term analysis of BBB spheroid viability. The diameter of each spheroid was quantified using ImageJ over 17 days ($n_{spheroid}$ = 11, $n_{experiment}$ = 2). Working media was changed every 2 days.

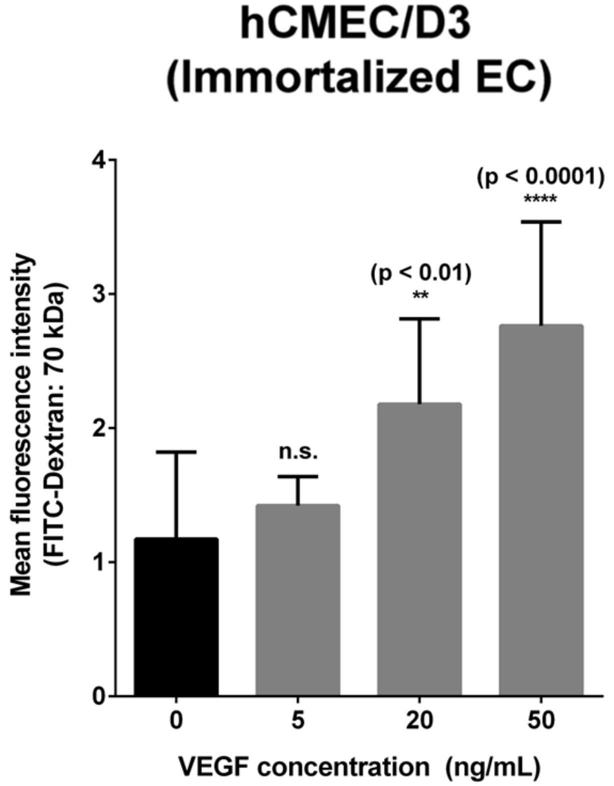

Supplementary Figure 3. Surface permeability of BBB spheroid to 70 kDa dextran. Permeability assay showing that VEGF-A (at 5, 20 and 50 ng/mL) increased spheroid permeability to FITC-Dextran (70kDa) using spheroids established with immortalized hCMEC/D3 endothelial cells. Graph shows mean FITC intensity quantified at 88 μm depth from the surface of the spheroid with standard deviation (SD) error bars (** $p < 0.01$; **** $p < 0.0001$). Statistical analyses were performed using the one-way ANOVA and Dunnett's multiple comparsion test ($n_{spheroid} = 8$).

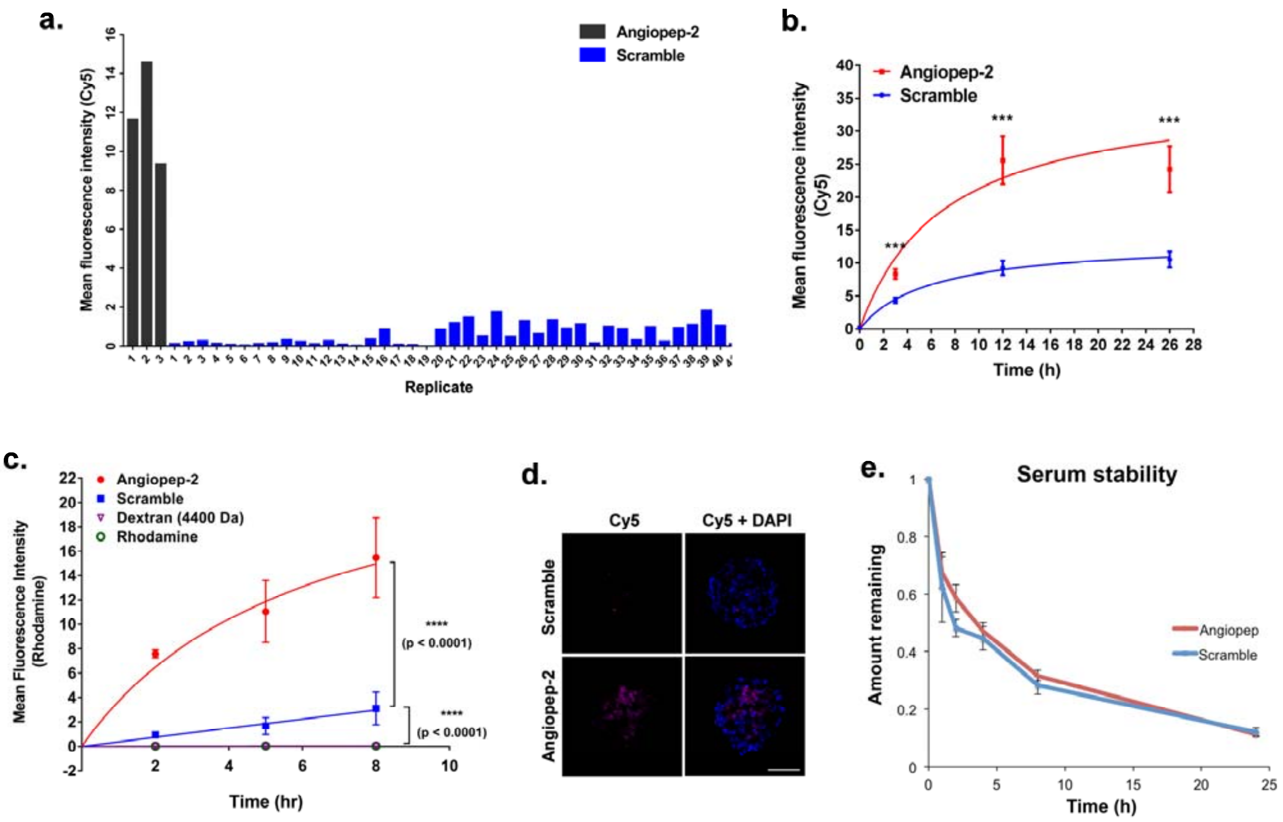

Supplementary Figure 4. Analysis of the transport of angiopep-2 into the BBB spheroid. (a) Bar graph depicting the reproducibility of the BBB spheroid in regulating Cy5-angiopep (and Cy5-scramble) transport. Three spheroids were incubated with Cy5-angiopep, while 41 spheroids were incubated with Cy5-scramble (at 5 µM concentration) for 3 hours at 37°C. Spheroids were imaged using confocal microscopy, and quantified at 88 µm depth from the surface of the spheroid using ImageJ. (b) Time course analysis of the transport of Cy5-angiopep (and Cy5-scramble) into the BBB spheroid over 26 hours (at 10 µM concentration) ($n_{spheroid} = 9$) (*** $p < 0.0001$). (c) Time course analysis of the transport of TAMRA-angiopep, TAMRA-scramble, TAMRA (rhodamine) and rhodamine-dextran (dextran 4400 Da) into the BBB spheroid over 8 hours (at 1 µM concentration) ($n_{spheroid} = 5\text{-}6$). The graphs display mean rhodamine fluorescence intensity quantified at 88 µm depth from the surface of each spheroid with SD error bars. Statistical analysis was performed using the multiple t-test (*** $p < 0.0001$). (d) Fluorescence images of a spheroid cryosection showing the accumulation of angiopep-2 (shown in magenta) in the core of the spheroid. The nuclei of the spheroids (stained with DAPI) is shown in blue. Scale bar: 100 microns. (e) Serum stability of Cy5.5-angiopep and Cy5.5-scramble (50 µM) at 37 °C in hCMEC/D3 working media containing 2% human serum. At each time point, an aliquot was subjected to LC-MS analysis to quantify the amount of peptide that remained relative to t=0.

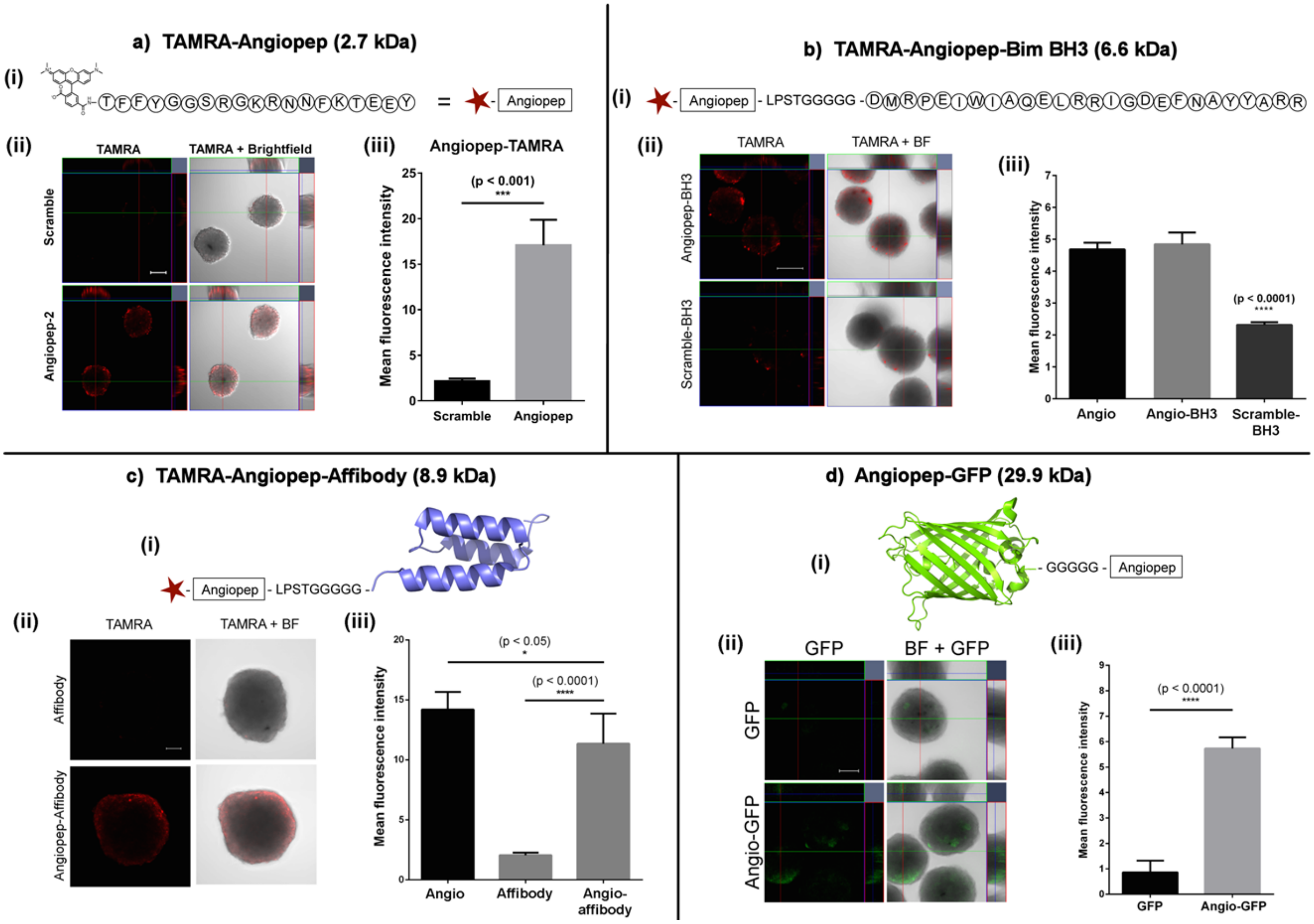

Supplementary Figure 5. Transport of angiopep-conjugated cargoes of various sizes into the BBB spheroid. (a-i) Structure of TAMRA-conjugated angiopep-2. (a-ii) Fluorescence images showing the transport of TAMRA-angiopep (red) (compared to TAMRA-scramble) in spheroids established with primary HBMECs. Scale bar: 100 microns. (a-iii) Bar graph displaying the mean fluorescence intensity quantified from images (a-ii) with SD error bars ($n_{spheroid} = 6$). (b-i) Structure of TAMRA-angiopep conjugated to an analogue of the Bim BH3 domain. (b-ii) Fluorescence images showing the transport of TAMRA-angiopep-Bim BH3 (red) (compared to TAMRA-scramble-Bim BH3 and TAMRA-angiopep) in spheroids established with primary HBMECs (final concentration: 5 µM). Scale bar: 200 microns. (b-iii) Bar graph displaying the mean fluorescence intensity quantified from images (b-ii) with standard error of mean (SEM) error bars ($n_{spheroid} = 17$). Statistical significance was compared with TAMRA-angiopep-Bim BH3. (c-i) Structure of TAMRA-angiopep conjugated to a HER-2 targeted affibody. (c-ii) Fluorescence images showing the transport of TAMRA-angiopep-affibody (red) (compared to TAMRA-affibody) in spheroids established with primary HBMECs (final concentration: 2.5 µM). Scale bar: 50 microns. (c-iii) Bar graph displaying the mean fluorescence intensity quantified from images (b-ii) with standard deviation (SD) error bars ($n_{spheroid} = 6$). (d-i) Structure of angiopep conjugated to green fluorescent protein (GFP). (d-ii) Fluorescence images showing the transport of angiopep-GFP (green) (compared to GFP protein alone) in spheroids established with primary HBMECs (final concentration: 2.5 µM). Scale bar: 100 microns. (b-iii) Bar graph displaying the mean fluorescence intensity quantified from images (b-ii) with standard error of mean (SEM) error bars ($n_{spheroid} = 18$). All spheroids were incubated at 37°C for 3 hrs, and imaged using confocal microscopy, and quantified at 88 µm depth from the surface of the spheroid. Statistical analyses were performed using either an unpaired student's T-test or the one-way ANOVA and Tukey's multiple comparison test.

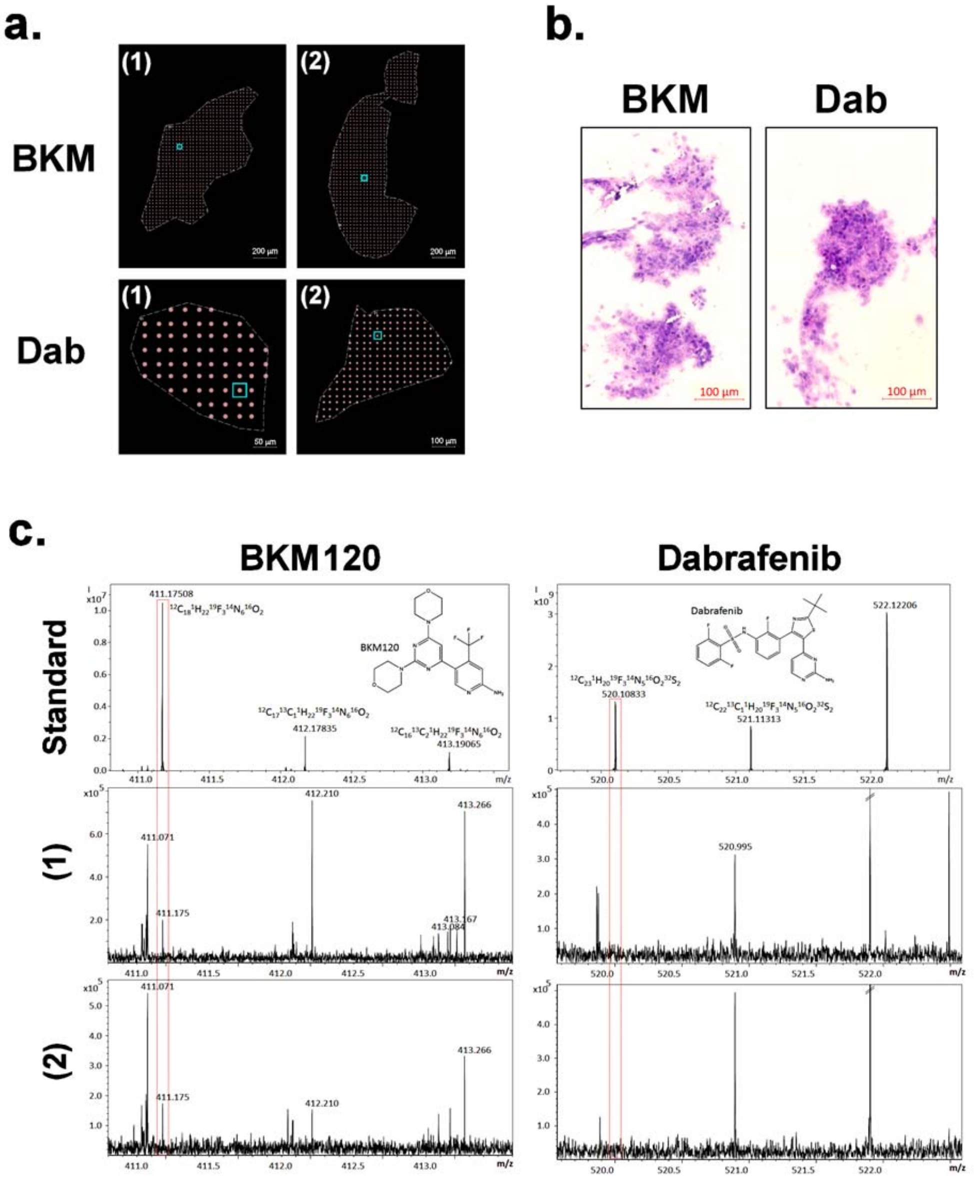

Supplementary Figure 6. Transport of BKM120 (a BBB-penetrant drug) and dabrafenib (a non-penetrant drug) into BBB spheroids. (a) Images displaying the tissue area corresponding to the BBB spheroid sections (outlined with white dashed lines) and the distribution of the pixels analyzed by MALDI mass spectrometry (pink dots) ($n_{spheroid}$ = 150, n_{tissue} = 2). Pixels are spaced at a spatial resolution of 30 µm. Scale bars are indicated within each image. (b) H&E stained images of serial section from each group, confirming the presence of BBB spheroid tissue. Scale bar: 100 µm. (c) Top panels display mass spectra from MALDI MS analysis of BKM120 and dabrafenib (Dab) standard solutions. Positive single-charged ions were detected at monoisotopic *m/z* values of 411.1751 for BKM120, and 520.1083 for dabrafenib. The middle and bottom panels display spectra that correspond to each pixel indicated by a cyan box in (a). The left panels indicate that BKM120 was detected in the tissue samples during the analyses (presence of peaks at *m/z* 411.1751 shown in the red dotted box), whereas dabrafenib was not (absence of peaks at *m/z* 520.1083 in the tissue samples shown in the red dotted box).

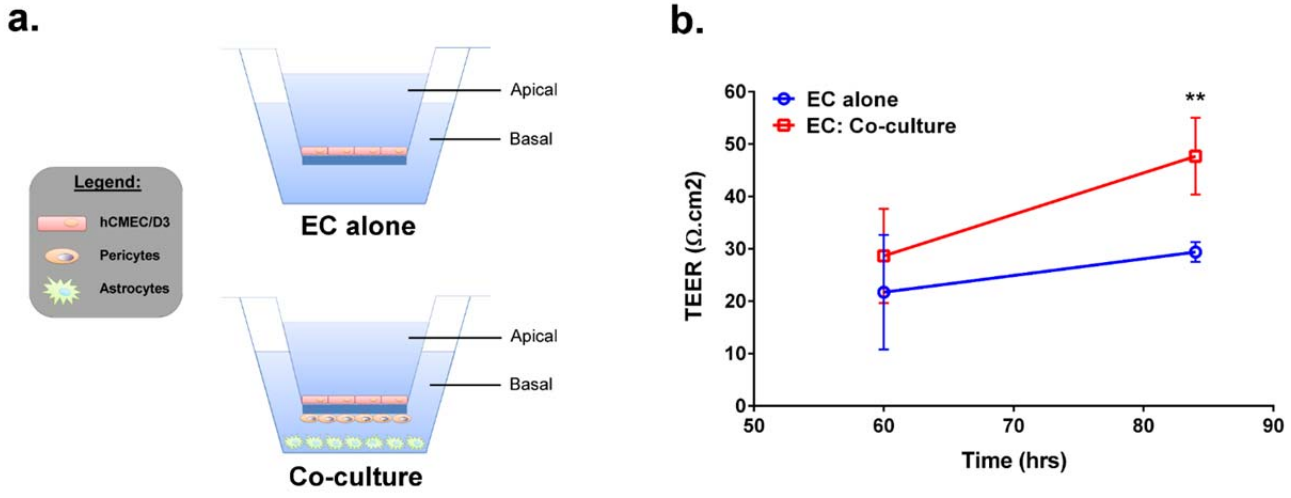

Supplementary Figure 7. Establishment of the BBB triple co-culture transwell model. (a) Schematic of the preparation of the *in vitro* BBB transwell model established by culturing either hCMEC/D3 endothelial cells (EC) alone (apical side of the insert), or in co-culture with human pericytes (basal side of the insert) and astrocytes (basal side on the bottom plate). Cells were co-cultured for 84 hours on a 24-well transwell system with inserts containing pore size of 0.4 µm. (b) Trans-endothelial Electrical Resistance (TEER) values of the transwell model using either hCMEC/D3 endothelial cells (ECs) cultured alone, or co-cultured with human pericytes and astrocytes as in (a). TEER measurements were obtained using the EVOM2 volt/ohm meter at 60 and 84 hrs. By 84 hrs of incubation, the co-culture model generated higher TEER values compared to EC alone ($n_{transwell} = 3$, $n_{experiment} = 3$). Statistical analysis was performed using the Sidak's multiple comparison test (** $p < 0.01$).

.

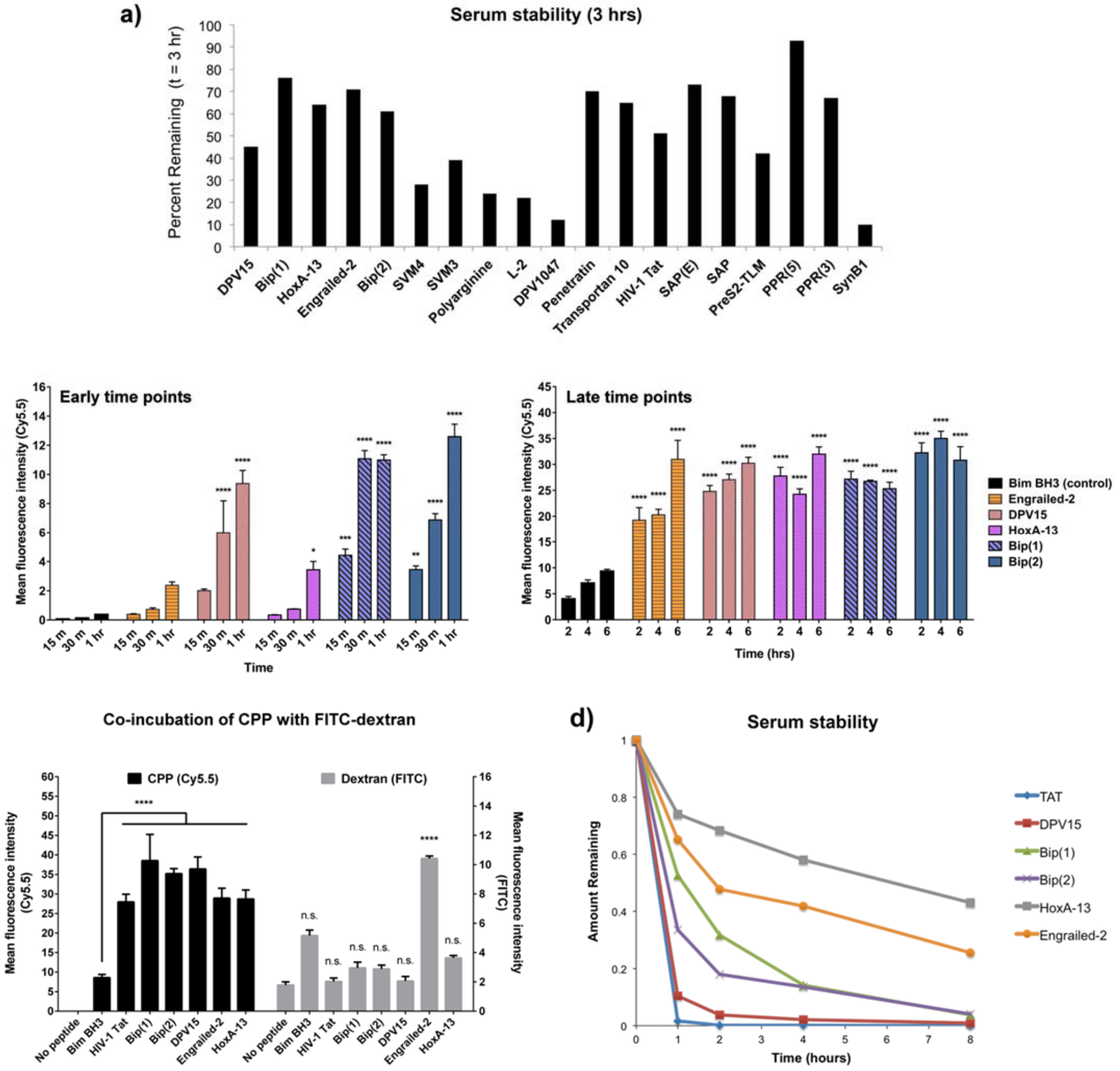

Supplementary Figure 8. Evaluation of cell-penetrating peptides (CPPs) for BBB penetration. (a) Serum stability of 19 Cy5.5-labeled CPPs in hCMEC/D3 working media containing 2% human serum (relative to Figure 6a). Each peptide (5 µM) was incubated for 3 hours at 37 °C, and then subjected to LC-MS analysis to quantify the percentage of intact peptide relative to t=0. (b) Time course analysis of the top 5 CPPs identified from (Fig. 3a) between 15 mins – 1 hour (early time points), and 2 – 6 hours (late time points). The graph displays mean fluorescence intensity quantified at 88 µm depth from the surface of each spheroid with SD error bars ($n_{spheroid}$ = 4-6). The significance of each value is compared with the Bim BH3 peptide (control with no cell-penetrating ability) (* $p < 0.05$; ** $p < 0.01$; *** $p < 0.001$; **** $p < 0.0001$). (c) Analysis of the top CPPs and their effect on the barrier integrity of BBB spheroids. Spheroids were co-incubated with the top CPPs (5 µM) and FITC-dextran (70 kDa) (10 µg/mL) for 3 hrs. The graph displays mean fluorescence intensity of the CPPs (Cy5.5) and dextran (FITC) at 88 µm depth from the surface of each spheroid with SD error bars ($n_{spheroid}$ = 3-6). Significance of CPP influx is compared with Bim BH3 control, while significance of dextran influx is compared with no peptide control. Statistics were performed using the two-way ANOVA and Dunnett's multiple comparison test ($p < 0.0001$). Incubation of spheroids with Engrailed-2 peptide significantly increased spheroid permeability to FITC-dextran. (d) Serum stability of the top Cy5.5-labeled CPPs in 10% mouse serum. Each peptide (50 µM) was incubated in PBS containing 10% mouse serum at 37 ºC. At the indicated times, an aliquot was subjected to liquid

chromatography-mass spectrometry (LC-MS) analysis to quantify the amount of peptide that remained relative to t=0.

Supplementary Notes.

Amino acid sequences, HPLC chromatograms and mass spectra of all proteins and peptides used in this study are depicted below. The C-terminus is an amide (-CO-NH2) unless otherwise noted. The underlined portion of the amino acid sequences corresponds to the angiopep or angiopep-scrambled portion of the conjugate. For details on synthesis, please see the main text or supplementary information.

The following liquid chromatography (LC) method was used:

A = water 0.1% formic acid; B = acetonitrile 0.1% formic acid; flow rate = 0.8 mL/min
0-2 minutes 5% B,
2-11 minutes linear gradient 5-65% B
11-12 minutes 65% B
Column: Zorbax 300SB C_3 column (2.1 x 150mm, 5μm), 40ºC

(1)

TAMRA-angiopep (Mass expected: 2711.6 Da; Mass observed 2711.2 Da)
TAMRA-<u>TFFYGGSRGKRNNFKTEEY</u>

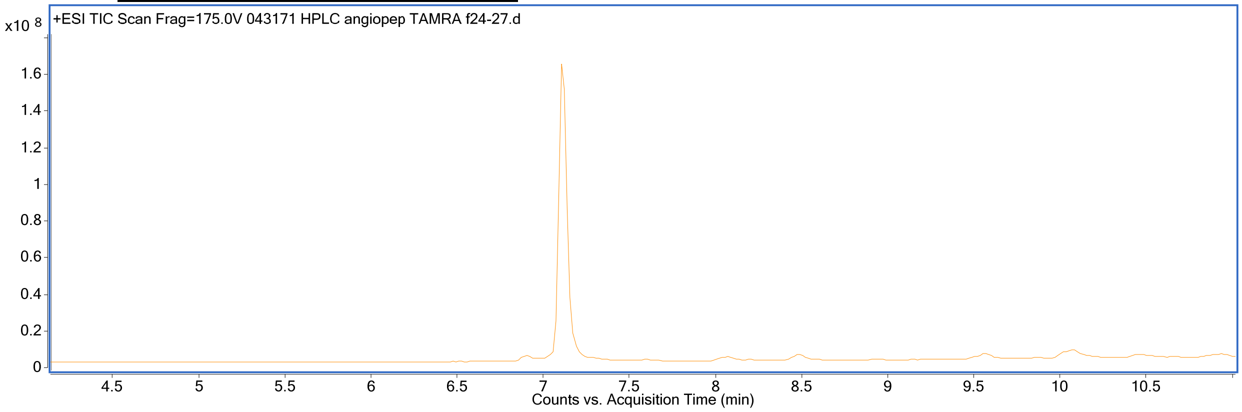

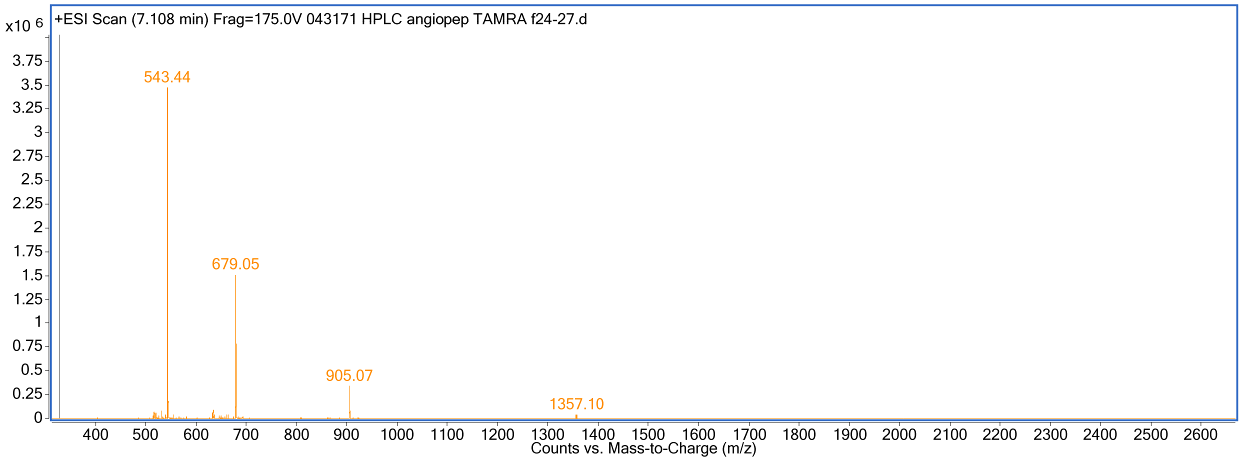

(2)

TAMRA-scrambled angiopep (Mass expected: 2711.6 Da; Mass observed 2711.3 Da)
TAMRA-GNYTSRFEREYGKFNKFGT

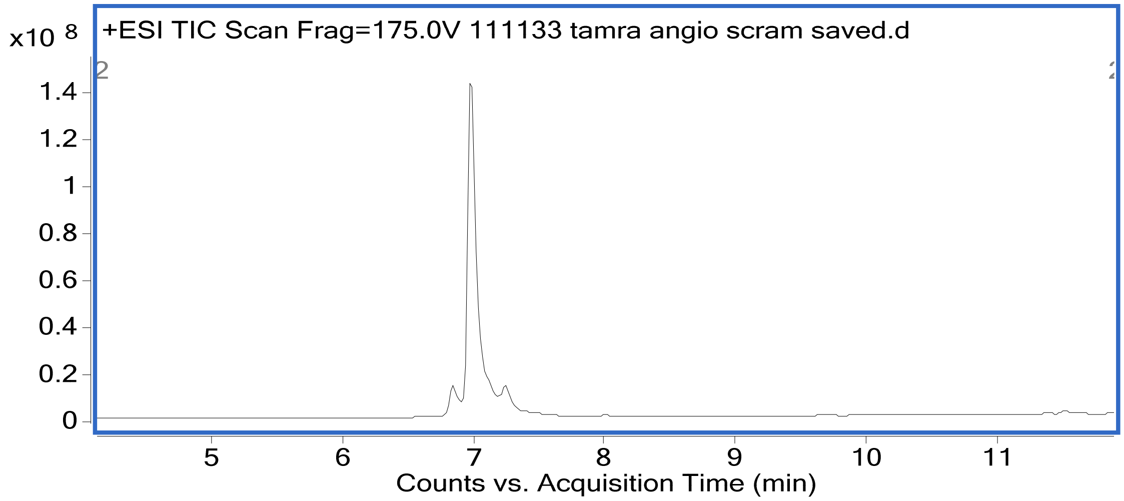

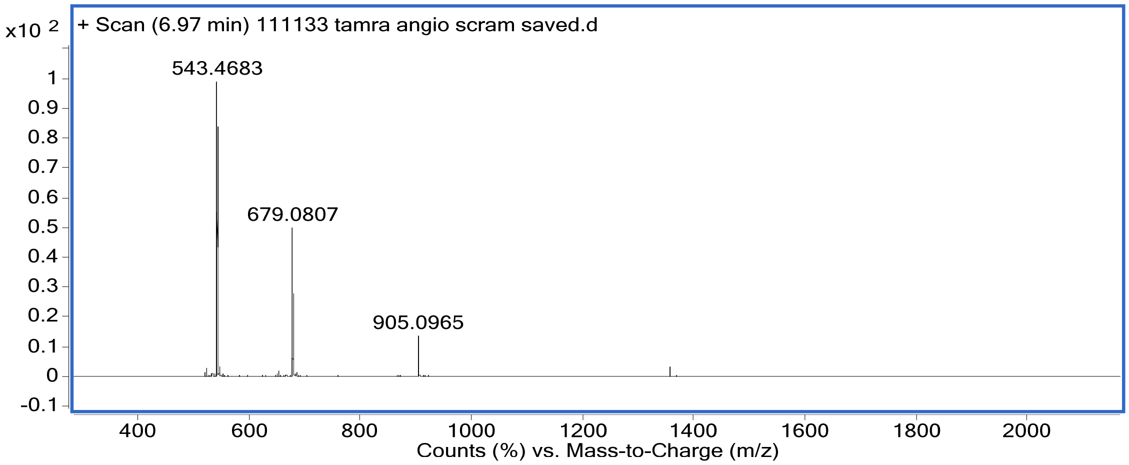

(3)

Cy5-angiopep (Mass expected: 2764.7 Da; Mass observed 2764.4 Da)
Cy5-TFFYGGSRGKRNNFKTEEY

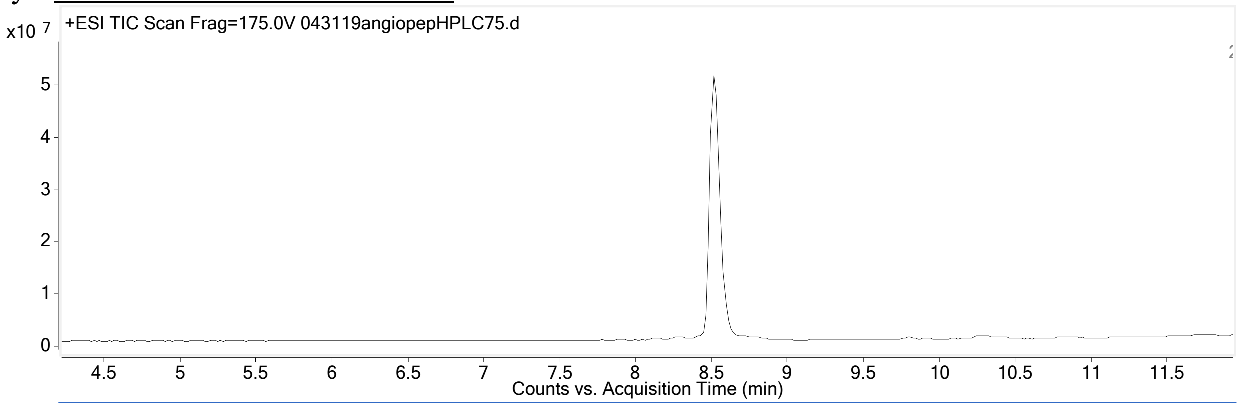

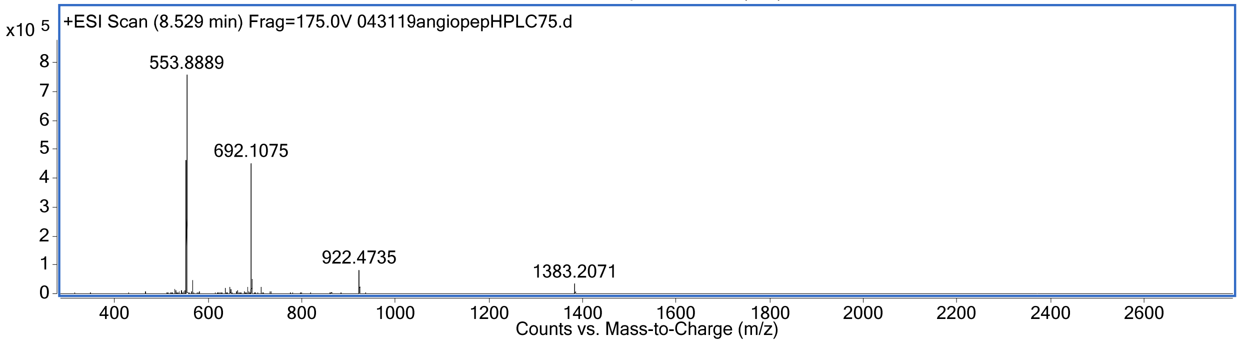

(4)

Cy5-scrambled angiopep (Mass expected: 2764.7 Da; Mass observed 2764.4 Da)
Cy5- GNYTSRFEREYGKFNKFGT

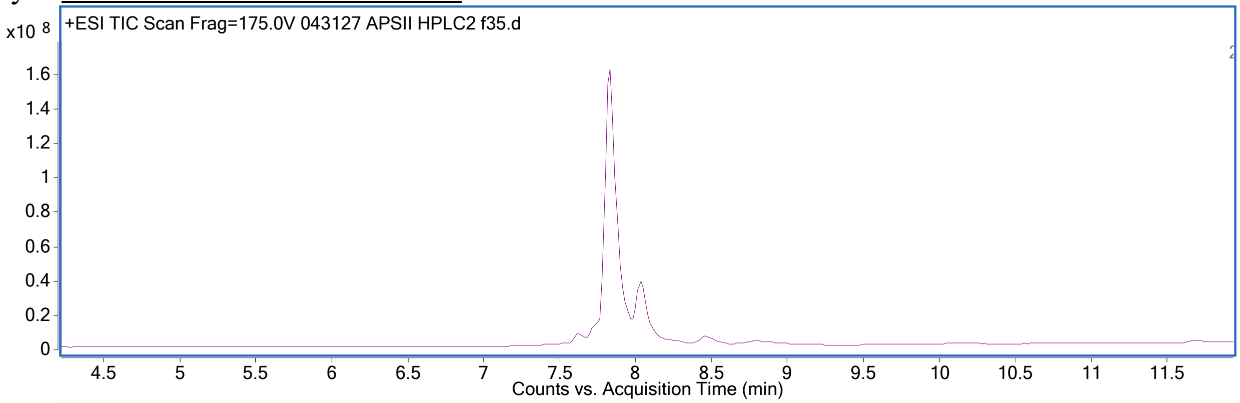

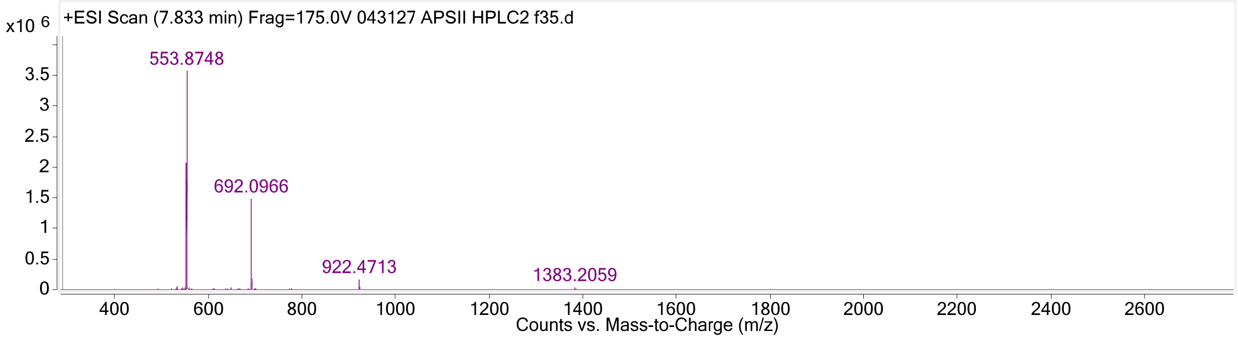

(5)

Cy5.5-angiopep (Mass expected: 3044.5 Da; Mass observed 3044.5 Da)
Cy5.5-TFFYGGSRGKRNNFKTEEY

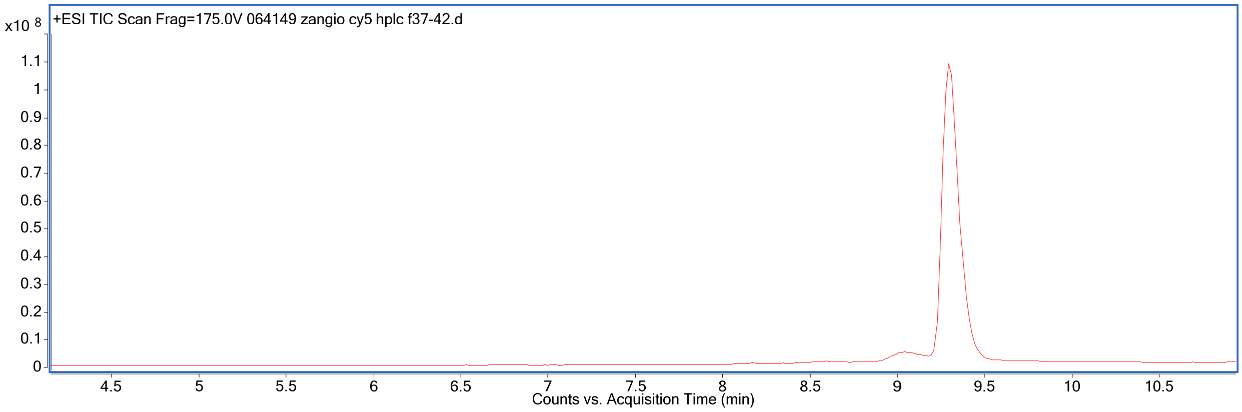

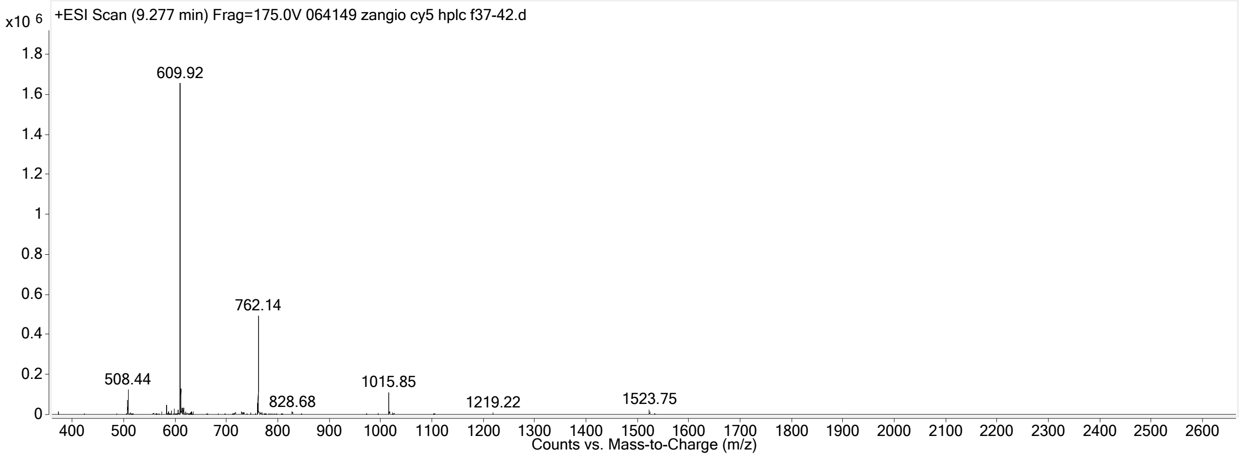

(6)

Cy5.5-scrambled angiopep (Mass expected: 3044.5 Da; Mass observed 3044.5 Da)
Cy5.5- GNYTSRFEREYGKFNKFGT

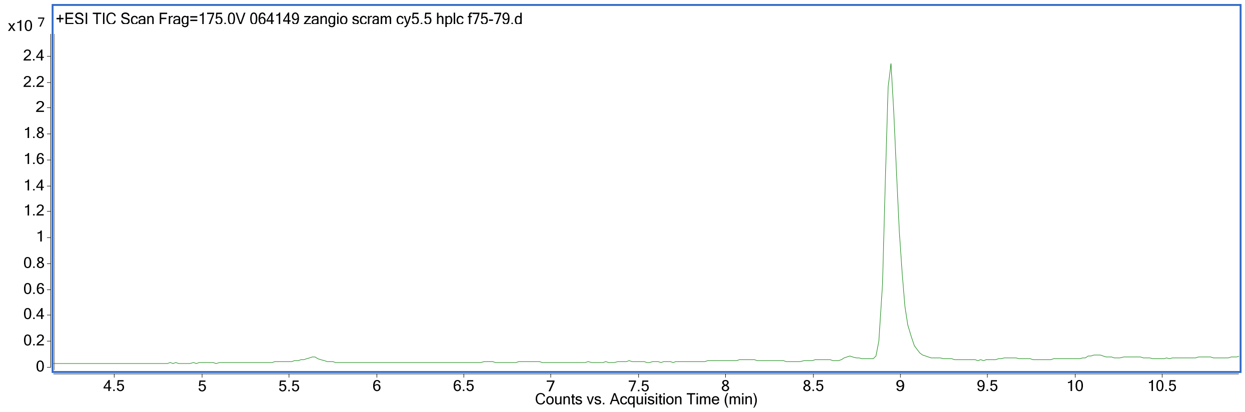

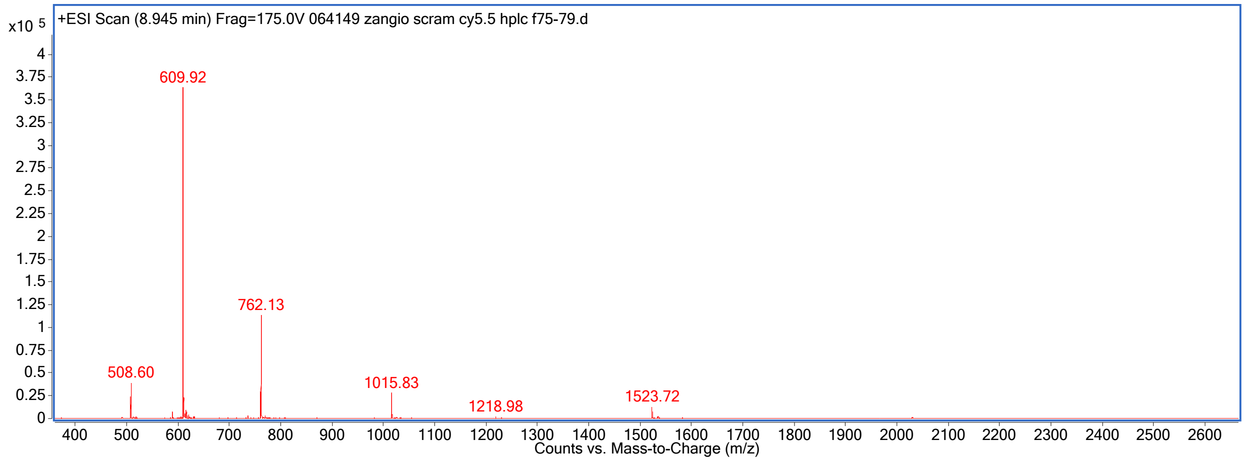

(7)

TAMRA-angiopep-Bim BH3 (Mass expected: 6644.5 Da; Mass observed 6645.2 Da)
TAMRA-TFFYGGSRGKRNNFKTEEYLPSTGGGGGDMRPEIWIAQELRRIGDEFNAYYARR

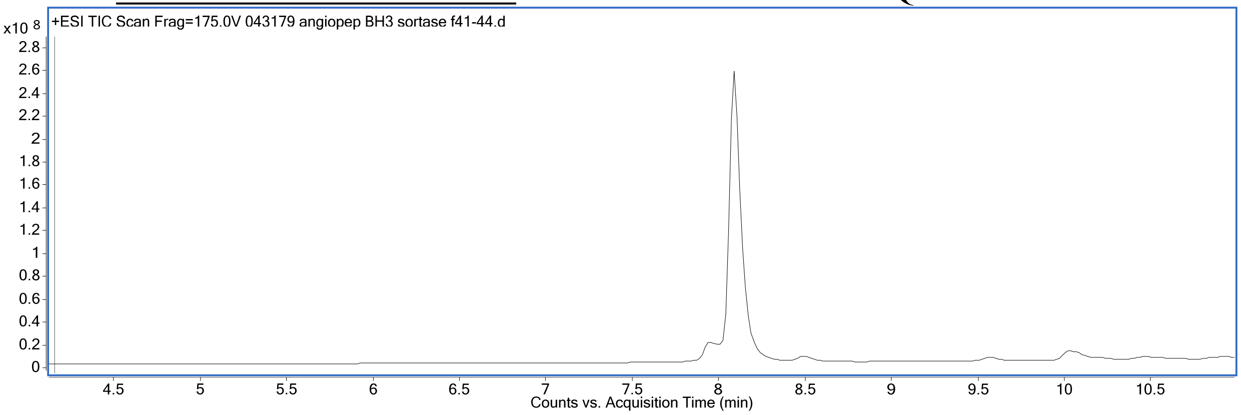

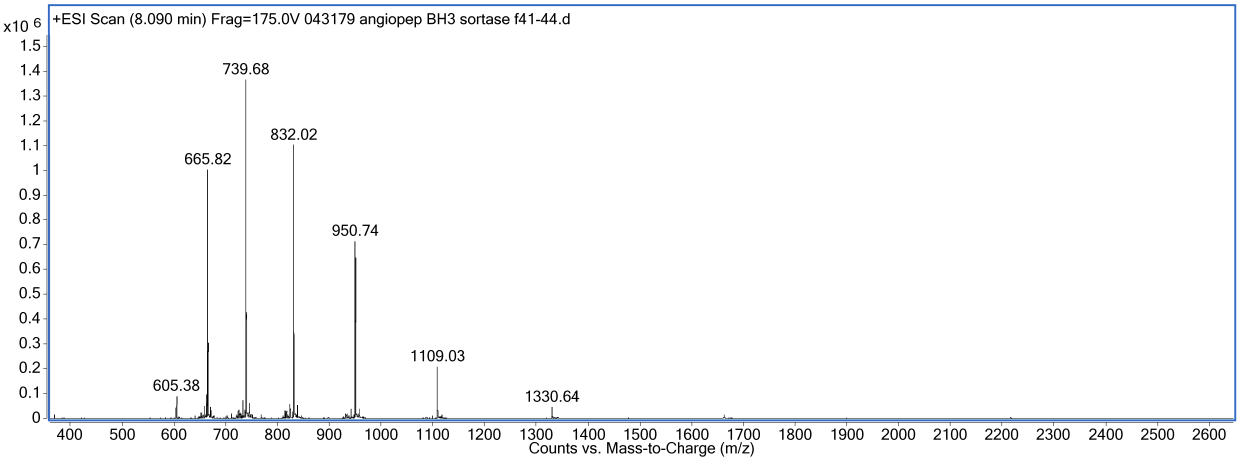

(8)

TAMRA-scrambled angiopep-Bim BH3 (Mass expected: 6644.5 Da; Mass observed 6645.2 Da)
TAMRA-GNYTSRFEREYGKFNKFGTLPSTGGGGGDMRPEIWIAQELRRIGDEFNAYYARR

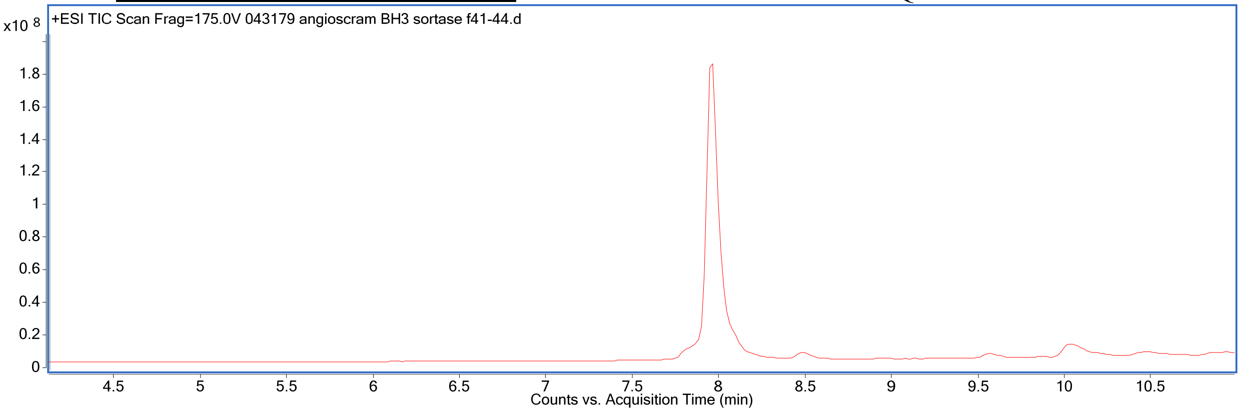

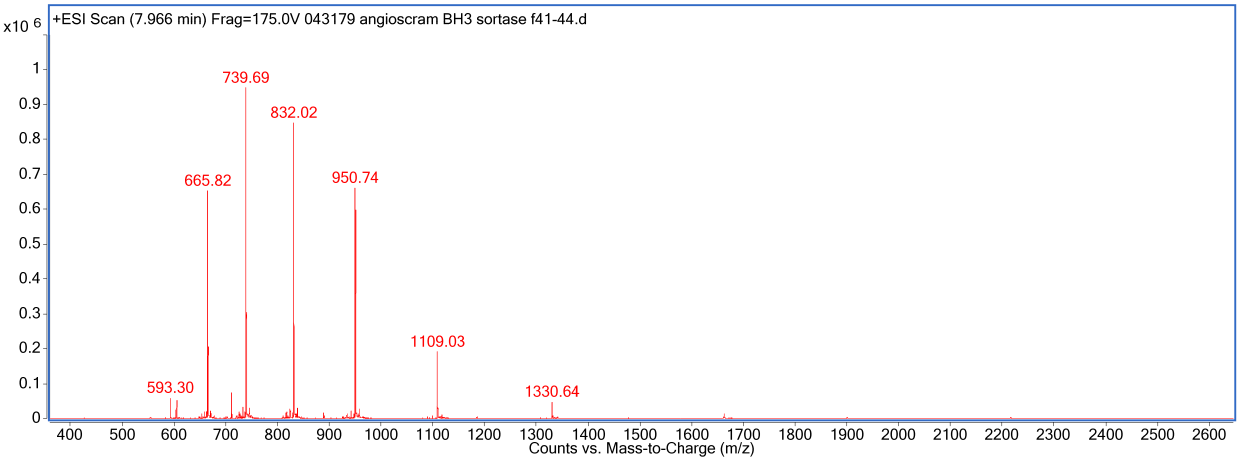

(9)

eGFP-Angio (Mass expected: 29888.9 Da; Mass observed 29887.8 Da)
MVSKGEELFTGVVPILVELDGDVNGHKFSVSGEGEGDATYGKLTLKFICTTGKLPVPWPTLVTT
LTYGVQCFSRYPDHMKQHDFFKSAMPEGYVQERTIFFKDDGNYKTRAEVKFEGDTLVNRIELK
GIDFKEDGNILGHKLEYNYNSHNVYIMADKQKNGIKVNFKIRHNIEDGSVQLADHYQQNTPIGD
GPVLLPDNHYLSTQSALSKDPNEKRDHMVLLE
FVTAAGITLGMDELYKLPSTGGGGGTFFYGGSRGKRNNFKTEEY

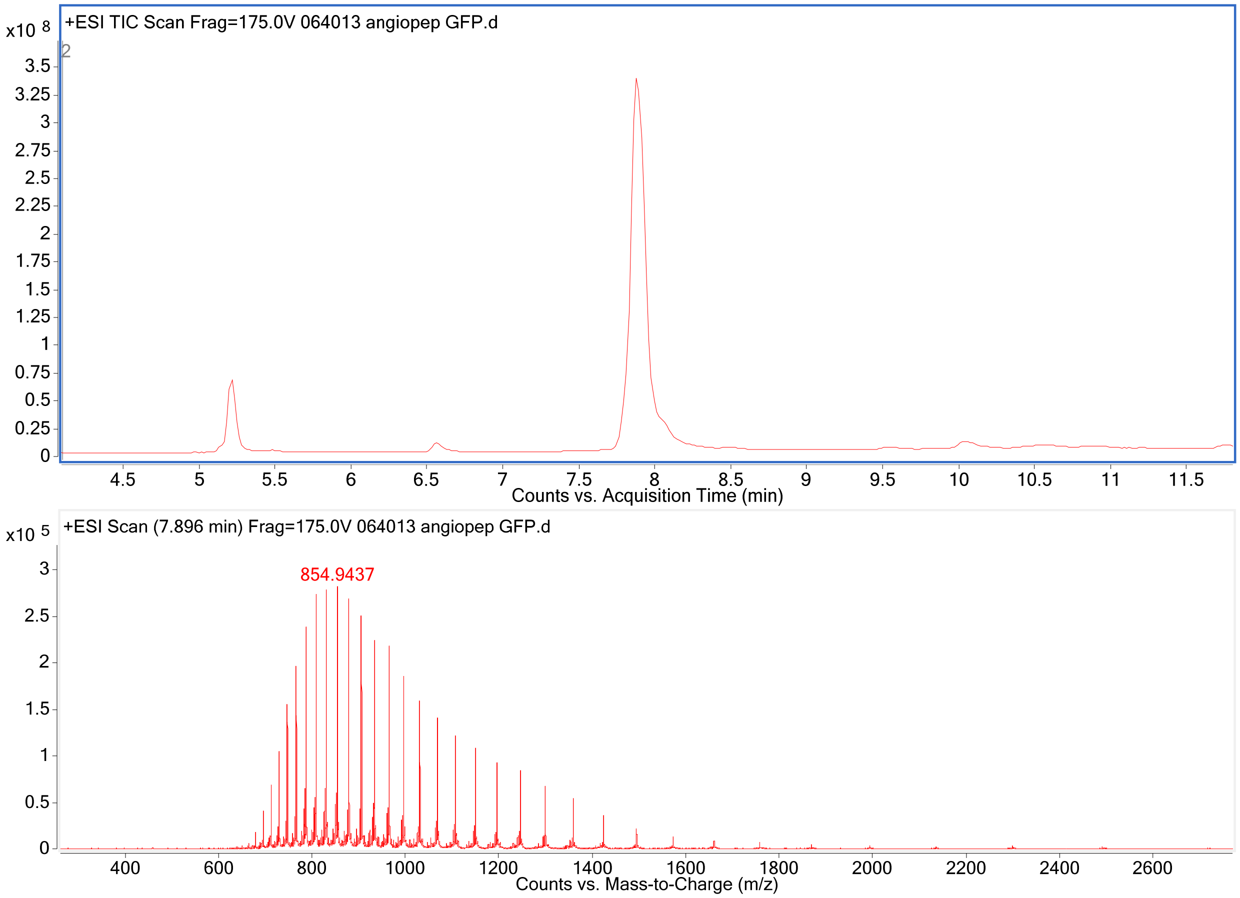

(10)

TAMRA-Angio-affibody (Mass expected: 11104.2 Da; Mass observed 11104.4 Da)
TAMRA-
TFFYGGSRGKRNNFKTEEYLPSTGGGGGVDNKFNKEQQNAFYEILHLPNLNEEQRNAFIQSLKD
DPSQSANLLAEAKKLNDAQAPKYPYDVPDYA*

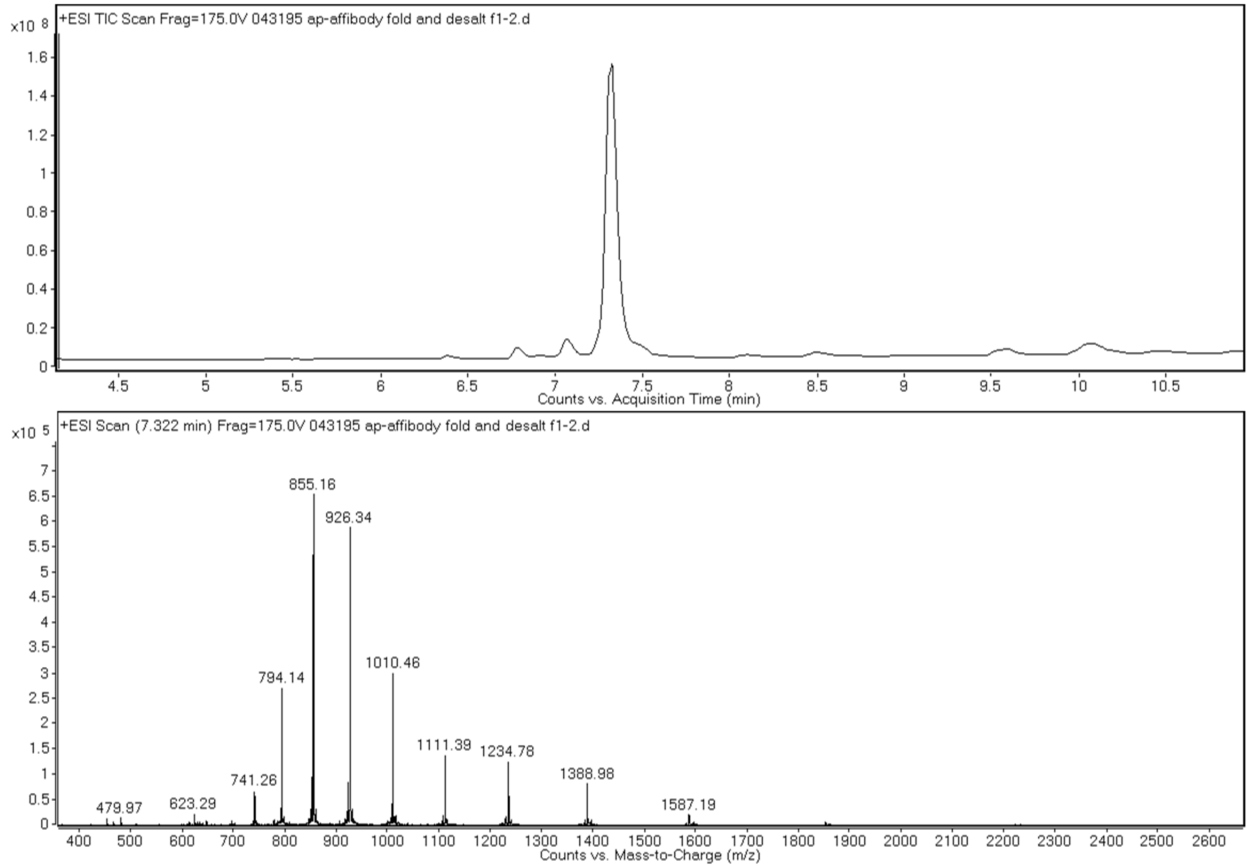

*-*COOH*

(11)

TAMRA-Affibody (Mass expected: 8820.7 Da; Mass observed: 8820.5 Da)
TAMRA-
LPSTGGGGGVDNKFNKEQQNAFYEILHLPNLNEEQRNAFIQSLKDDPSQSANLLAEAKKLNDA
QAPKYPYDVPDYA*

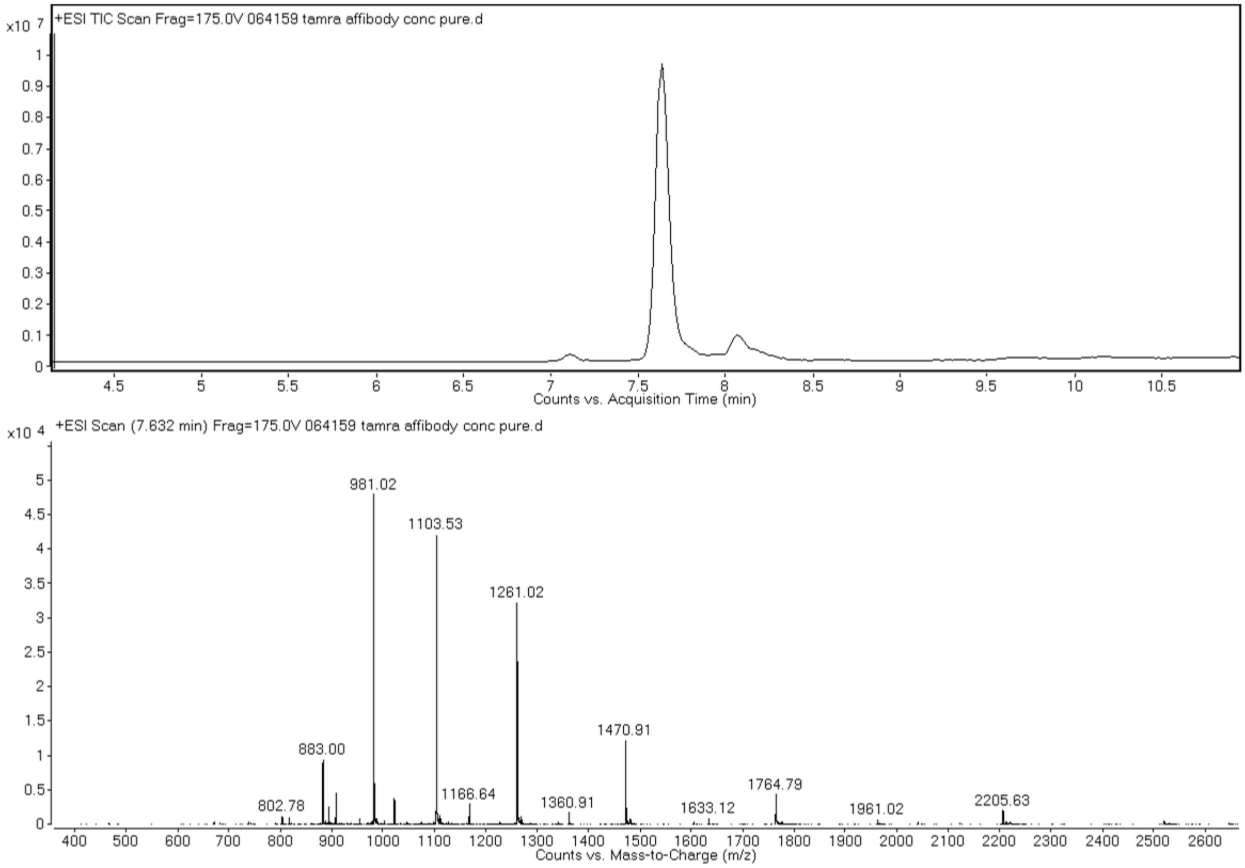

*-*COOH*

(12)

Peptide: DPV15
Sequence: Cy5.5-triazole-LRRERQSRLRRERQSR

Expected MW: 2925.7 Da
Observed MW: 2925.7 Da

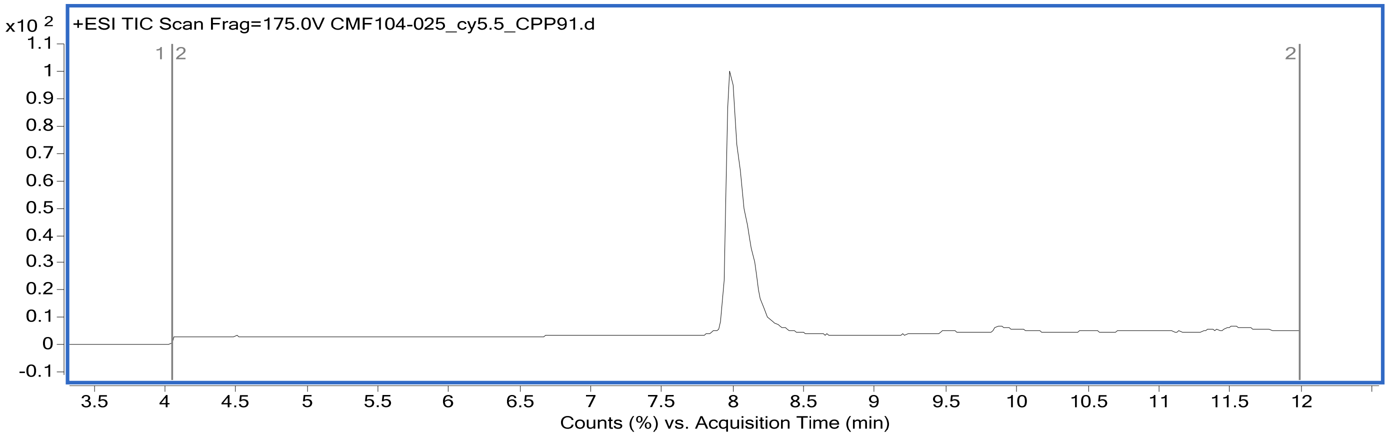

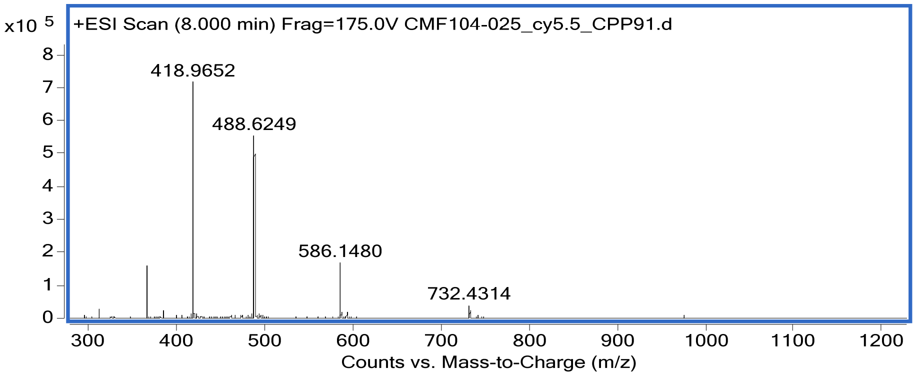

(13)

Peptide: Bip (1)
Sequence: Cy5.5-triazole-VPALR

Expected MW: 1298.8 Da
Observed MW: 1298.8 Da

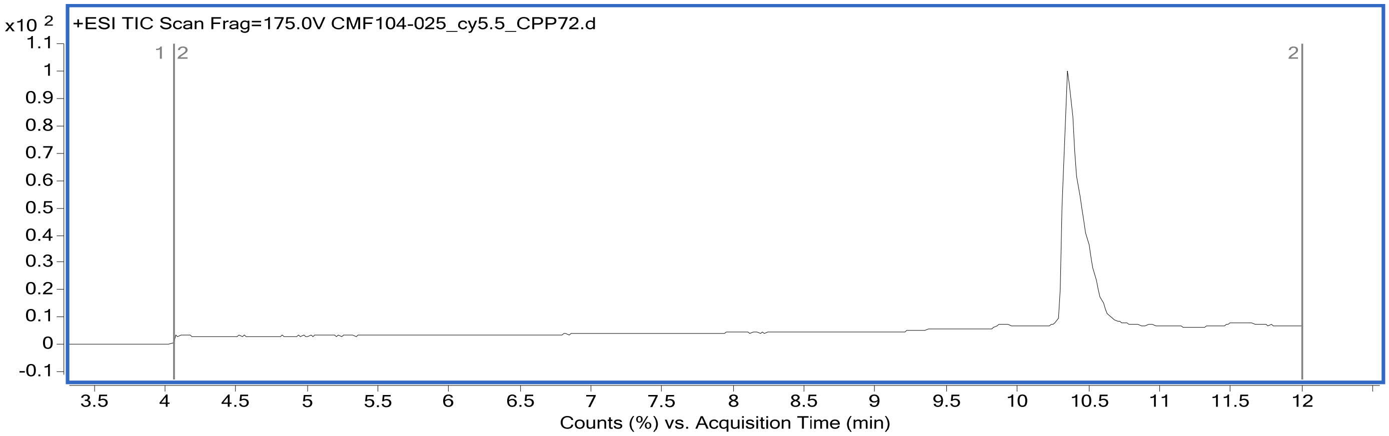

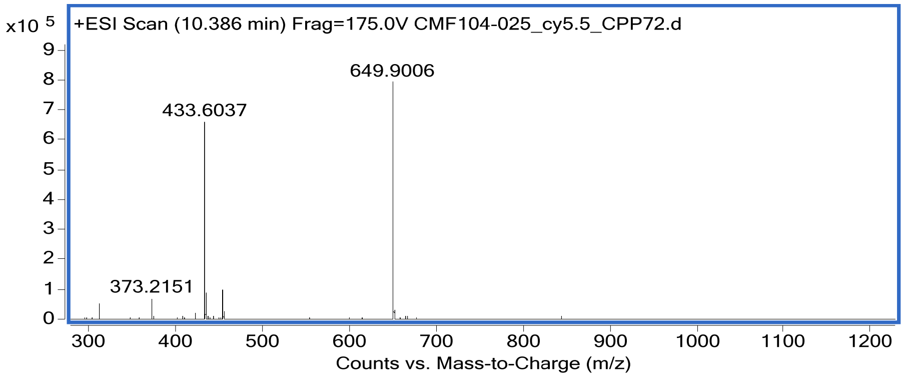

(14)

Peptide: HoxA-13
Sequence: Cy5.5-triazole-RQVTIWFQNRRVKEKK

Expected MW: 2859.6 Da
Observed MW: 2859.7 Da

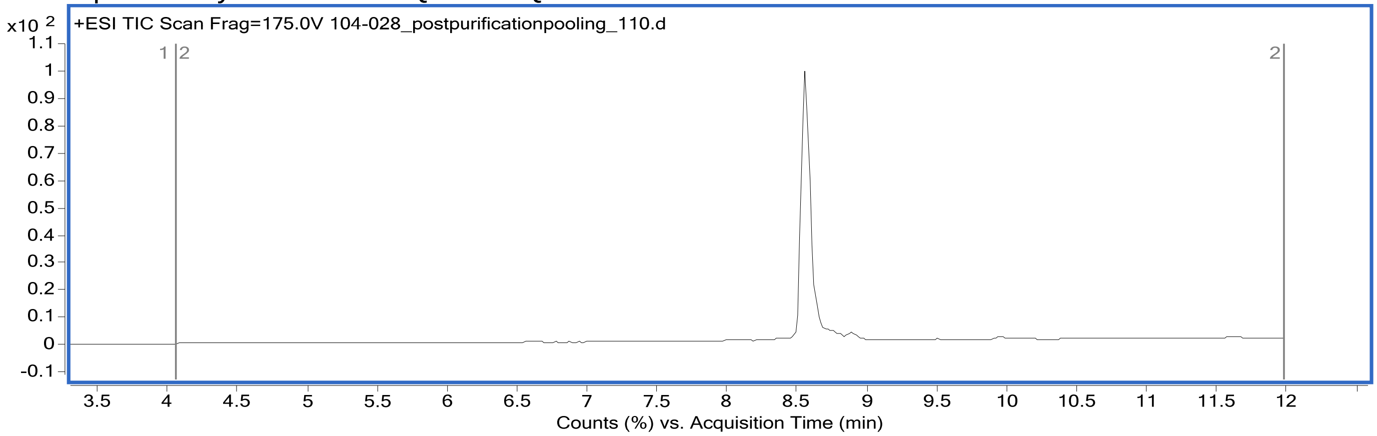

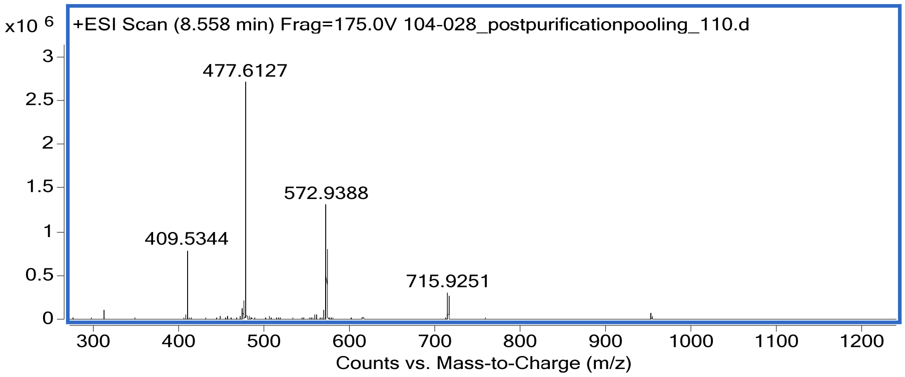

(15)

Peptide: Engrailed-2
Sequence: Cy5.5-triazole-SQIKIWFQNKRAKIKK

Expected MW: 2759.6 Da
Observed MW: 2759.6 Da

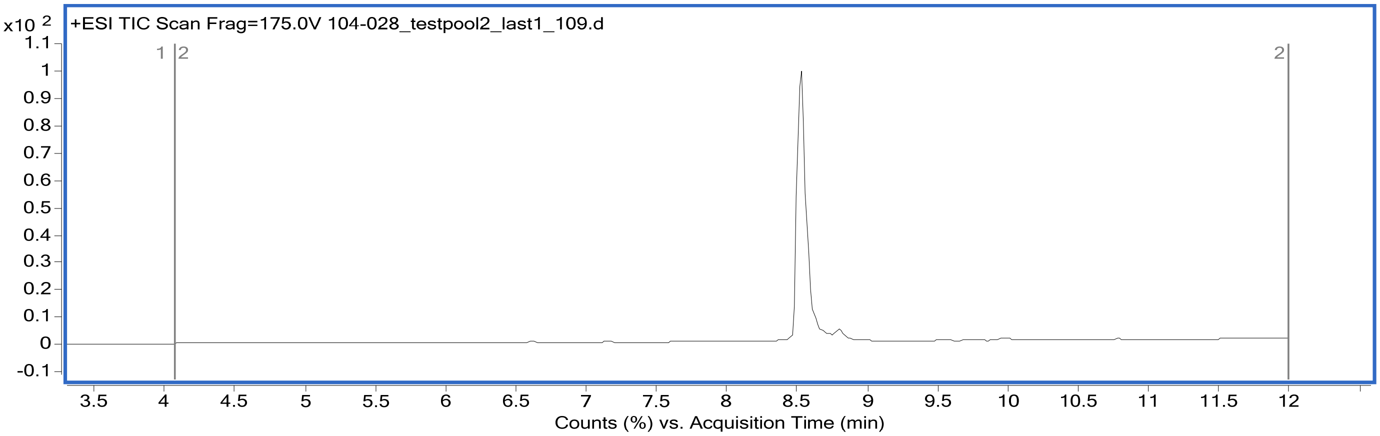

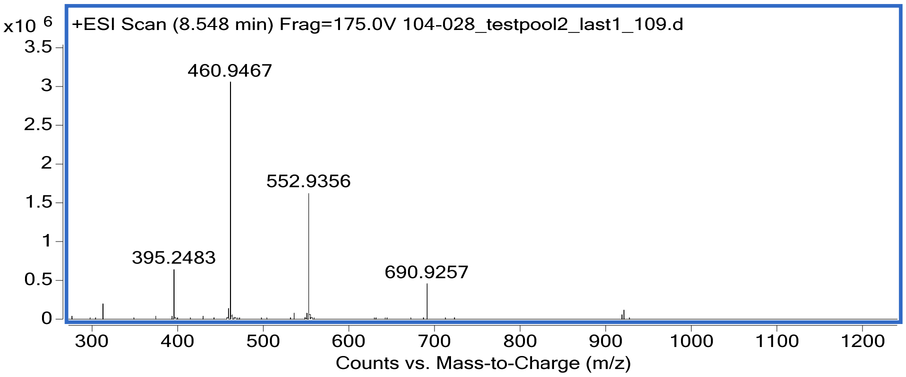

(16)

Peptide: Bip (2)
Sequence: Cy5.5-triazole-VSALK

Expected MW: 1260.7 Da
Observed MW: 1260.8 Da

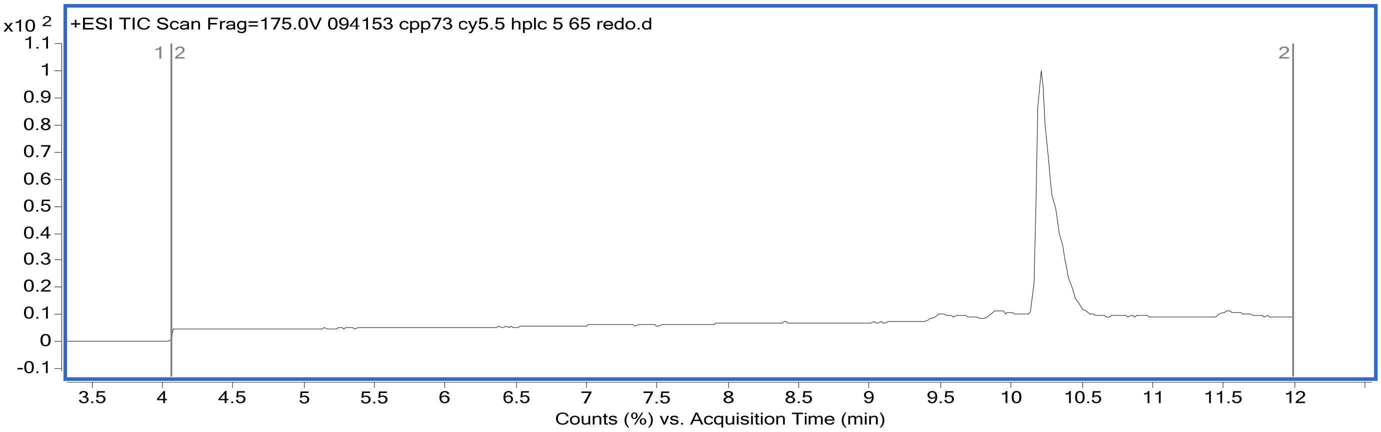

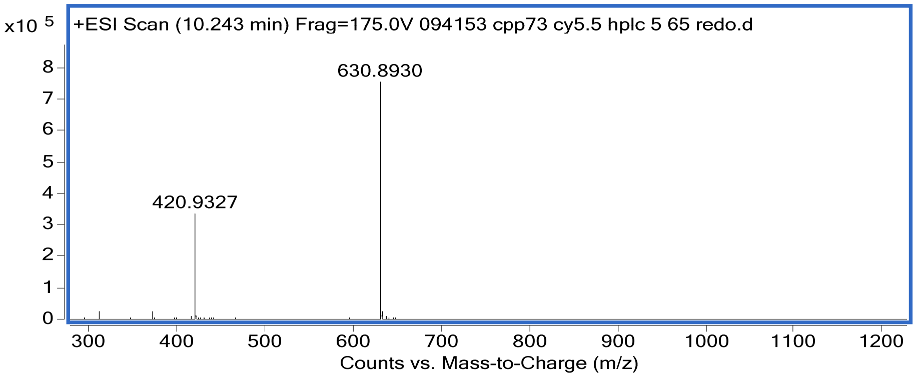

(17)

Peptide: SVM4 Expected MW: 2979.7 Da
Sequence: Cy5.5-triazole-LYKKGPAKKGRPPLRGWFH Observed MW: 2979.7 Da

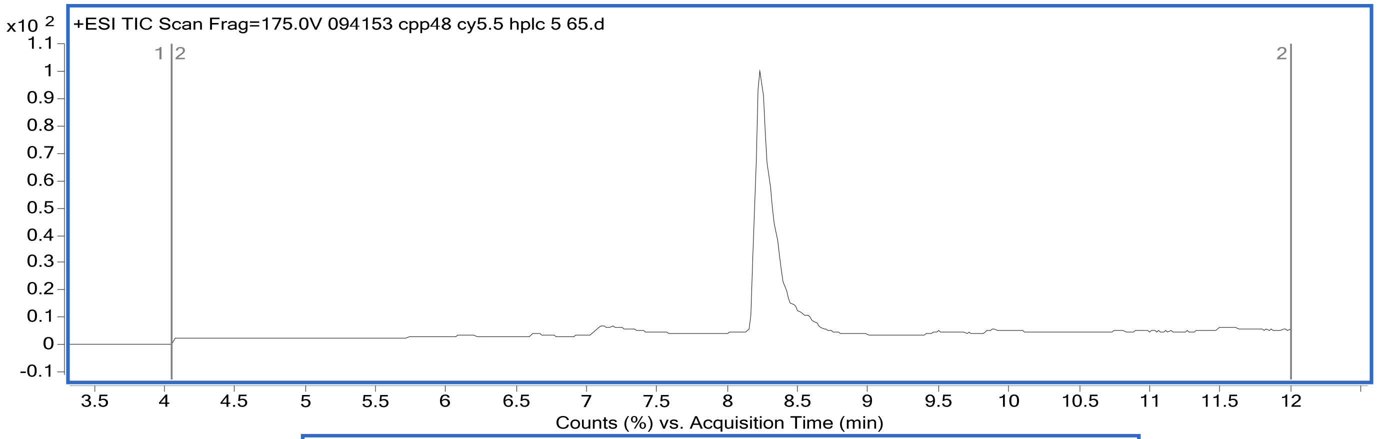

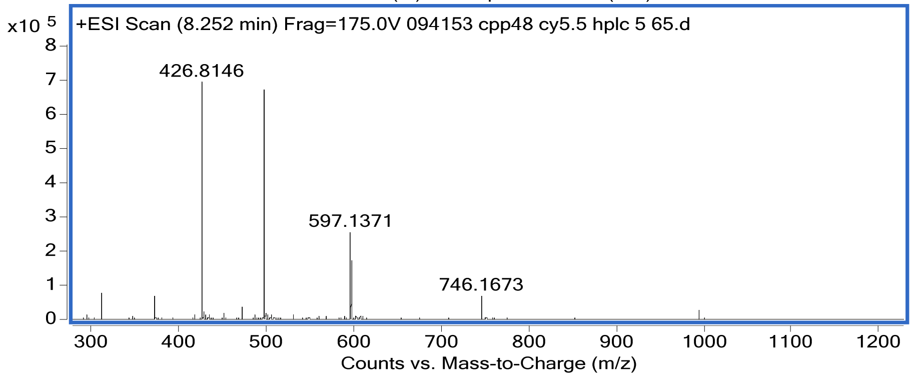

(18)

Peptide: SVM3
Sequence: Cy5.5-triazole-KGTYKKKLMRIPLKGT

Expected MW: 2605.5 Da
Observed MW: 2605.6 Da

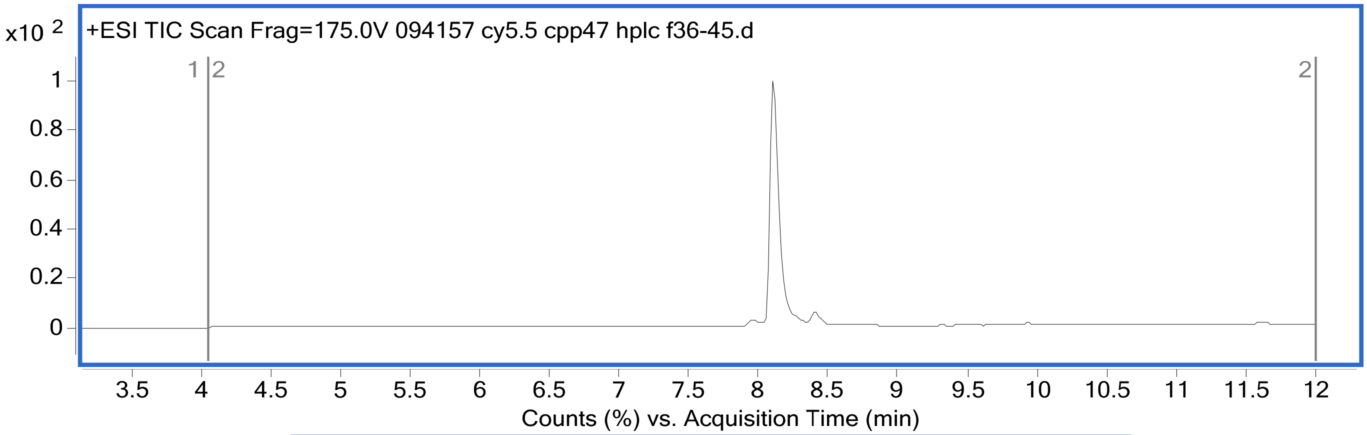

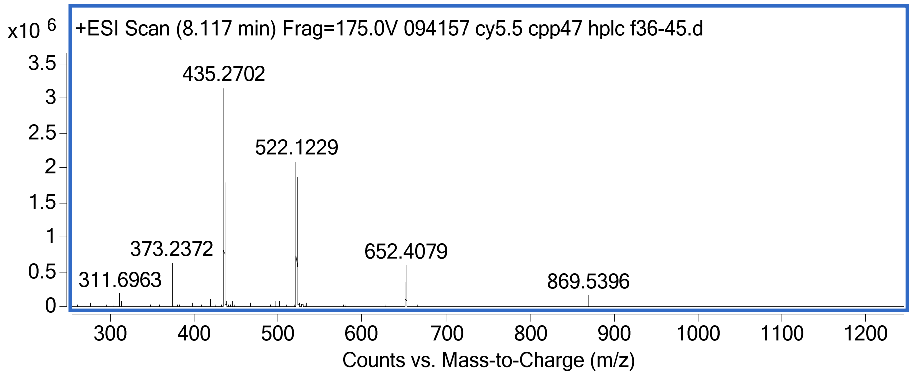

(19)

Peptide: Polyarginine
Sequence: Cy5.5-triazole-RRRRRRRR

Expected MW: 2011.2 Da
Observed MW: 2011.3 Da

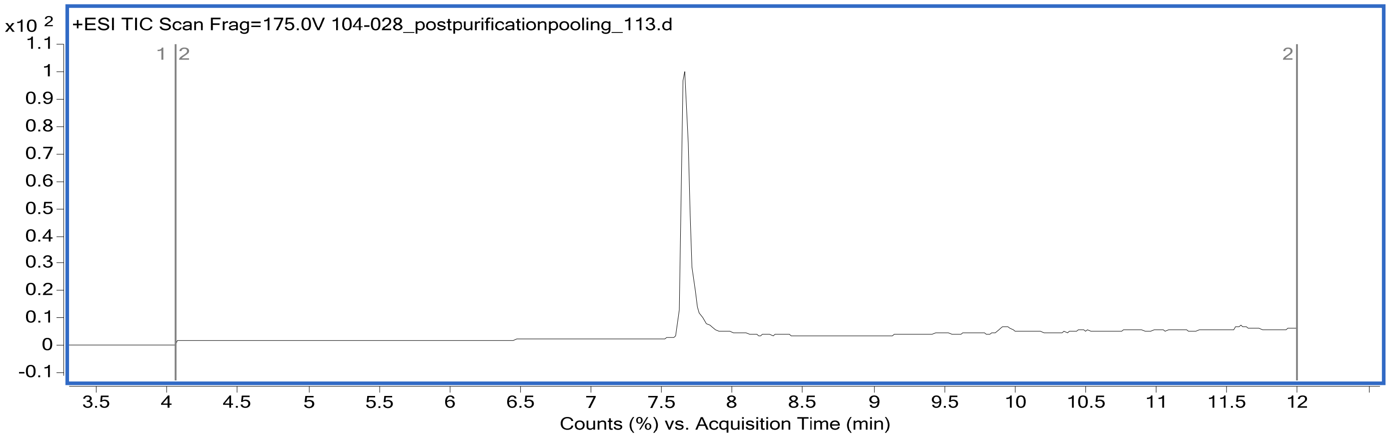

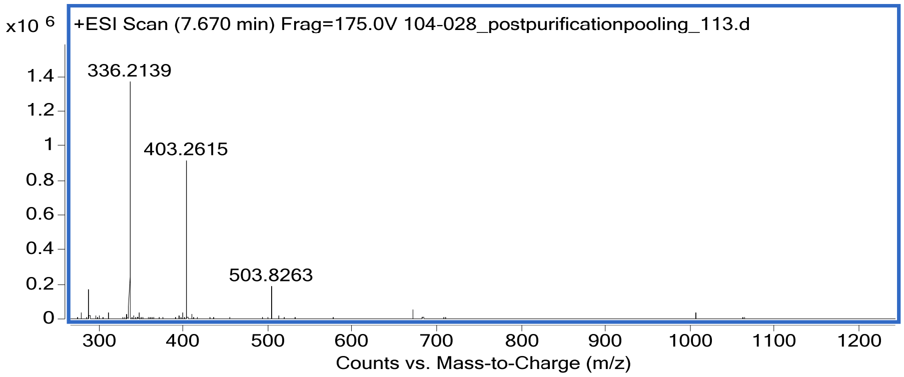

(20)

Peptide: L-2
Sequence: Cy5.5-triazole-HARIKPTFRRLKWKYKGKFW

Expected MW: 3389.9 Da
Observed MW: 3390.0 Da

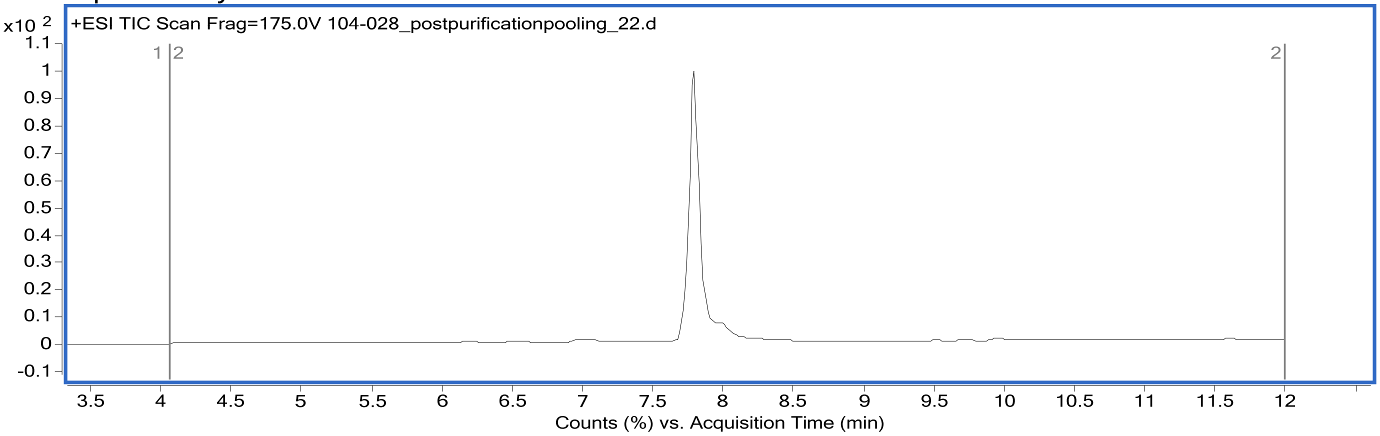

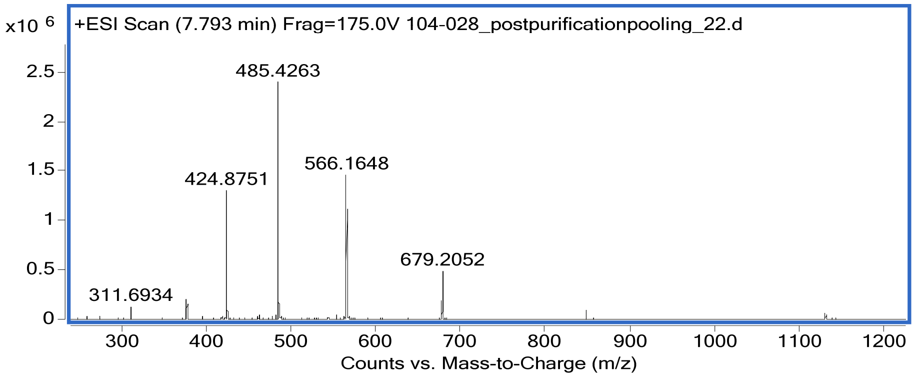

(21)

Peptide: DPV1047
Sequence: Cy5.5-triazole-VKRGLKLRHVRPRVTRMDV

Expected MW: 3059.8 Da
Observed MW: 3059.9 Da

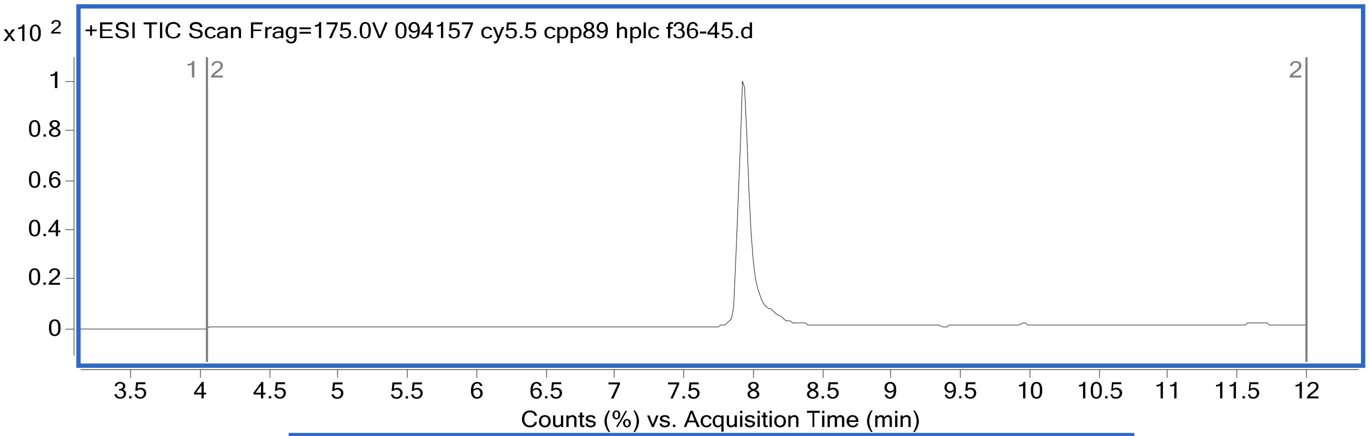

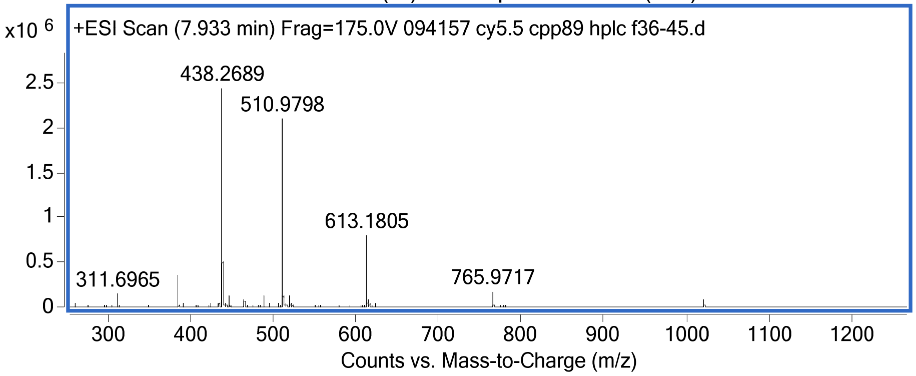

(22)

Peptide: Penetratin
Sequence: Cy5.5-triazole-RQIKIWFQNRRMKWKK

Expected MW: 2989.7 Da
Observed MW: 2989.8 Da

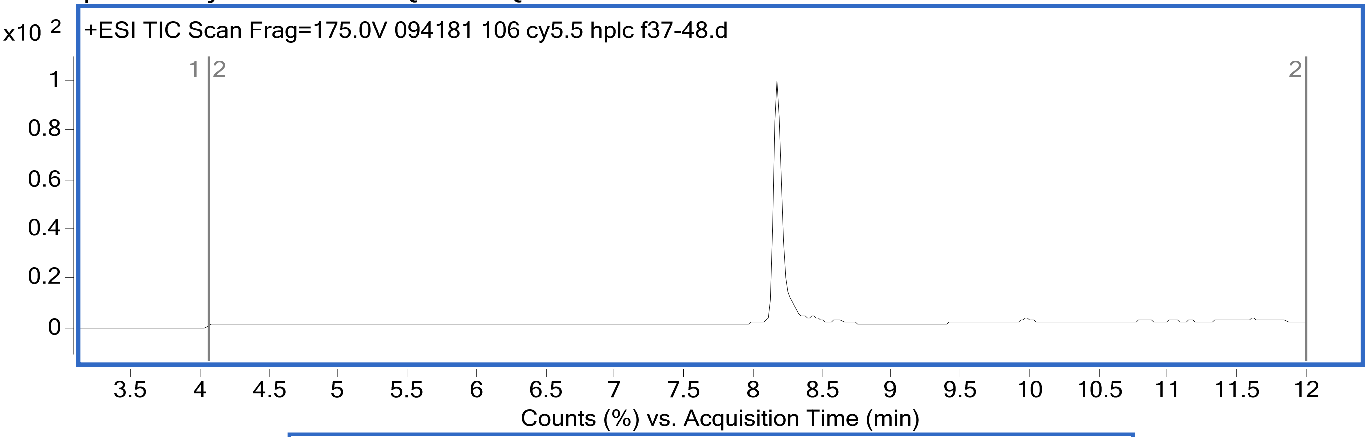

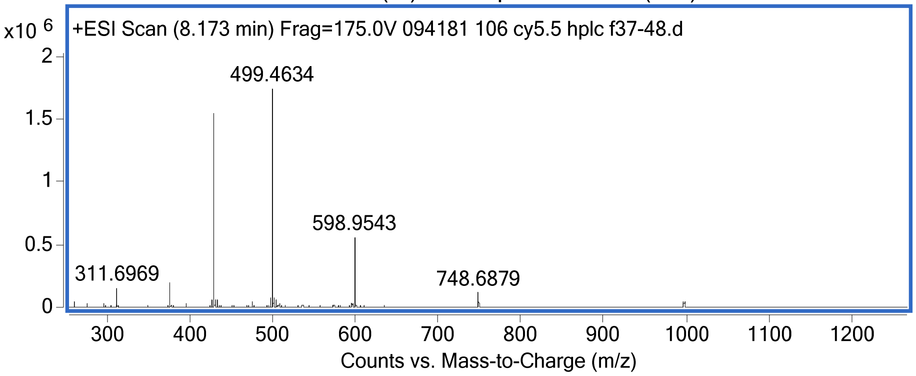

(23)

Peptide: TP10
Sequence: Cy5.5-triazole-AGYLLGKINLKALAALAKKIL

Expected MW: 2925.8 Da
Observed MW: 2925.9 Da

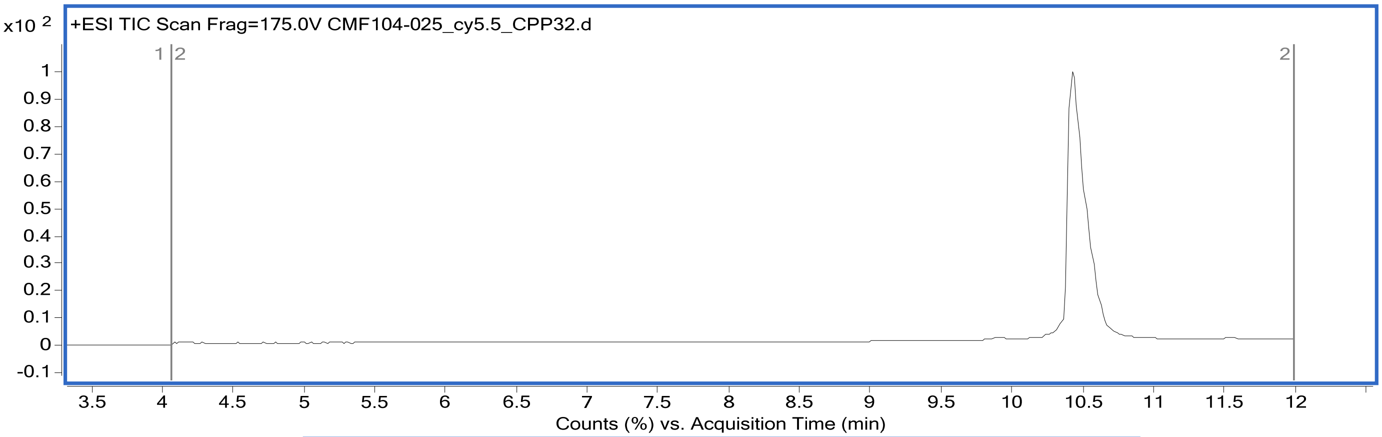

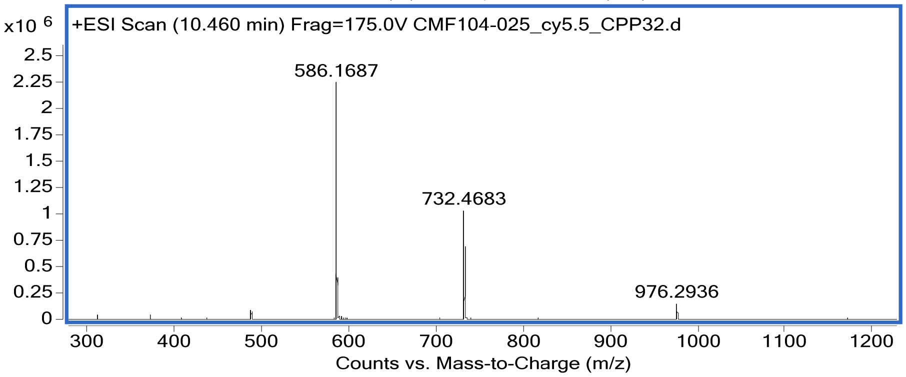

(24)

Peptide: HIV-1 TAT
Sequence: Cy5.5-triazole-RKKRRQRRR

Expected MW: 2083.3 Da
Observed MW: 2083.3 Da

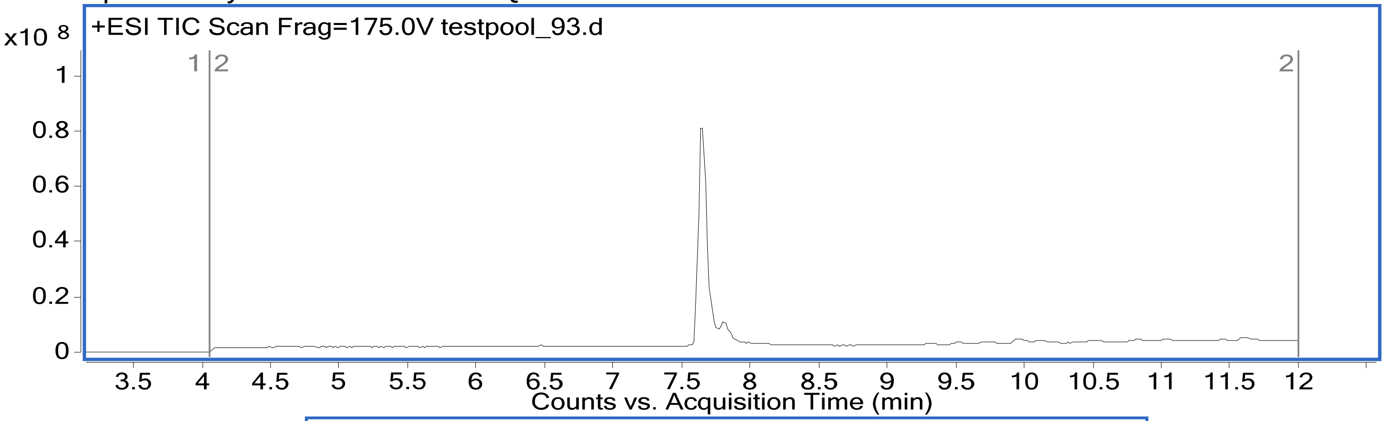

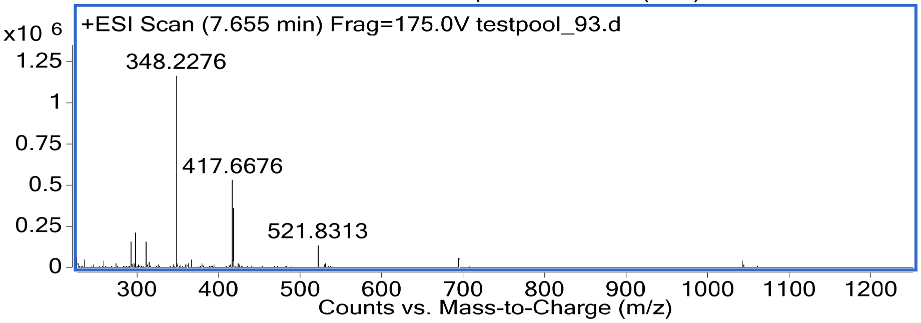

(25)

Peptide: SAP(E)
Sequence: Cy5.5-triazole-VELPPPVELPPPVELPPP

Expected MW: 2659.5 Da
Observed MW: 2659.5 Da

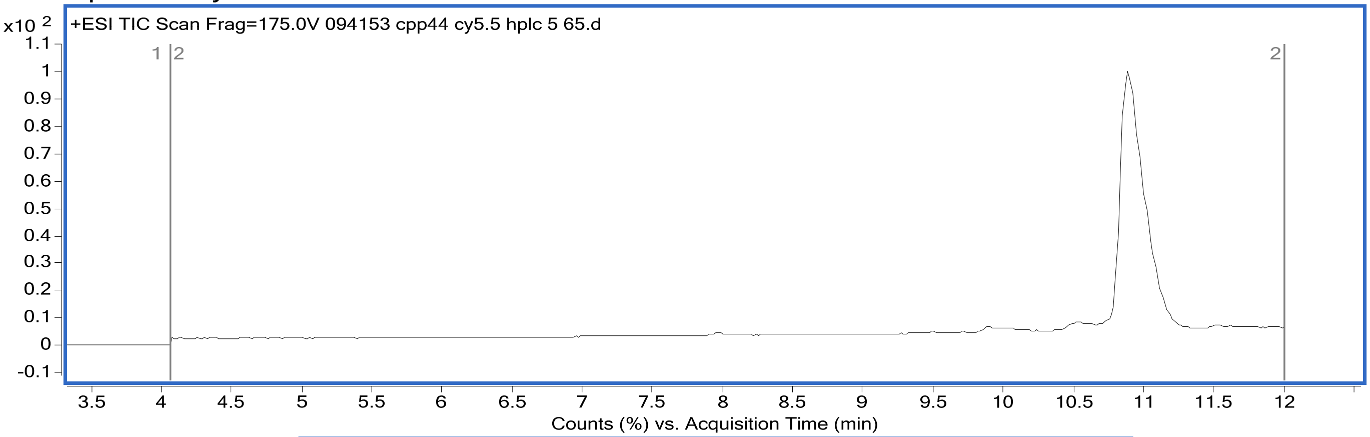

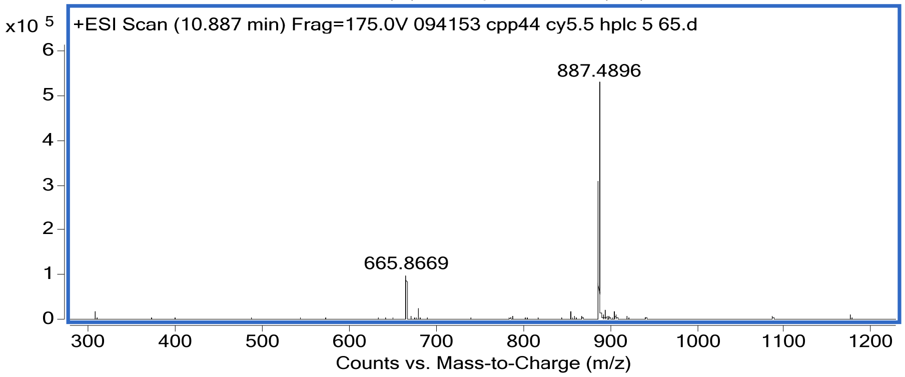

(26)

Peptide: SAP
Sequence: Cy5.5-triazole-VRLPPPVRLPPPVRLPPP

Expected MW: 2740.7 Da
Observed MW: 2740.6 Da

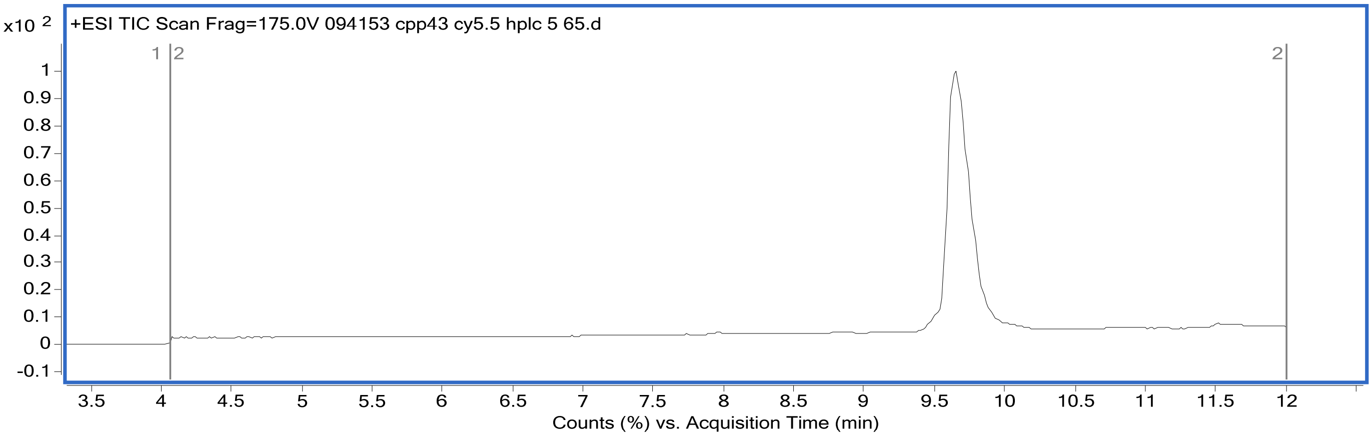

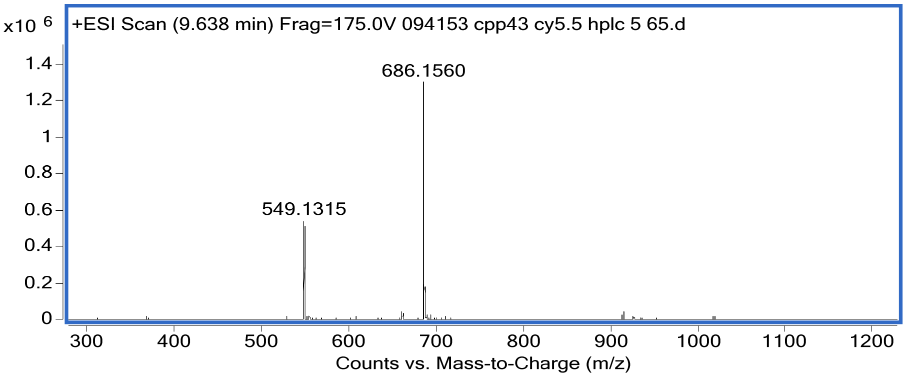

(27)

Peptide: PreS2-TLM
Sequence: Cy5.5-triazole-PLSSIFSRIGDP

Expected MW: 2032.1 Da
Observed MW: 2032.1 Da

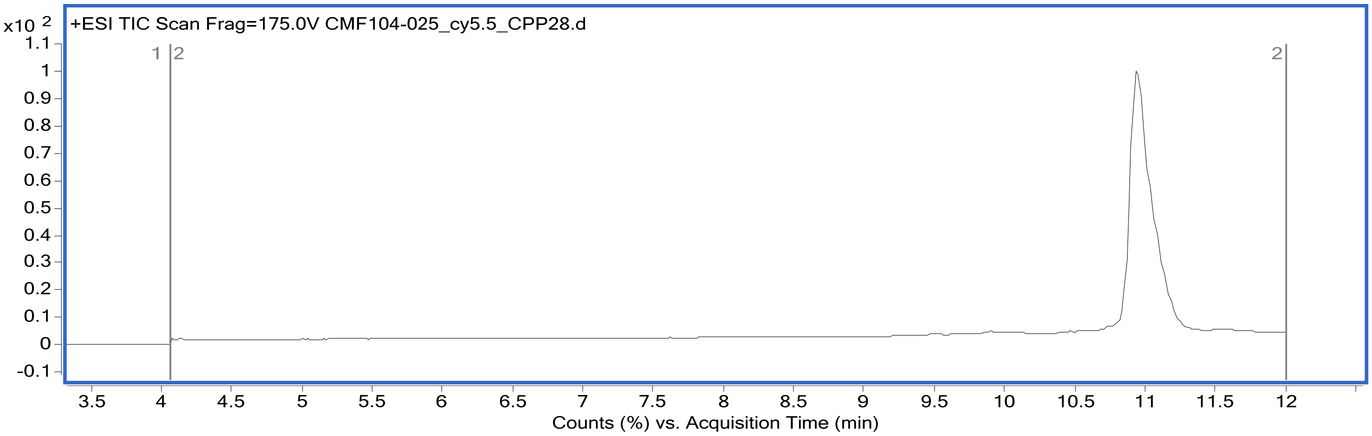

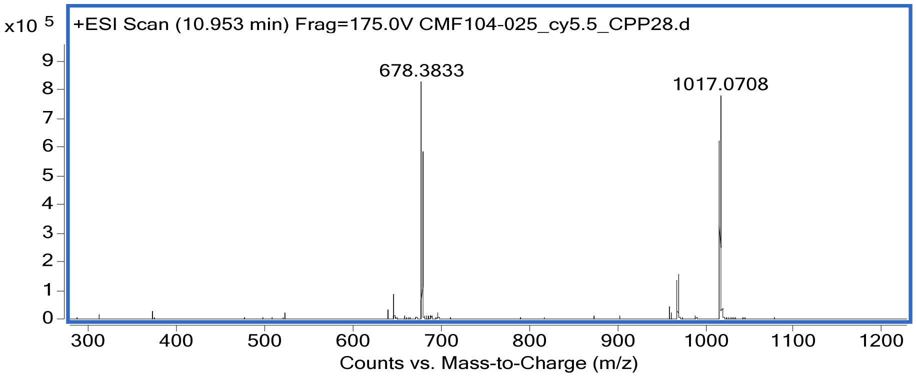

(28)

Peptide: $(PPR)_5$
Sequence: Cy5.5-triazole-PPRPPRPPRPPRPPR

Expected MW: 2513.4 Da
Observed MW: 2513.4 Da

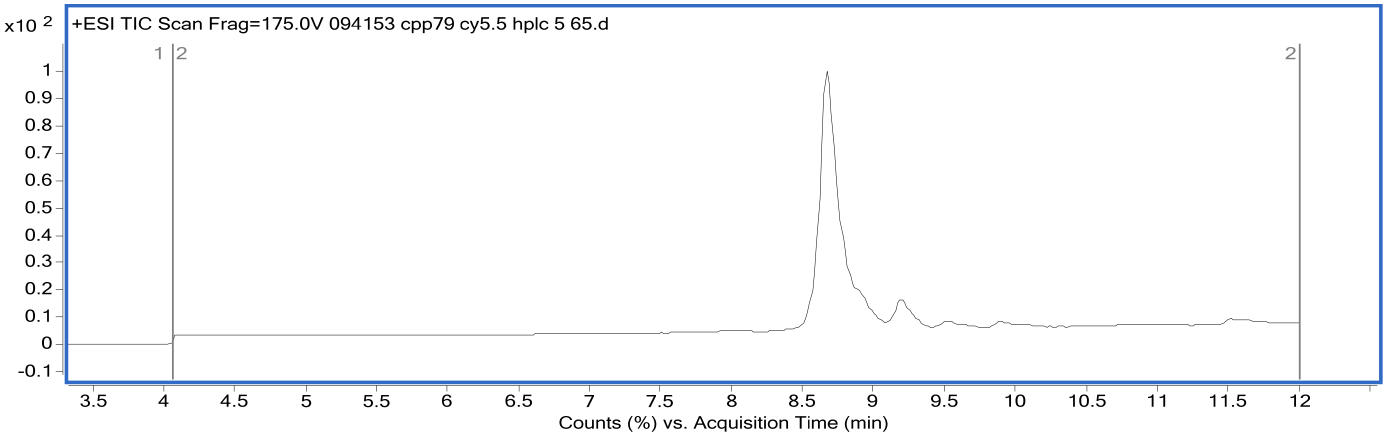

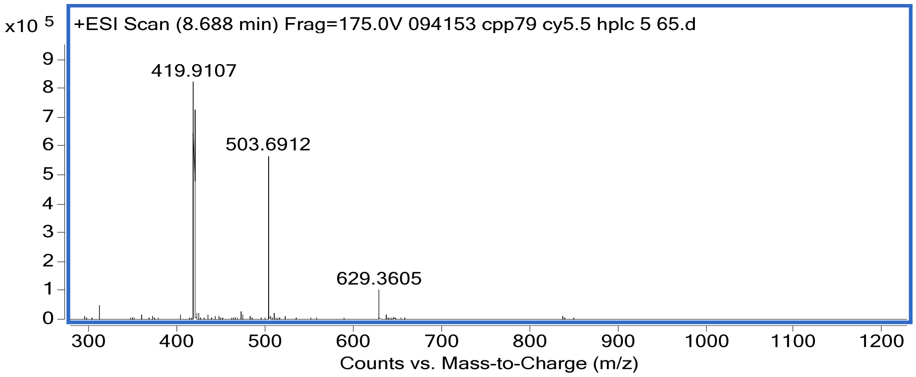

(29)

Peptide: (PPR)$_3$
Sequence: Cy5.5-triazole-PPRPPRPPR

Expected MW: 1813.0 Da
Observed MW: 1813.1 Da

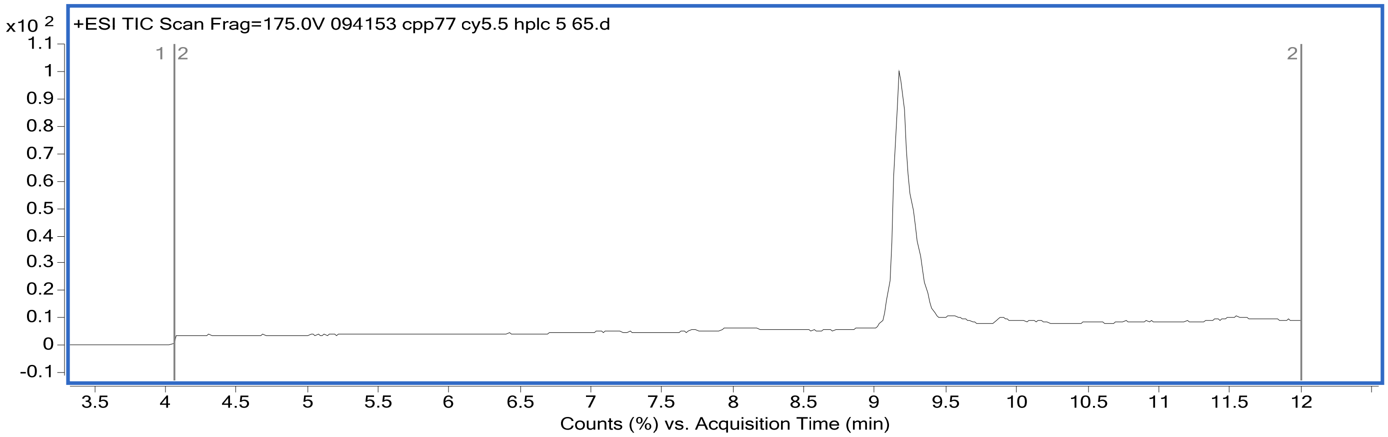

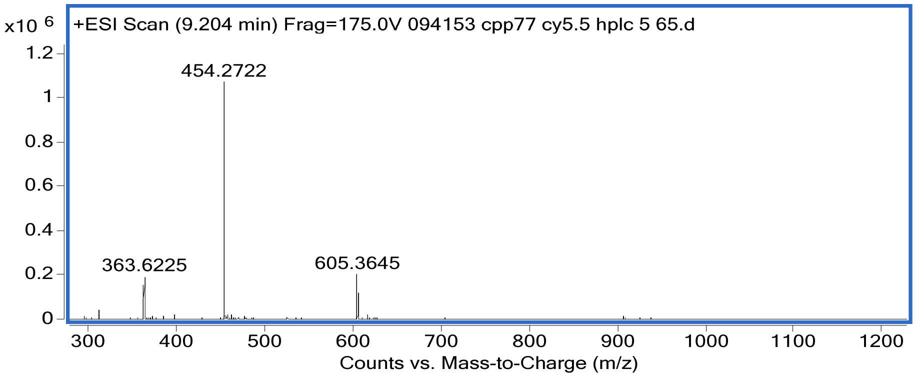

(30)

Peptide: SynB1 | Expected MW: 2843.5 Da
Sequence: Cy5.5-triazole-RGGRLSYSRRRFSTSTGR | Observed MW: 2843.5 Da

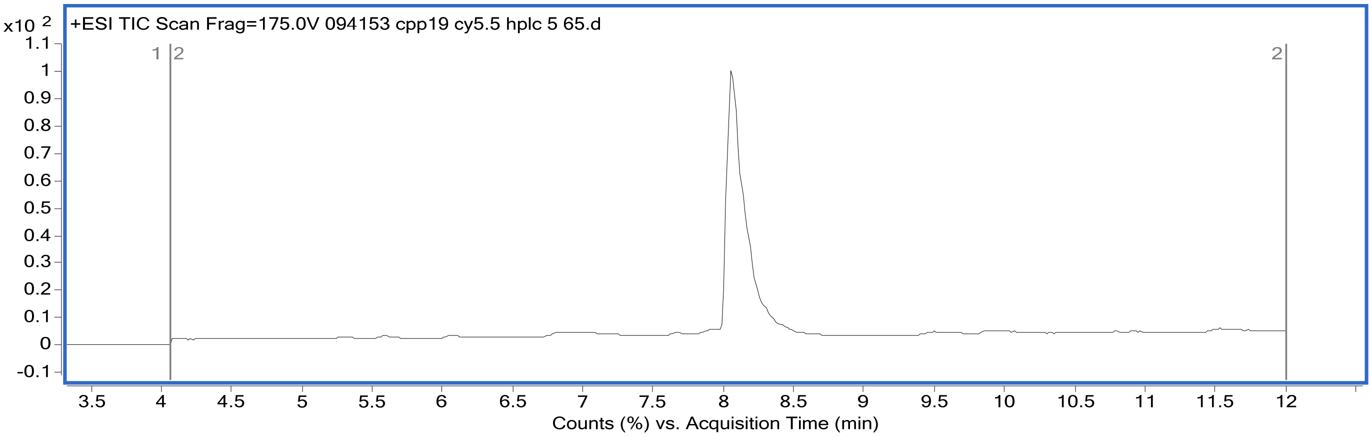

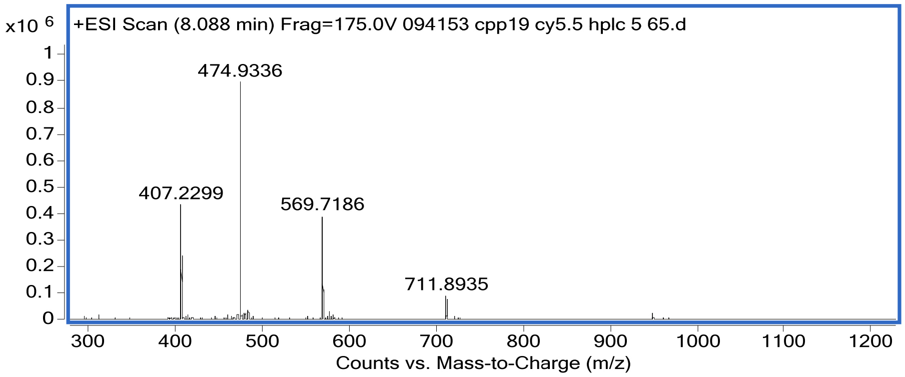

(31)

Peptide: Bim BH3
Sequence: Cy5.5-triazole-IWIAQELRRIGDEFNAYYARR

Expected MW: 3382.4 Da
Observed MW: 3382.9 Da

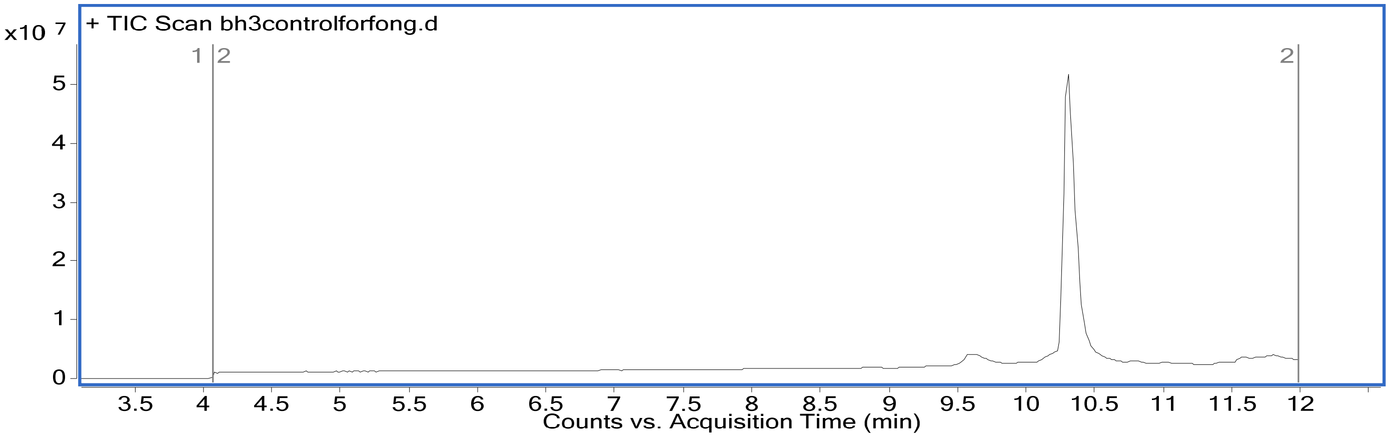

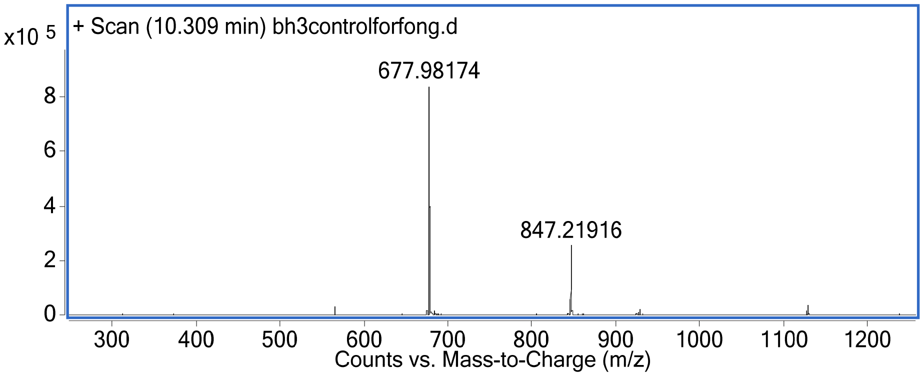

Supplementary Methods

Immunofluorescence staining cells

Each well of a Nunc Lab-Tek II thin-glass 8-well chambered coverglass (Thermo Scientific) was coated with poly-lysine (1 mg/mL in sterile water) overnight at 4°C. Astrocytes, HBVP, HBMEC and hCMEC/D3 were released by trypsin/EDTA, resuspended in their appropriate media and counted using a hemocytometer. Each well of the chambered coverglass was washed once with PBS. 5×10^4 of each cell type was seeded into each well, and allowed to adhere/grow overnight in a humidified incubator at 37°C with 5% CO_2. On the next day, all cells were washed once with PBS. HBVP and HBMEC were fixed in pre-chilled methanol for 10 mins on ice, while hCMEC/D3 and astrocytes were fixed in 3.7% formaldehyde for 10 mins at RT. All cells were washed twice in PBS and then, permeabilized with PBS containing 0.1% Tween-20 (v/v) for 20 mins. Cells were then, blocked with 5% normal goat serum (NGS) diluted in PBS containing 0.025% Tween-20 (v/v) for 1 hour at RT. The following primary anti-human antibodies: GFAP (astrocyte marker; 1:400 dilution), NG2 (pericyte marker; 1:300 dilution), CD31 (endothelial cell marker; 1:20 dilution) or VE-cadherin (endothelial cell marker; 1:400 dilution) were added into the appropriate wells, and allowed to incubated overnight at 4°C. Cells were then, washed 3× with PBS containing 0.025% Tween-20, and incubated appropriately with either the anti-mouse Alexa Fluor 488 or anti-rabbit Alexa Fluor 546 (Invitrogen) (1:1000 dilution) and Hoechst dye (Life Technologies) (1:1000 dilution) in 5% NGS diluted in PBS containing 0.025% Tween-20 (v/v) for 1 hour at RT. Cells were washed 3× with PBS containing 0.025% Tween-20 and imaged using a confocal microscope under a 20× or 40× oil immersion objective. Z-slices captured through each spheroid (up to 100 μm-deep) were merged to generate a 2D maximum intensity projection using the ZEN blue imaging software (version 2012).

Reproducibility of spheroid formation

Multicellular BBB spheroids were cultured as described in the main "Methods" section for 48 hours on 1% agarose in a 96-well plate. Spheroids were then inspected individually under an inverted microscope. "Acceptable" (round and intact spheroids) vs. "failed" (disrupted, undeveloped or broken spheroids) were visually determined according to Supplementary Figure 2. A sample size of 200 will provide sufficient precision, with a sufficiently narrow confidence interval, to estimate the proportion of spheroids that form properly, where the 95% confidence interval width around the proportion is 0.088

and the sample proportion is 0.90 (analyzed using the PASS 13 Power Analysis and Sample Size Software (2014)). A total of 886 multicellular BBB spheroids were analyzed, and the percentage of successful spheroid formation was counted.

Synthesis of angiopep-2, scrambled angiopep-2 and their derivatives

(Note: Scrambled angiopep-2 is referred to as "scramble")

TAMRA-angiopep-LPSTGG, TAMRA-scramble-LPSTGG, TAMRA-LPSTGG, G_5-angiopep, G_5-Bim BH3: The peptides synthesized using an automated flow-based synthesizer using Fmoc chemistry and H-Rink Amide-ChemMatrix resin as previously reported.[1] For all TAMRA-containing peptides, 5-TAMRA was coupled to the N-terminus on resin using HATU.

TAMRA-angiopep-affibody and TAMRA-affibody: TAMRA-angiopep-LPSTGG and TAMRA-LPSTGG were enzymatically coupled to G_5-affibody using sortase ($SrtA^*$), as previously described.[2] This affibody binds to HER-2 with nanomolar affinity.[3] G_5-affibody was expressed as SUMO-fusion constructs as previously described[4] and the SUMO-fusion was excised via SUMO protease and used without purification. For conjugation, 50 µM affibody, 1000 uM TAMRA-peptide, 5 µM $SrtA^*$, and Ni-NTA beads were mixed in sortase buffer (Tris·HCl (50 mM, pH 7.5), $CaCl_2$ (10 mM), NaCl (150 mM)) for 30 mins at RT with rocking. Ni-NTA beads bound unreacted starting material, $SrtA^*$-His_6, and His_6-SUMO. The supernatant (containing product) was collected and subjected to centrifugal filtration to separate excess peptide reactants. LC-MS was used to assess the purity and identity of the products.

TAMRA-angiopep-Bim BH3 and TAMRA-scramble-Bim BH3: An identical protocol was used for the sortase mediated conjugation of G_5-Bim BH3 to TAMRA-angiopep or TAMRA-scramble, except the product was purified by RP-HPLC on an Agilent Zorbax C_3 column (9.4 × 250 mm, 5 µm) using a water/acetonitrile (containing 0.1% TFA) gradient.

eGFP-angiopep: eGFP was conjugated to G_5-angiopep using sortase as described above, except the concentrations were 50 µM eGFP and 1000 µM G5-angiopep. The eGFP-LPSTGG-His_6 was expressed as a SUMO-fusion construct as previously described.[4] During the sortase-mediated conjugation, Ni-NTA beads were used to bind unreacted starting material, $SrtA^*$-His_6, and His_6-SUMO. The supernatant

(containing product) was collected and subjected to centrifugal filtration to separate excess peptide reactants. LC-MS was used to assess the purity and identity of the products.

Cy5-angiopep and Cy5-scramble: Before cleavage from the resin, the free N-termini of angiopep-2 and scrambled angiopep-2 were reacted with 2 mg of Cy5-NHS and 100 µL of DIEA in 2 mL of DMF. The peptides were then cleaved from the resin, and labeled peptide was separated from unlabeled using reverse phase HPLC on an Agilent Zorbax C_3 column (9.4 × 250 mm, 5 µm).

Transport of angiopep-2 derivatives

Multicellular BBB spheroids (established with astrocytes, HBVP and HBMEC) were established for 48 hrs as described in the main text, and pooled into a 1.5 mL Lobind microcentrifuge tube (Eppendorf) in 1.0 mL HBMEC working media. Angiopep-2, its derivatives and their respective controls (synthesis described in above section) labeled with a TAMRA dye was added into each tube at a final concentration of 5 µM for the Bim BH3 conjugates, and 2.5 µM for the affibody and GFP conjugates. Spheroids were incubated for 3 hrs at 37°C on a rotator. Spheroids were then washed with PBS, fixed with 3.7% formaldehyde and imaged using a confocal microscope. Confocal z-stack images (8 µm slices) were captured through each spheroid (104 µm-deep) using a 20× objective. The mean fluorescence intensity inside each spheroid at 88 µm depth was quantified using the ImageJ software and plotted using GraphPad Prism.

Supplementary References

1. Simon, M. D. *et al.* Rapid Flow-Based Peptide Synthesis. *ChemBioChem* **15,** 713–720 (2014).
2. Liao, X., Rabideau, A. E. & Pentelute, B. L. Delivery of Antibody Mimics into Mammalian Cells via Anthrax Toxin Protective Antigen. *ChemBioChem* **15,** 2458–2466 (2014).
3. Orlova, A. *et al.* Tumor imaging using a picomolar affinity HER2 binding affibody molecule. *Cancer Res.* **66,** 4339–4348 (2006).
4. Policarpo, R. L. *et al.* Flow-Based Enzymatic Ligation by Sortase A. *Angew. Chem. Int. Ed.* **53,** 9203–9208 (2014).
